# Synthesis, Characterization, and Anticancer Activities Evaluation of Compounds Derived from 3,4-Dihydropyrimidin-2(1*H*)-one

**DOI:** 10.3390/molecules24050891

**Published:** 2019-03-03

**Authors:** Ye Liu, Jiaqi Liu, Renmei Zhang, Yan Guo, Hongbo Wang, Qingguo Meng, Yuan Sun, Zongliang Liu

**Affiliations:** 1 School of Pharmacy, Key Laboratory of Molecular Pharmacology and Drug Evaluation (Yantai University), Ministry of Education, Collaborative Innovation Center of Advanced Drug Delivery System and Biotech Drugs in Universities of Shandong, Yantai University, Yantai 264005, China; liuyeer126@126.com (Y.L.); jiaqi.miami@gmail.com (J.L.); zhangrenmeigrace@126.com (R.Z.); 18865672173@163.com (Y.G.); hongbowangyt@163.com (H.W.); qinggmeng@163.com (Q.M.); 2 Department of Biochemistry and Molecular Medicine, School of Medicine, University of California Davis, Sacramento, CA 95817, USA; ysysun@ucdavis.edu

**Keywords:** 3,4-dihydropyrimidin-2(1*H*)-ones, N^1^-alkylation, structure-activity relationship, in vivo experiments, anticancer activities

## Abstract

3,4-dihydropyrimidin-2(1*H*)-one compounds (DHPMs) possess extensive biological activities and are mainly prepared via Biginelli reaction and N-alkylation. In the present study, selective alkylation of N^1^ was investigated by using tetrabutylammonium hydroxide. In vitro cytotoxicity study on all synthesized compounds demonstrated that introduction of the aryl chain in the R^3^ as well as the low electron-donating group in the R^1^ of DHPMs contributed to the anti-proliferative potency. A larger value of the partition coefficient (Log P) and suitable polar surface area (PSA) values were both found to be important in order to maintain the antitumor activity. The results from in vivo study indicated the great potential of compound **3d** to serve as a lead compound for novel anti-tumor drugs to treat glioma. Pharmacophore study regarding the structure-activity relations of DHPMs were also conducted. Our results here could provide a guide for the design of novel bioactive 3,4-dihydropyrimidin-2(1*H*)-one compounds.

## 1. Introduction

Noncommunicable diseases (NCDs) are a major threat to global health, causing a significant amount of death every year. Second only to cardiovascular disease, cancer is becoming a global burden which lead to an estimation of 8.7 million deaths in 2015 [1]. Moreover, cancer is expected to rank as the leading cause of death and the single most significant barrier for the increase of life expectancy in every country worldwide [2]. Contrary to common misperception, cancer is a major health challenge not only in high-income countries but also in low- and middle-income countries (LMICs), where the number of cancer occurrence is rapidly growing [3]. Unfortunately, almost all anticancer drugs are associated with serious side effects, making the search for novel chemical agents that are cytotoxic to cancer cells with less side effects an urgent need.

**Figure 1:**
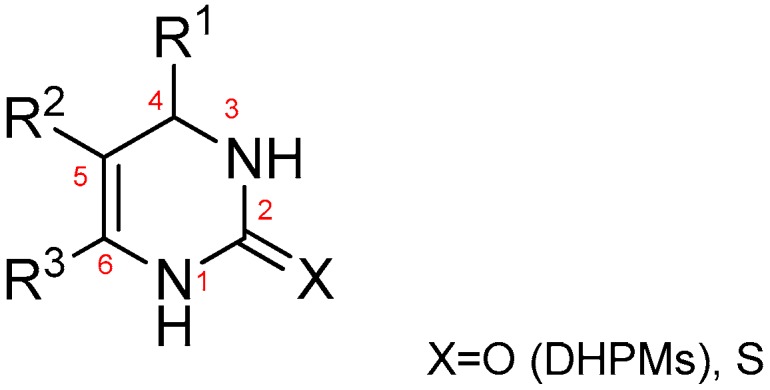
The structure of a 3,4-dihydropyrimidin-2(1H)-one compound (DHPM) and its thione derivatives.

The interests of using DHPMs in medicinal chemistry is dramatically growing (Figure 1) due to their therapeutic and pharmacological properties [4,5]. It has been reported that DHPMs can possess various biological activities including antiviral [6,7], antitumor [8], anti-inflammatory [9], antidiabetic [10], antibacterial [11], antifungal [12], anti-epileptic [13], antimalarial [14], and antileishmanial [15] and others upon suitable structural modification. The highly functionalized DHPM 10, termed MAL3-101, had been observed with effect of inducing breast cancer cell apoptosis [16]. More recently, DHPMs have emerged as the integral backbone of several calcium channel blockers [17,18], antioxidant molecules [19], and radical scavengers [20,21,22]. In addition, Barbosa et al. reported the synthesis and biological evaluation of a series of DHPMs functionalized with selenocyanides as potential multi-targeted therapeutics against Alzheimer’s disease (AD) [23].

In DHPMs, R^1^ is usually an aryl group, such as phenyl or pyridyl, while R^2^ is an ester or amide group and R^3^ is an alkyl group, such as methyl or ethyl. General method for the synthesis of DHPMs starts with firstly obtaining the basic scaffold via Biginelli reaction followed by N alkylation. Mohammadi and Behbahani reviewed the synthesis of DHPMs and improved procedures for the preparation of DHPMs under solvent-free conditions or with the presence of solvent [24]. Dallinger and Kappe introduced a selective N^1^-alkylation method of DHPMs using Mitsunobu reaction [25]. However, the yield of Mitsunobu reaction was low, and the reagents were relatively expensive, making it not suitable for practical synthesis. Singh et al. provided another N-alkylation method catalyzed by inorganic strong base [26]. In the present report, not only did we find highly selective N^1^-alkylation of DHPMs in the presence of tetrabutylammonium hydroxide, but also we investigated the biological importance of the newly synthesized molecules both in vitro and in vivo.

## 2. Results and Discussion

### 2.1. Chemistry

The various non-alkylated DHPM moieties used in this report were synthesized through one pot Biginelli condensation reaction according to the reported method [27]. The effects of different choices of bases on the reaction, including sodium hydride (NaH), lithium hydroxide (LiOH·H_2_O), potassium carbonate (K_2_CO_3_) and some pKa similar organic base, were carried out to perform N^1^ and N^3^ dialkylation of DHPMs. While using a strong base, such as LiOH·H_2_O, NaH and potassium tert-butoxide (KTB), the reactions were proceeding very fast. However the dialkyl product was formed and detected even from the beginning of the reaction. In some reactions the yields of dialkylation were even higher than that of N^1^-alkylation. N-alkylation cannot be achieved when a weak base is used, such as K_2_CO_3_, triethylamine (Et_3_N), 1,8-diazabicyclo[5,4,0]undec-7-ene (DBU) and tetramethylguanidine (TMG). Interestingly, when tetrabutylammonium hydroxide was selected as the base, the yield was similar to that of cesium carbonate (Cs_2_CO_3_), while no dialkyl products was found (Table S1 in Supplementary Materials). A possible explanation for this phenomenon is that N^3^-alkylation of DHPMs had a large steric effect, so the steric-hindered base like tetrabutylammonium hydroxide would favor the mono-alkylation reaction. The yields of the DHPMs were reported in Table 1.

### 2.2. Structure–Activity Relationship (SAR) Studies

#### 2.2.1. Cytotoxicity Activities with SAR

Cytotoxic activity of the DHPMs are strongly dependent on their structure. Yadlapalli et al. screened 21 compounds in vitro anticancer screening against MCF-7 human breast cancer cells, and found the maximum GI_50_ was 33.2 μM. The results indicated that presence of thio-urea functional group in DHPMs enhanced the in vitro anticancer activity [28]. In vitro cytotoxicity of all synthesized compounds containing X = O were assayed on four cell lines, namely U87, U251 human malignant glioma cell lines, HeLa human cervical cancer cell line and A549 human lung cancer cell line. Cancer cell lines were exposed to drug solution at concentration of 10 µM for 72 h, and results were summarized in Table 2. On the whole, no certain trend in inhibitory activity was observed in Hela and A549 cell lines, indicating that these compounds were selective toward certain tumor types. Some of the tested compounds showed effective cytotoxicity in U87 and U251 cell lines.

Table 2 showed that compound **1a** resulted in cell viability of 87.34 ± 1.24 and 67.14 ± 4.61 in U87 and U251 cell lines. For SAR studies, we maintained the R^1^ as Br, R^2^ as ethyl acetate and explored R^3^ first with a series of halohydrocarbons. Compounds **1b**, **1f**, **1g**, and **1i**, with an alkyl side chain replacing the methyl 4-bromobutanoate of **1a**, were found to have similar activity as **1a**. Compound **1h**, with an 1-bromohexane in R^3^, displayed decent activity, indicating that the length of the alkyl side chain in the R^3^ would affect the potency. Compound **1d**, with a 4-bromobenzyl group in the R^3^, also demonstrated strong cytotoxicity, suggesting that the R^3^ may tolerate variations to some degree. We then explored the R^2^ with the goal to compare the ester group and the amide group on cell viability. Compared with **1e**, compound **7e** and **8e** differ in R^2^, had no significant change in activity. Compounds **7c**, **7d**, **7f**, **8a**, and **8d** were also tested, and not satisfactory performances were observed, suggesting that the amide group may not be compatible in that position. For R^1^ of DHPMs, we proposed that the capability of electron-donating group may affect the cell viability and the SAR of the R^1^ group was explored. Compound **3a**, with a low electron-donating 4-biphenyl group instead of 4-phenylmorpholine group or 4-methoxyphenyl, was found to have better potency than **4a** and **2a**. Compound **1a**, with the 4-bromo phenyl group in the R^1^, had significant activity in U251 cell lines, suggesting that a low electron-withdrawing group in the R^1^ may contribute to augment the activity. Compound **5a** and **6a**, with a 4-nitrophenyl group and 4-pyridinylphenyl group in the R^1^, were also not active in the cell study, suggesting that high electron-withdrawing substituent in the R^1^ may not be tolerated. Studies on different electron-donating groups in the R^1^ had shown that a maximum cytotoxic activity may be achieved for low electron-donating ability or low electron-withdrawing ability. Based on the above studies, the cell viability of compounds **3c**, **3d**, **3e**, **3g**, and **3h** were tested and compound **3d** and **3g**, with 4-biphenyl low electron-donating group in the R^1^ and alkyl chain or 4-bromobenzyl in the R^3^, were found to have good activity.

#### 2.2.2. Half Maximal Inhibitory Concentration (IC_50_) Study of Compounds **1d**, **1h**, **3d** and **3g**

The values of 50% inhibitory concentration (concentration of drug yielding a 50% cell viability decrease, IC_50_) measured for the distinct compounds investigated were comprised in Table 3, which confirmed that the active compounds can inhibit tumor cell growth. In tumor cells, inhibition of heat shock protein 90 (HSP90) results in the degradation of oncoproteins which is crucial to malignant progression [29]. Preclinical data suggest that synthetic HSP90 inhibitors such as BIIB021 may be active against tumors with acquired multidrug resistance [30]. All of the tested compounds had IC_50_ in the micromolar range against U87 and U251 cell lines. These results evidenced that although compounds **1d** (9.72 ± 0.29 µM in U87 cell line, 13.91 ± 0.86 µM in U251 cell line), **1h** (9.3 ± 0.81 µM in U87 cell line, 14.01 ± 0.76 µM in U251 cell line), **3d** (12.02 ± 0.5 µM in U87 cell line, 6.36 ± 0.73 µM in U251 cell line), and **3g** (9.52 ± 0.81 µM in U87 cell line, 7.32 ± 0.86 µM in U251 cell line) did not display stronger cytotoxic activity on the U87 and U251 cell lines compared to positive control, they can still possess certain cytotoxic activity in micromolar range. In the present study, it was verified that the alkyl chain or aryl chain in the R^3^, and low electron-donating ability or low electron-withdrawing ability in the R^1^ displayed obvious effect. As expected, all of the four compounds especially **3d** displaying high Log P (5.01) values and suitable PSA (58.64).

#### 2.2.3. Effects on Xenograft Model of Compounds **3d** and **3g**

**Figure 2:**
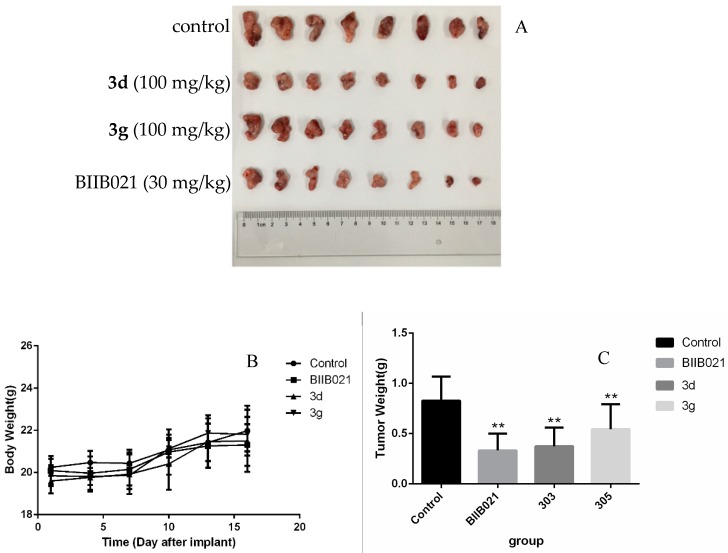
Effects of compounds **3d** and **3g** on the antitumor activity of GL261 xenograft tumors in C57 mice. (**A**): The tumors were stripped and photographed the experimental results after two weeks. (**B**): The body weight of the four groups of mice was changed for two weeks. (**C**): The tumor of the four groups of mice was weighed after two weeks. ** *p* < 0.01, compared with control group.

We further investigated the efficacy of compounds **3d** and **3g** in xenograft tumor model on the basis of their good membrane permeability and IC_50_ value. In brief, GL261 mouse malignant glioma cells were inoculated subcutaneously in right frank regions. Mice were treated with either: control, positive control (30 mg/kg), or compound **3d** (100 mg/kg), or compound **3g** (100 mg/kg). The results of representative studies were summarized in Table 4, and examples were shown in Figure 2. These data indicated that in xenograft tumor model, compound **3d** or **3g** were able to significantly inhibit tumor growth, with inhibition ratios (IR) of 54.9% and 34.3%, respectively. The compound **3d** produced a similar antitumor activity compared with BIIB021 (IR 59.7%). This study had shown that the aryl chain in the R^3^, and 4-biphenyl low electron-donating group in the R^1^ had a moderate growth inhibitory effected on xenograft tumor model. The compound **3d** also had suitable Log P and PSA values. Although the compounds were less active when compared to the positive control, compound **3d** had the potential to serve as lead compound and be further optimized to improve activity.

### 2.3. Pharmacophore Requirements

**Figure 3:**
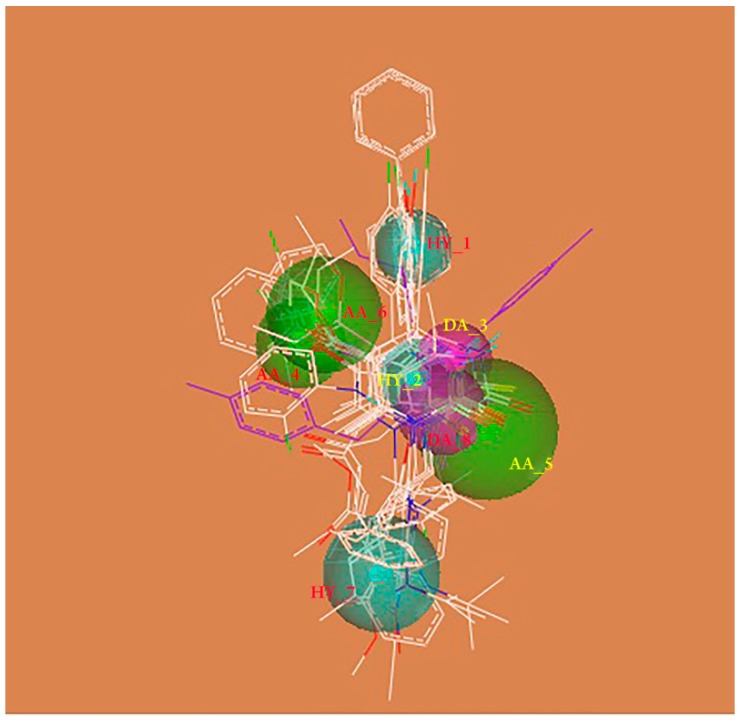
Pharmacophore Requirements.

According to previous studies, thirteen substituted DHPMs with good anticancer activities were selected to generate pharmacophores and guide the design of novel DHPMs derivatives (Table S3 in Supplementary Materials). Galahad module of Sybyl-X 2.0 (Certara, Princeton, NJ, USA) was used to generate pharmacophore using population size of 20 and maximum generations as 10. Finally, 13 models were generated (Figure 3). The best pharmacophore model was chosen with low energy and high value of steric and hydrogen bonding. Eight pharmacophoric features, namely three acceptor atoms (AA-4, 5, 6), two donor atoms (DA-3, 8) and three hydrophobic center (HY-1, 2, 7) were identified. The two acceptor atoms were at the R^2^ position and X position. Hydrophobic center is the DHPMs parent ring, R^1^ position and R^3^ position.

## 3. Materials and Methods

### 3.1. General Information

All reagents were obtained from commercial suppliers and were used without any further purification unless otherwise stated. Column chromatography was performed using an SRL silica gel (200–300 mesh). Thin layer chromatography was performed using Merck silica gel GF_254_ plates. Melting points were measured on an XT3A micro-melting point apparatus and are uncorrected (Beijing Keyi Company, Beijing, China). ^1^H NMR and ^13^C NMR spectra were recorded with a Bruker AV-400 instrument or a Bruker AV-300 (Bruker, Ettlingen, Germany). Chemical shifts were reported as δ values (ppm) from internal reference tetramethylsilane (TMS). All coupling constants were reported in hertz (Hz), and proton multiplicities were labeled as br (broad), s (singlet), d (doublet), dd (doublet of doublets), t (triplet), q (quartet), and m (multiplet). HR-MS were performed on a Waters Vion IMS Q-tof (Waters, MA, USA).

### 3.2. Synthesis and the General Procedure for N1-alkylation

Tetrabutylammonium hydrogen sulfate (TBAS 0.1 eq) was added to a solution of ethyl 4-(4-bromophenyl)-6-methyl-2-oxo-1, 2, 3, 4-tetrahydropyrimidine-5-carboxylate (200.0 mg, 0.59 mmol) and tetrabutylammonium hydroxide (0.26 mL, 1.0 mmol) in anhydrous DMF (4.0 mL). The mixture was stirred at 45 °C for 1.5 h under anhydrous condition. Then 2-chlorobenzyl chloride (0.13 mL, 1.06 mmol), potassium iodide (0.1 eq) were added to the reaction mixture slowly and stirred at 45 °C for 16 h. Saturated NaCl solution (25 mL) was added and then the reaction mixture was extracted with ethyl acetate (3 × 25 mL). The combined organic layer was washed with water (2 × 50 mL), followed by brine solution (1 × 50 mL). The organic layer was dried over anhydrous Na_2_SO_4_ and the solvent was removed under reduced pressure. Column chromatographic purification using a methanol in dichloromethane gradient (dichloromethane: methanol = 60:1–5:1) yielded compounds **1a**–**8e**.

#### 3.2.1. N^1^-Substituted Ethyl4-(4-bromophenyl)-6-methyl-2-oxo-1,2,3,4-tetrahydropyrimidine-5-carboxylates **1a**–**1j**

*Ethyl 4-(4-bromophenyl)-1-(4-methoxy-4-oxobutyl)-6-methyl-2-oxo-1,2,3,4-tetrahydropyrimidine-5-carboxylate* (**1a**). White solid, m.p.: 92.4–95.0 °C, yield: 8.0%, *R*f value: 0.5 (CH_2_Cl_2_: CH_3_OH=20:1). ^1^H NMR (400 MHz, CDCl_3_) δ 7.44 (d, *J* = 8.4 Hz, 2H, 2 × Ar-H), 7.14 (d, *J* = 8.4 Hz, 2H, 2 × Ar-H), 5.53 (s, 1H, NH), 5.35 (d, *J* = 2.9 Hz, 1H, Ar-CH), 4.12 (q, *J* = 7.1 Hz, 2H, OCH_2_), 3.98–3.88 (m, 1H, NCH_2_), 3.71 (d, *J* = 5.3 Hz, 3H, OCH_3_), 3.66 (dd, *J* = 9.5, 5.1 Hz, 1H, NCH_2_), 2.57 (s, 3H, =CCH_3_), 2.35–2.24 (m, 2H, COCH_2_), 2.00–1.77 (m, 2H, CH_2_), 1.20 (t, *J* = 7.1 Hz, 3H, CH_3_). ^13^C NMR (101 MHz, DMSO-*d_6_*) δ 173.35 (C=O), 165.98 (C=O), 153.11 (C=O), 150.50 (Ar-C), 143.74 (C-N), 131.86 (2 × Ar-C), 128.82 (2 × Ar-C), 120.95 (Br-Ar-C), 103.30 (C=C), 60.24 (CH_2_), 52.28 (CH), 51.88 (CH_3_), 41.43 (CH_2_), 30.67 (CH_2_), 24.98 (CH_2_), 16.08 (CH_3_), 14.56 (CH_3_). HRMS (ESI): *m*/*z* calcd for C_19_H_23_BrN_2_O_5_ [M + H]^+^ 439.0869, found 439.0868.

*Ethyl 4-(4-bromophenyl)-1-(3-ethoxy-3-oxopropyl)-6-methyl-2-oxo-1,2,3,4-tetrahydropyrimidine-5-carboxylate* (**1b**). White solid, m.p.: 126.8–127.0 °C, yield: 52.2%, *R*f value: 0.6 (CH_2_Cl_2_: CH_3_OH=20:1). ^1^H NMR (400 MHz, CDCl_3_) δ 7.48–7.42 (m, 2H, 2 × Ar-H), 7.25 (d, *J* = 8.4 Hz, 2H, 2 × Ar-H), 5.37 (s, 1H, Ar-CH), 4.19–4.05 (m, 4H, 2 × OCH_2_), 3.83–3.73 (m, 1H, NCH_2_), 3.29–3.20 (m, 1H, NCH_2_), 2.70 (d, *J* = 16.4 Hz, 1H, CH_2_), 2.49–2.38 (m, 1H, CH_2_), 2.33 (s, 3H, =CCH_3_), 1.25 (q, *J* = 7.3 Hz, 6H, 2 × CH_3_). ^13^C NMR (101 MHz, DMSO-*d_6_*) δ 171.71 (C=O), 165.37 (C=O), 152.05 (C=O), 148.21 (Ar-C), 142.66 (C-N), 132.08 (2 × Ar-C), 129.58 (2 × Ar-C), 121.40 (Br-Ar-C), 100.21 (C=C), 60.57 (2 × CH_2_), 59.97 (CH_2_, CH), 33.09 (CH_2_), 18.10 (CH_3_), 14.54 (2 × CH_3_). HRMS (ESI): *m*/*z* calcd for C_19_H_23_BrN_2_O_5_ [M + H]^+^ 439.0869, found 439.0870.

*Ethyl 4-(4-bromophenyl)-1-(2-chlorobenzyl)-6-methyl-2-oxo-1,2,3,4-tetrahydropyrimidine-5-carboxylate* (**1c**). White solid, m.p.: 125.6–127.8 °C, yield: 35.1%, *R*f value: 0.5 (CH_2_Cl_2_: CH_3_OH=20:1). ^1^H NMR (400 MHz, CDCl_3_) δ 7.41 (d, *J* = 8.3 Hz, 2H, 2 × Ar-H), 7.36 (d, *J* = 7.7 Hz, 1H, Ar-H), 7.26 (s, 1H, Ar-H), 7.16 (d, *J* = 8.2 Hz, 3H, 3 × Ar-H), 6.90 (d, *J* = 7.4 Hz, 1H, Ar-H), 6.48 (s, 1H, NH), 5.44 (s, 1H, Ar-CH), 5.06 (d, *J* = 7.8 Hz, 2H, NCH_2_), 4.11 (q, *J* = 7.1 Hz, 2H, OCH_2_), 2.38 (s, 3H, =CCH_3_), 1.18 (t, *J* = 7.1 Hz, 3H, CH_3_). ^13^C NMR (101 MHz, DMSO-*d_6_*) δ 165.33 (C=O), 152.34 (C=O), 148.46 (2 × Ar-C), 141.90 (C-N), 134.59 (Ar-C-Cl), 132.88 (2 × Ar-C), 132.15 (2 × Ar-C), 130.04 (Ar-C), 129.59 (Ar-C), 129.34 (Ar-C), 128.00 (Ar-C), 121.56 (Br-Ar-C), 100.15 (C=C), 60.01 (CH_2_), 59.16 (CH_2_, CH), 18.21 (CH_3_), 14.53 (CH_3_). HRMS (ESI): *m*/*z* calcd for C_21_H_20_BrClN_2_O_3_ [M + H]^+^ 463.0424, found 463.0439.

*Ethyl 1-(4-bromobenzyl)-4-(4-bromophenyl)-6-methyl-2-oxo-1,2,3,4-tetrahydropyrimidine-5-carboxylate* (**1d**). White solid, m.p.: 190.4–191.9 °C, yield: 32.3%, *R*f value: 0.7 (CH_2_Cl_2_: CH_3_OH=20:1). ^1^H NMR (400 MHz, CDCl_3_) δ 7.44 (d, *J* = 8.3 Hz, 4H, 4 × Ar-H), 7.13 (d, *J* = 8.4 Hz, 2H, 2 × Ar-H), 7.01 (d, *J* = 8.3 Hz, 2H, 2 × Ar-H), 5.72 (s, 1H, NH), 5.42 (d, *J* = 2.6 Hz, 1H, Ar-CH), 5.10 (d, *J* = 16.7 Hz, 1H, NCH_2_), 4.87 (d, *J* = 16.3 Hz, 1H, NCH_2_), 4.12 (q, *J* = 7.1 Hz, 2H, OCH_2_), 2.44 (s, 3H, =CCH_3_), 1.19 (t, *J* = 7.1 Hz, 3H, CH_3_). ^13^C NMR (101 MHz, DMSO-*d_6_*) δ 165.90 (C=O), 153.29 (C=O), 150.16 (Ar-C), 143.76 (C-N), 138.63 (Ar-C), 131.90 (4 × Ar-C), 128.98 (4 × Ar-C), 121.06 (Br-Ar-C), 120.43 (Br-Ar-C), 103.75 (C=C), 60.33 (OCH_2_), 52.53 (CH-NH), 45.05 (N-CH_2_), 16.54 (CH_3_), 14.53 (CH_3_). HRMS (ESI): *m*/*z* calcd for C_21_H_20_Br_2_N_2_O_3_ [M + H]^+^ 508.9898, found 508.9901.

*Ethyl 4-(4-bromophenyl)-1-(4-methoxybenzyl)-6-methyl-2-oxo-1,2,3,4-tetrahydropyrimidine-5-carboxylate* (**1e**). White solid, m.p.: 136.8–139.7 °C, yield: 43.6%, *R*f value: 0.5 (CH_2_Cl_2_: CH_3_OH=20:1). ^1^H NMR (400 MHz, CDCl_3_) δ 7.43–7.37 (m, 2H, 2×Ar-H), 7.11 (d, *J* = 8.4 Hz, 2H, 2 × Ar-H), 7.05 (d, *J* = 8.6 Hz, 2H, 2 × Ar-H), 6.86–6.80 (m, 2H, 2 × Ar-H), 5.75 (s, 1H, NH), 5.39 (d, *J* = 2.9 Hz, 1H, Ar-CH), 5.16 (d, *J* = 15.9 Hz, 1H, NCH_2_), 4.78 (d, *J* = 15.8 Hz, 1H, NCH_2_), 4.09 (q, *J* = 7.1 Hz, 2H, OCH_2_), 3.81 (s, 3H, OCH_3_), 2.48 (s, 3H, =CCH_3_), 1.18 (t, *J* = 7.1 Hz, 3H, CH_3_). ^13^C NMR (101 MHz, DMSO-*d_6_*) δ 165.95 (C=O), 158.70 (C=O), 153.42 (Ar-C), 150.43 (Ar-C), 143.86 (C-N), 131.83 (2 × Ar-C), 130.91 (2 × Ar-C), 128.99 (2 × Ar-C), 128.15 (Ar-C), 120.98 (Br-Ar-C), 114.41 (2×Ar-C), 103.66 (C=C), 60.27 (CH_2_), 55.68 (CH_3_), 55.44 (CH), 52.49 (CH_2_), 16.55 (CH_3_), 14.53 (CH_3_). HRMS (ESI): *m*/*z* calcd for C_22_H_23_BrN_2_O_4_ [M + H]^+^ 459.0919, found 459.0917.

*Ethyl 4-(4-bromophenyl)-6-methyl-2-oxo-1-propyl-1,2,3,4-tetrahydropyrimidine-5-carboxylate* (**1f**). White solid, m.p.: 114.7–115.3 °C, yield: 20.0%, *R*f value: 0.3 (CH_2_Cl_2_: CH_3_OH=20:1). ^1^H NMR (400 MHz, CDCl_3_) δ 7.45 (d, *J* = 8.4 Hz, 2H, 2 × Ar-H), 7.15 (d, *J* = 8.4 Hz, 2H, 2 × Ar-H), 5.59 (s, 1H, NH), 5.36 (d, *J* = 2.8 Hz, 1H, Ar-CH), 4.12 (q, *J* = 7.1 Hz, 2H, OCH_2_), 3.88 (td, *J* = 9.9, 9.4, 5.0 Hz, 1H, NCH_2_), 3.56 (ddd, *J* = 14.7, 9.9, 5.5 Hz, 1H, NCH_2_), 2.54 (s, 2H, =CCH_3_), 1.69–1.54 (m, 2H, CH_2_), 1.27 (t, *J* = 7.1 Hz, 1H, OCH_2_), 1.21 (t, *J* = 7.1 Hz, 2H, OCH_2_), 0.92 (t, *J* = 7.4 Hz, 3H, CH3). ^13^C NMR (101 MHz, DMSO-*d_6_*) δ 167.36 (C=O), 154.39 (C=O), 151.87 (Ar-C), 145.26 (C-N), 133.17 (2 × Ar-C), 130.18 (2 × Ar-C), 122.22 (Br-Ar-C), 104.32 (C=C), 61.50 (OCH_2_), 53.73 (CH-NH), 45.12 (N-CH_2_), 24.38 (CH_2_), 17.51 (CH_3_), 15.90 (CH_3_), 12.72 (CH_3_). HRMS (ESI): *m*/*z* calcd for C_17_H_21_BrN_2_O_3_ [M + H]^+^ 381.0814, found 381.0807.

*Ethyl 4-(4-bromophenyl)-1-butyl-6-methyl-2-oxo-1,2,3,4-tetrahydropyrimidine-5-carboxylate* (**1g**). White solid, m.p.: 117.5–119.3 °C, yield: 69.7%, *R*f value: 0.3 (CH_2_Cl_2_: CH_3_OH=20:1). ^1^H NMR (400 MHz, CDCl_3_) δ 7.42 (d, *J* = 8.4 Hz, 2H, 2 × Ar-H), 7.13 (d, *J* = 8.4 Hz, 2H, 2 × Ar-H), 5.39 (s, 1H, NH), 5.33 (s, 1H, Ar-CH), 4.10 (q, *J* = 7.1 Hz, 2H, OCH_2_), 3.90 (ddd, *J* = 15.1, 9.9, 5.9 Hz, 1H, NCH_2_), 3.58 (ddd, *J* = 14.7, 9.6, 5.4 Hz, 1H, NCH_2_), 2.52 (s, 3H, =CCH_3_), 1.64 (s, 2H, CH_2_), 1.30 (dd, *J* = 14.9, 7.4 Hz, 2H, CH_2_), 1.18 (t, *J* = 7.1 Hz, 3H, CH_3_), 0.92 (t, *J* = 7.3 Hz, 3H, CH_3_). ^13^C NMR (101 MHz, DMSO-*d_6_*) δ 166.02 (C=O), 153.12 (C=O), 150.58 (Ar-C), 143.92 (C-N), 131.81 (2 × Ar-C), 128.94 (Ar-C), 128.72 (Ar-C), 120.89 (Br-Ar-C), 103.17 (C=C), 60.18 (OCH_2_), 52.30 (CH-NH), 31.90 (N-CH_2_), 19.89 (CH_2_), 16.16 (CH_3_), 14.57 (CH_3_), 14.10 (CH_3_). HRMS (ESI): *m*/*z* calcd for C_18_H_23_BrN_2_O_3_ [M + H]^+^ 395.0970, found 395.0977.

*Ethyl 4-(4-bromophenyl)-1-hexyl-6-methyl-2-oxo-1,2,3,4-tetrahydropyrimidine-5-carboxylate* (**1h**). White solid, m.p.: 161.3–161.6 °C, yield: 55.8%, *R*f value: 0.5 (CH_2_Cl_2_: CH_3_OH=20:1). ^1^H NMR (400 MHz, CDCl_3_) δ 7.42 (d, *J* = 8.1 Hz, 2H, 2 × Ar-H), 7.13 (d, *J* = 8.2 Hz, 2H, 2 × Ar-H), 5.50 (s, 1H, NH), 5.33 (s, 1H, Ar-CH), 4.10 (q, *J* = 7.1 Hz, 2H, OCH_2_), 3.90 (ddd, *J* = 14.8, 9.6, 5.9 Hz, 1H, NCH_2_), 3.57 (ddd, *J* = 14.7, 9.6, 5.4 Hz, 1H, NCH_2_), 2.52 (s, 3H, =CCH_3_), 1.59 (s, 1H, CH_2_), 1.49 (s, 1H, CH_2_), 1.27 (s, 6H, 3 × CH_2_), 1.19 (t, *J* = 7.1 Hz, 3H, CH_3_), 0.88 (t, *J* = 6.6 Hz, 3H, CH_3_). ^13^C NMR (101 MHz, DMSO-*d_6_*) δ 166.04 (C=O), 153.20 (C=O), 150.68 (Ar-C), 143.84 (C-N), 131.79 (2 × Ar-C), 128.78 (2 × Ar-C), 120.91 (Br-Ar-C), 103.25 (C=C), 60.19 (OCH_2_), 52.12 (CH-NH), 42.07 (N-CH_2_), 31.51 (CH_2_), 29.84 (CH_2_), 26.35 (CH_2_), 22.60 (CH_2_), 16.17 (CH_3_),14.51 (CH_3_). HRMS (ESI): *m*/*z* calcd for C_20_H_27_BrN_2_O_3_ [M + H]^+^ 423.1283, found 423.1270.

*Ethyl 4-(4-bromophenyl)-1-(3-cyanopropyl)-6-methyl-2-oxo-1,2,3,4-tetrahydropyrimidine-5-carboxylate* (**1i**). White solid, m.p.: 128.8–129.7 °C, yield: 27.2%, *R*f value: 0.4 (CH_2_Cl_2_: CH_3_OH=20:1). ^1^H NMR (400 MHz, CDCl_3_) δ 7.42 (dd, *J* = 8.4, 1.9 Hz, 2H, 2 × Ar-H), 7.09 (dd, *J* = 8.3, 1.4 Hz, 2H, 2 × Ar-H), 5.33 (s, 1H, Ar-CH), 4.14 – 4.03 (m, 2H, OCH_2_), 3.95–3.84 (m, 1H, NCH_2_), 3.74 (dd, *J* = 9.0, 5.4 Hz, 1H, NCH_2)_, 2.52 (s, 3H, =CCH_3_), 2.38 – 2.18 (m, 2H, CH_2_), 1.96 (s, 1H, CH_2_), 1.84 (s, 1H, CH_2_), 1.16 (dd, *J* = 7.5, 6.8 Hz, 3H, CH_3_). ^13^C NMR (101 MHz, DMSO-*d_6_*) δ 166.04 (C=O), 153.20 (C=O), 150.69 (Ar-C), 143.84 (C-N), 131.78 (2 × Ar-C), 128.79 (2 × Ar-C), 120.91 (Br-Ar-C, C≡N), 103.24 (C=C), 60.19 (OCH_2_), 52.11 (CH-NH), 42.07 (N-CH_2_), 22.60 (CH_3_), 16.17 (CH_2_), 14.59 (CH_2_), 14.43 (CH_3_). HRMS (ESI): *m*/*z* calcd for C_18_H_20_BrN_3_O_3_ [M + Na]^+^ 428.0586, found 428.0577.

*Ethyl 4-(4-bromophenyl)-6-methyl-1-(4-nitrobenzyl)-2-oxo-1,2,3,4-tetrahydropyrimidine-5-carboxylate* (**1j**). White solid, m.p.: 183.3–184.6 °C, yield: 9.4%, *R*f value: 0.6 (CH_2_Cl_2_: CH_3_OH=20:1). ^1^H NMR (400 MHz, CDCl_3_) δ 8.17 (d, *J* = 8.7 Hz, 2H, 2 × Ar-H), 7.47–7.44 (m, 2H, 2 × Ar-H), 7.29 (s, 2H, 2 × Ar-H), 7.17–7.10 (m, 2H, 2 × Ar-H), 5.69 (d, *J* = 3.0 Hz, 1H, NH), 5.45 (d, *J* = 3.1 Hz, 1H, Ar-CH), 5.10 (q, *J* = 17.1 Hz, 2H, CH_2_), 4.11 (q, *J* = 7.1 Hz, 2H, OCH_2_), 2.40 (s, 3H, =CCH_3_), 1.18 (t, *J* = 7.1 Hz, 3H, CH_3_). ^13^C NMR (101 MHz, DMSO-*d_6_*) δ 165.88 (C=O), 153.21 (C=O), 149.95 (NO_2_-Ar-C), 147.20 (Ar-C), 147.00 (C-N), 143.70 (Ar-C), 131.96 (2 × Ar-C), 129.02 (2 × Ar-C), 127.77 (2 × Ar-C), 124.25 (2 × Ar-C), 121.12 (Br-Ar-C), 103.87 (C=C), 60.39 (OCH_2_), 52.59 (CH-NH), 45.51 (N-CH_2_), 16.56 (CH_3_), 14.52 (CH_3_). HRMS (ESI): *m*/*z* calcd for C_21_H_20_BrN_3_O_5_ [M + H]^+^ 474.0665, found 474.0658.

#### 3.2.2. N^1^-Substituted Ethyl 4-(4-methoxyphenyl)-6-methyl-2-oxo-1,2,3,4-tetrahydropyrimidine-5-carboxylate **2a**

*Ethyl 1-(4-methoxy-4-oxobutyl)-4-(4-methoxyphenyl)-6-methyl-2-oxo-1,2,3,4-tetrahydropyrimidine-5-carboxylate* (**2a**). White solid, m.p.: 87.8–90.4 °C, yield: 21.7%, *R*f value: 0.4 (CH_2_Cl_2_: CH_3_OH=20:1). ^1^H NMR (400 MHz, CDCl_3_) δ 7.15 (d, *J* = 8.6 Hz, 2H, 2 × Ar-H), 6.80 (d, *J* = 8.6 Hz, 2H, 2 × Ar-H), 5.77 (s, 1H, NH), 5.30 (d, *J* = 1.9 Hz, 1H, Ar-CH), 4.14–4.03 (m, 2H, OCH_2_), 3.96–3.84 (m, 1H, NCH_2_), 3.77 (d, *J* = 5.7 Hz, 3H, OCH_3_), 3.67 (s, 3H, OCH_3_), 3.63 (dd, *J* = 9.0, 4.6 Hz, 1H, NCH_2_), 2.54 (s, 3H, =CCH_3_), 2.29 (td, *J* = 7.0, 2.8 Hz, 2H, COCH_2_), 1.98–1.87 (m, 1H, CH_2_), 1.86–1.76 (m, 1H, CH_2_),1.20–1.13 (m, 3H, CH_3_). ^13^C NMR (101 MHz, DMSO-*d_6_*) δ 173.38 (C=O), 166.18 (C=O), 159.01 (Ar-C), 153.29 (C-N), 149.61 (Ar-C), 136.54 (Ar-C), 127.75 (Ar-C), 127.71 (Ar-C), 114.24 (Ar-C), 104.21 (C=C), 60.11 (OCH_2_), 55.57 (OCH_3_), 52.27 (OCH_3_), 51.82 (CH-NH), 41.37 (N-CH_2_), 30.72 (CH_2_), 25.05 (CH_2_), 16.06 (CH_3_), 14.58 (CH_3_). HRMS (ESI): *m*/*z* calcd for C_20_H_26_BrN_2_O_6_ [M + H]^+^ 391.1869, found 391.1869.

#### 3.2.3. N^1^-Substituted Ethyl 4-([1,1′-biphenyl]-4-yl)-6-methyl-2-oxo-1,2,3,4-tetrahydropyrimidine-5-carboxylates **3a**, **3c**, **3d**, **3e**, **3g**, **3h**

*Ethyl 4-([1,1′-biphenyl]-4-yl)-1-(4-methoxy-4-oxobutyl)-6-methyl-2-oxo-1,2,3,4-tetrahydropyrimidine-5-carboxylate* (**3a**). Yellow solid, m.p.: 141.5–142.7 °C, yield: 33.5%, *R*f value: 0.5 (CH_2_Cl_2_: CH_3_OH=20:1). ^1^H NMR (400 MHz, CDCl_3_) δ 7.55 (dd, *J* = 10.1, 8.0 Hz, 4H, 4 × Ar-H), 7.44 (t, *J* = 7.5 Hz, 2H, 2 × Ar-H), 7.35 (dd, *J* = 11.1, 7.8 Hz, 3H, 3 × Ar-H), 5.87 (d, *J* = 2.8 Hz, 1H, NH), 5.44 (d, *J* = 2.9 Hz, 1H, Ar-CH), 4.21–4.09 (m, 2H, OCH_2_), 4.04–3.90 (m, 1H, NCH_2_), 3.76–3.68 (m, 1H, NCH_2_), 3.68–3.65 (m, 3H, OCH_3_), 2.63–2.54 (m, 3H, =CCH_3_), 2.38–2.27 (m, 2H, OCH_2_), 2.04–1.78 (m, 2H, CH_2_), 1.23 (dd, *J* = 13.0, 5.9 Hz, 3H, CH_3_). ^13^C NMR (151 MHz, DMSO-*d_6_*) δ 173.29 (C=O), 166.08 (C=O), 153.25 (Ar-C), 150.06 (Ar-C), 143.48 (Ar-C), 140.20 (Ar-C), 139.68 (Ar-C), 129.37 (2 × Ar-C), 127.89 (2 × Ar-C), 127.21 (2 × Ar-C), 127.06 (2 × Ar-C), 103.72 (C=C), 60.16 (OCH_2_), 52.46 (OCH_3_), 51.74 (CH-NH), 41.41 (N-CH_2_), 30.67 (CH_2_), 24.99 (CH_2_), 16.07 (CH_3_), 14.56 (CH_3_). HRMS (ESI): *m*/*z* calcd for C_25_H_28_N_2_O_5_ [M + Na]^+^ 459.1896, found 459.1903. 

*Ethyl 4-([1,1′-biphenyl]-4-yl)-1-(2-chlorobenzyl)-6-methyl-2-oxo-1,2,3,4-tetrahydropyrimidine-5-carboxylate* (**3c**). White solid, m.p.: 189.8–190.9 °C, yield: 25.8%, *R*f value: 0.5 (CH_2_Cl_2_: CH_3_OH=20:1). ^1^H NMR (400 MHz, CDCl_3_) δ 7.57 (dd, *J* = 12.2, 4.8 Hz, 4H, 4 × Ar-H), 7.45 (t, *J* = 7.5 Hz, 2H, 2 × Ar-H), 7.41–7.33 (m, 4H, 4 × Ar-H), 7.15 (dtd, *J* = 13.6, 7.4, 6.0 Hz, 2H, 2 × Ar-H), 6.97 (d, *J* = 7.4 Hz, 1H, Ar-H), 5.77 (d, *J* = 3.0 Hz, 1H, NH), 5.55 (d, *J* = 3.0 Hz, 1H, Ar-CH), 5.11 (d, *J* = 2.5 Hz, 2H, NCH_2_), 4.14 (q, *J* = 7.1 Hz, 2H, OCH_2_), 2.40 (s, 3H, =CCH_3_), 1.21 (t, *J* = 7.1 Hz, 3H, CH_3_). ^13^C NMR (151 MHz, DMSO-*d_6_*) δ 166.03 (C=O), 153.25 (C=O), 149.75 (Ar-C), 143.39 (Ar-C), 140.25 (C-N), 139.88 (Ar-C), 135.98 (Ar-C), 131.35 (Ar-C), 129.83 (2 × Ar-C), 129.42 (2 × Ar-C), 129.03 (2 × Ar-C), 127.93 (2 × Ar-C), 127.78 (2 × Ar-C), 127.30 (2 × Ar-C), 127.13 (Ar-C), 104.07 (C=C), 60.33 (OCH_2_), 52.60 (CH-NH), 43.80 (N-CH_2_), 16.12 (CH_3_), 14.53 (CH_3_). HRMS (ESI): *m*/*z* calcd for C_27_H_25_ClN_2_O_3_ [M + H]^+^ 461.1632, found 461.1631. 

*Ethyl 4-([1,1′-biphenyl]-4-yl)-1-(4-bromobenzyl)-6-methyl-2-oxo-1,2,3,4-tetrahydropyrimidine-5-carboxylate* (**3d**). White solid, m.p.: 175.3–177.8 °C, yield: 13.3%, *R*f value: 0.5 (CH_2_Cl_2_: CH_3_OH=20:1). ^1^H NMR (400 MHz, CDCl_3_) δ 7.59 (dd, *J* = 5.2, 3.3 Hz, 2H, 2 × Ar-H), 7.56–7.52 (m, 2H, 2 × Ar-H), 7.47 (dd, *J* = 10.2, 4.7 Hz, 2H, 2 × Ar-H), 7.41 (dd, *J* = 7.7, 4.9 Hz, 2H, 2 × Ar-H), 7.38 (s, 1H, Ar-H), 7.33 (d, *J* = 8.2 Hz, 2H, 2 × Ar-H), 7.04 (d, *J* = 8.4 Hz, 2H, 2 × Ar-H), 5.79 (d, *J* = 3.0 Hz, 1H, NH), 5.52 (d, *J* = 3.1 Hz, 1H, Ar-CH), 5.14 (d, *J* = 15.5 Hz, 1H, NCH_2_), 4.88 (d, *J* = 16.1 Hz, 1H, NCH_2_), 4.14 (q, *J* = 7.1 Hz, 2H, OCH_2_), 2.46 (s, 3H, =CCH_3_), 1.22 (t, *J* = 7.1 Hz, 3H, CH_3_). ^13^C NMR (151MHz, DMSO-*d_6_*) δ 166.00 (C=O), 153.46 (C=O), 149.81 (Ar-C), 143.42 (C-N), 140.24 (Ar-C), 139.83 (Ar-C), 138.64 (Ar-C), 131.92 (2 × Ar-C), 131.82 (2 × Ar-C), 129.40 (2 × Ar-C),128.94 (2 × Ar-C), 127.92 (2 × Ar-C), 127.24 (Ar-C), 127.12 (2 × Ar-C), 120.34 (Br-Ar-C), 104.13 (C=C), 60.27 (OCH_2_), 52.59 (CH-NH), 44.97 (N-CH_2_), 16.50 (CH_3_), 14.52 (CH_3_). HRMS (ESI): *m*/*z* calcd for C_27_H_25_BrN_2_O_3_ [M + Na]^+^ 527.0946, found 527.0945.

*Ethyl 4-([1,1′-biphenyl]-4-yl)-1-(4-methoxybenzyl)-6-methyl-2-oxo-1,2,3,4-tetrahydropyrimidine-5-carboxylate* (**3e**). White solid, m.p.: 163.5–165.8 °C, yield: 24.8%, *R*f value: 0.5 (CH_2_Cl_2_: CH_3_OH=20:1). ^1^H NMR (400 MHz, CDCl_3_) δ 7.56 (d, *J* = 7.2 Hz, 2H, 2 × Ar-H), 7.50 (d, *J* = 8.1 Hz, 2H, 2 × Ar-H), 7.44 (t, *J* = 7.5 Hz, 2H, 2 × Ar-H), 7.36 (d, *J* = 7.3 Hz, 1H, Ar-H), 7.32 (t, *J* = 6.4 Hz, 2H, 2 × Ar-H), 7.08 (d, *J* = 8.5 Hz, 2H, 2 × Ar-H), 6.81 (d, *J* = 8.6 Hz, 2H, 2 × Ar-H), 5.71 (s, 1H, NH), 5.48 (s, 1H, Ar-CH), 5.17 (d, *J* = 15.8 Hz, 1H, NCH_2_), 4.82 (d, *J* = 16.2 Hz, 1H, NCH_2_), 4.11 (q, *J* = 7.1 Hz, 2H, OCH_2_), 3.75 (d, *J* = 3.9 Hz, 3H, CH_3_), 2.46 (d, *J* = 14.7 Hz, 3H, OCH_3_), 1.19 (t, *J* = 7.1 Hz, 3H, CH_3_). ^13^C NMR (151 MHz, DMSO-*d_6_*) δ 166.06 (C=O), 158.59 (C=O), 153.60 (Ar-C), 150.08 (Ar-C), 143.52 (C-N), 140.26 (Ar-C), 139.76 (Ar-C), 130.92 (2 × Ar-C), 129.40 (2 × Ar-C), 128.10 (2 × Ar-C), 127.91 (2 × Ar-C), 127.35 (2 × Ar-C), 126.96 (2 × Ar-C), 114.32 (2 × Ar-C), 104.05 (C=C), 60.21 (OCH_2_), 55.40 (OCH_3_), 52.59 (CH-NH), 44.75 (N-CH_2_), 16.51 (CH_3_), 14.52 (CH_3_). HRMS (ESI): *m*/*z* calcd for C_28_H_28_N_2_O_4_ [M + Na]^+^ 457.2127, found 457.2127.

*Ethyl 4-([1,1′-biphenyl]-4-yl)-1-butyl-6-methyl-2-oxo-1,2,3,4-tetrahydropyrimidine-5-carboxylate* (**3g**). White solid, m.p.: 161.2–165.8 °C, yield: 19.9%, *R*f value: 0.5 (CH_2_Cl_2_: CH_3_OH=20:1). ^1^H NMR (400 MHz, CDCl_3_) δ 7.58–7.50 (m, 4H, 4 × Ar-H), 7.43 (t, *J* = 7.6 Hz, 2H, 2 × Ar-H), 7.37–7.30 (m, 3H, 3 × Ar-H), 5.42 (s, 2H, NH, Ar-CH), 4.13 (q, *J* = 7.1 Hz, 2H, OCH_2_), 3.98–3.87 (m, 1H, NCH_2_), 3.62 (ddd, *J* = 14.8, 14.3, 9.5 Hz, 1H, NCH_2_), 2.55 (s, 3H, =CCH_3_), 1.64–1.50 (m, 2H, CH_2_), 1.31 (dt, *J* = 15.1, 7.5 Hz, 2H, CH_2_), 1.21 (t, *J* = 7.1 Hz, 3H, CH_3_), 0.92 (t, *J* = 7.3 Hz, 3H, CH_3_). ^13^C NMR (151 MHz, DMSO-*d*_6_) δ 166.12 (C=O), 153.25 (C=O), 150.18 (Ar-C), 143.64 (C-N), 140.24 (Ar-C), 139.67 (Ar-C), 129.38 (2 × Ar-C), 127.87 (2 × Ar-C), 127.18 (2 × Ar-C), 127.09 (2 × Ar-C), 127.08 (Ar-C), 103.52 (C=C), 60.12 (OCH_2_), 52.49 (CH-NH), 41.87 (N-CH_2_), 31.91 (CH_2_), 19.88 (CH_2_), 16.15 (CH_3_), 14.56 (CH_3_), 14.10 (CH_3_). HRMS (ESI): *m*/*z* calcd for C_24_H_28_N_2_O_3_ [M + H]^+^ 393.2168, found 393.2178.

*Ethyl 4-([1,1′-biphenyl]-4-yl)-1-hexyl-6-methyl-2-oxo-1,2,3,4-tetrahydropyrimidine-5-carboxylate* (**3h**). White solid, m.p.: 114.4–117.4 °C, yield: 50.5%, *R*f value: 0.4 (CH_2_Cl_2_: CH_3_OH=20:1). ^1^H NMR (400 MHz, CDCl_3_) δ 7.57–7.48 (m, 4H 4 × Ar-H), 7.42 (t, *J* = 7.5 Hz, 2H 2 × Ar-H), 7.33 (t, *J* = 8.0 Hz, 3H, 3 × Ar-H), 5.99 (d, *J* = 2.9 Hz, 1H, NH), 5.42 (d, *J* = 2.9 Hz, 1H, Ar-CH), 4.13 (q, *J* = 7.1 Hz, 2H, OCH_2_), 3.99–3.87 (m, 1H, NCH_2_), 3.57 (ddd, *J* = 14.6, 9.5, 5.4 Hz, 1H, NCH_2_), 2.54 (s, 3H, =CCH_3_), 1.68–1.44 (m, 2H, CH_2_), 1.25 (s, 6H, 3 × CH_2_), 1.21 (t, *J* = 7.1 Hz, 3H, CH_3_), 0.85 (t, *J* = 6.5 Hz, 3H, CH_3_). ^13^C NMR (151 MHz, DMSO-*d*_6_) δ 166.13 (C=O), 153.31 (C=O), 150.26 (Ar-C), 143.58 (C-N), 140.22 (Ar-C), 139.64 (Ar-C), 129.35 (2 × Ar-C), 127.86 (2 × Ar-C), 127.13 (3 × Ar-C), 127.04 (2 × Ar-C), 103.56 (C=C), 60.12 (OCH_2_), 52.34 (CH-NH), 42.04 (N-CH_2_), 31.48 (CH_2_), 29.83 (CH_2_), 26.3 (CH_2_), 22.54 (CH_2_), 16.15 (CH_3_), 14.57 (CH_3_), 14.33 (CH_3_). HRMS (ESI): *m*/*z* calcd for C_26_H_32_N_2_O_3_ [M + H]^+^ 421.2491, found 421.2488.

#### 3.2.4. N^1^-Substituted Ethyl 6-methyl-4-(4-morpholinophenyl)-2-oxo-1,2,3,4-tetrahydropyrimidine-5-carboxylate **4a**

*1-(4-methoxy-4-oxobutyl)-6-methyl-4-(4-morpholinophenyl)-2-oxo-1,2,3,4-tetrahydropyrimidine-5-carboxylate* (**4a**). Orange solid, m.p.: 123.1–125.4 °C, yield: 20.3%, *R*f value: 0.7 (CH_2_Cl_2_: CH_3_OH=10:1). ^1^H NMR (500 MHz, CDCl_3_) δ 7.14 (d, *J* = 8.5 Hz, 2H, 2 × Ar-H), 6.83 (d, *J* = 8.0 Hz, 2H, 2 × Ar-H), 5.49 (s, 1H, NH), 5.29 (s, 1H, Ar-CH), 4.14–4.05 (m, 2H, OCH_2_), 3.95–3.86 (m, 1H, NCH_2_), 3.87–3.82 (m, 4H, 2 × OCH_2_), 3.68 (s, 3H, OCH_3_), 3.65 (dd, *J* = 8.9, 5.9 Hz, 1H, NCH_2_), 3.16–3.09 (m, 4H, 2 × NCH_2_), 2.55 (d, *J* = 16.9 Hz, 3H, =CCH_3_), 2.38–2.25 (m, 2H, CH_2_), 1.99–1.89 (m, 1H, CH_2_), 1.84 (qd, *J* = 13.3, 6.9 Hz, 1H, CH_2_), 1.22 – 1.14 (m, 3H, CH_3_). ^13^C NMR (151 MHz, DMSO-*d_6_*) δ 173.32 (C=O), 166.16 (C=O), 153.27 (Ar-C), 150.77 (C-N), 149.33 (Ar-C), 135.01 (Ar-C), 127.15 (2 × Ar-C), 115.33 (Ar-C), 104.18 (C=C), 66.52 (2 × OCH_2_), 60.02 (OCH_3_), 52.21 (N-CH_2_), 51.79 (N-CH_2_), 48.87 (N-CH_2_), 41.32 (CH-NH), 30.69 (CH_2_), 25.01 (CH_2_), 16.00 (CH_3_), 14.55 (CH_3_). HRMS (ESI): *m*/*z* calcd for C_23_H_31_N_3_O_6_ [M + Na]^+^ 468.2111, found 468.2105.

#### 3.2.5. N^1^-Substituted Ethyl 6-methyl-4-(4-nitrophenyl)-2-oxo-1,2,3,4-tetrahydropyrimidine-5-carboxylate **5a**

*Ethyl 1-(4-methoxy-4-oxobutyl)-6-methyl-4-(4-nitrophenyl)-2-oxo-1,2,3,4-tetrahydropyrimidine-5-carboxylate* (**5a**). Yellow solid, m.p.: 108.4–111.2 °C, yield: 17.8%, *R*f value: 0.7 (CH_2_Cl_2_: CH_3_OH=10:1). ^1^H NMR (400 MHz, CDCl_3_) δ 8.16 (d, *J* = 8.7 Hz, 2H, 2 × Ar-H), 7.42 (d, *J* = 8.7 Hz, 2H, 2 × Ar-H), 5.66 (s, 1H, NH), 5.47 (d, *J* = 2.4 Hz, 1H, Ar-CH), 4.11 (q, *J* = 7.1 Hz, 2H, OCH_2_), 3.96–3.83 (m, 1H, NCH_2_), 3.73–3.60 (m, 4H, NCH_2_, OCH_3_), 2.56 (s, 3H, =CCH_3_), 2.32–2.23 (m, 2H, OCH_2_), 1.98–1.74 (m, 2H, CH_2_), 1.18 (t, *J* = 7.1 Hz, 3H, CH_3_). ^13^C NMR (151 MHz, DMSO-*d_6_*) δ 173.24 (C=O), 165.77 (C=O), 152.93 (Ar-C), 151.64 (C-N), 151.16 (Ar-C), 147.21 (Ar-C), 127.89 (2 × Ar-C), 124.28 (Ar-C), 102.758 (C=C), 60.30 (OCH_2_), 52.37 (OCH_3_), 51.75 (N-CH_2_), 41.47 (CH-NH), 30.62 (CH_2_), 16.08 (CH_3_), 14.50 (CH_3_) HRMS (ESI): *m*/*z* calcd for C_19_H_23_N_3_O_7_ [M + H]^+^ 406.1614, found 406. 1611.

#### 3.2.6. N^1^-Substituted Ethyl 6-methyl-2-oxo-4-(pyridin-4-yl)-1,2,3,4-tetrahydropyrimidine-5-carboxylate **6a**

*Ethyl 1-(4-methoxy-4-oxobutyl)-6-methyl-2-oxo-4-(pyridin-4-yl)-1,2,3,4-tetrahydropyrimidine-5-carboxylate* (**6a**). Yellow solid, m.p.: 97.8–100.3 °C, yield: 21.7%, *R*f value: 0.6 (CH_2_Cl_2_: CH_3_OH=10:1). ^1^H NMR (400 MHz, CDCl_3_) δ 8.54 (d, *J* = 5.8 Hz, 2H, 2 × Pyridine-H), 7.20 (d, *J* = 6.0 Hz, 2H, 2 × Pyridine-H), 6.00 (d, *J* = 3.4 Hz, 1H, NH), 5.39 (d, *J* = 3.5 Hz, 1H, Ar-CH), 4.15 (q, *J* = 7.1 Hz, 2H, OCH_2_), 3.90 (ddd, *J* = 15.1, 9.7, 5.8 Hz, 1H, NCH_2_), 3.67 (s, 3H, OCH_3_), 3.66–3.60 (m, 1H, NCH_2_), 2.56 (s, 3H, =CCH_3_), 2.29 (td, *J* = 7.0, 3.9 Hz, 2H, CH_2_), 1.79 (ddt, *J* = 13.3, 9.7, 6.6 Hz, 2H, CH_2_), 1.21 (t, *J* = 7.1 Hz, 3H, CH_3_). ^13^C NMR (101 MHz, DMSO-*d_6_*) δ 173.14 (C=O), 165.73 (C=O), 153.04 (2 × Pyridine-C), 152.474 (C=O), 151.02 (2 × Pyridine-C), 150.27 (C-N), 121.45 (2 × Pyridine-C), 102.45 (C=C), 60.19 (CH_2_), 51.75 (Ar-CH), 41.40 (CH_2_), 30.57 (CH_2_), 24.83 (CH_2_ 2), 15.97 (CH_3_), 14.41 (CH_3_). HRMS (ESI): *m*/*z* calcd for C_18_H_23_N_3_O_5_ [M + H]^+^ 362.1716, found 362.1704.

#### 3.2.7. N^1^-Substituted 4-(4-bromophenyl)-6-methyl-2-oxo-1,2,3,4-tetrahydropyrimidine-5-carboxamide **7c**, **7d**, **7e**, **7f**

*4-(4-bromophenyl)-1-(2-chlorobenzyl)-6-methyl-2-oxo-1,2,3,4-tetrahydropyrimidine-5-carboxamide* (**7c**). White solid, m.p.: 186.4–188.5 °C, yield: 14.6%, *R*f value: 0.5 (CH_2_Cl_2_: CH_3_OH=10:1). ^1^H NMR (400 MHz, CDCl_3_) δ 7.51 (d, *J* = 8.4 Hz, 2H, 2 × Ar-H), 7.38 (dd, *J* = 5.4, 3.8 Hz, 1H, Ar-H), 7.24 (dd, *J* = 8.9, 6.7 Hz, 4H, 4 × Ar-H), 7.08 – 7.04 (m, 1H, Ar-H), 5.83 (s, 1H, NH), 5.36 (s, 1H, Ar-CH), 5.05 (s, 2H, NCH_2_), 2.19 (s, 3H, =CCH_3_). ^13^C NMR (101 MHz, DMSO-*d_6_*) δ 169.09 (C=O), 153.52 (C=O), 143.36 (Ar-C), 138.64 (Ar-C), 136.65 (C-N), 131.83 (Ar-C-Cl), 131.39 (2 × Ar-C), 129.83 (2 × Ar-C), 129.28 (Ar-C), 129.00 (Ar-C), 127.82 (Ar-C), 127.30 (Ar-C), 121.10 (Br-Ar-C), 109.76 (C=C), 53.96 (CH), 43.34 (CH_2_), 16.36 (CH_3_). HRMS (ESI): *m*/*z* calcd for C_19_H_17_BrClN_3_O_2_ [M − H]^−^ 432.0114, found 432.0110.

*1-(4-bromobenzyl)-4-(4-bromophenyl)-6-methyl-2-oxo-1,2,3,4-tetrahydropyrimidine-5-carboxamide* (**7d**). White solid, m.p.: 280.3–280.7 °C, yield: 58.2%, *R*f value: 0.7 (CH_2_Cl_2_: CH_3_OH=10:1). ^1^H NMR (400 MHz, DMSO) δ 7.56–7.43 (m, 4H, 4 × Ar-H), 7.15 (s, 2H, 2 × Ar-H), 7.03 (d, *J* = 7.1 Hz, 2H, 2 × Ar-H), 5.17 (s, 1H, Ar-CH), 4.92 (d, *J* = 17.6 Hz, 1H, NCH_2_), 4.67 (t, *J* = 14.5 Hz, 1H, NCH_2_), 2.02 (s, 3H, =CCH_3_). ^13^C NMR (101 MHz, DMSO-*d_6_*) δ 169.06 (C=O), 153.73 (C=O), 143.42 (Ar-C), 139.33 (C-N), 138.70 (Ar-C), 131.80 (4 × Ar-C), 129.19 (2 × Ar-C), 129.07 (2 × Ar-C), 121.03 (Br-Ar-C), 120.29 (Br-Ar-C), 109.88 (C=C), 53.91 (CH), 44.61 (CH_2_), 16.75 (CH_3_). HRMS (ESI): *m*/*z* calcd for C_19_H_17_Br_2_N_3_O_2_ [M + H]^+^ 477.9766, found 477.9769.

*4-(4-bromophenyl)-1-(4-methoxybenzyl)-6-methyl-2-oxo-1,2,3,4-tetrahydropyrimidine-5-carboxamide* (**7e**). White solid, m.p.: 256.4–257.9 °C, yield: 73.8%, *R*f value: 0.6 (CH_2_Cl_2_: CH_3_OH=10:1). ^1^H NMR (400 MHz, DMSO-*d*_6_) δ 7.80 (d, *J* = 3.0 Hz, 1H, NH_2_), 7.48 (d, *J* = 8.4 Hz, 2H, 2 × Ar-H), 7.34 (s, 1H, NH_2_), 7.14 (d, *J* = 8.4 Hz, 2H, 2 × Ar-H), 6.99 (d, *J* = 8.6 Hz, 3H, 2 × Ar-H, NH), 6.81 (d, *J* = 8.7 Hz, 2H, 2 × Ar-H), 5.16 (d, *J* = 2.4 Hz, 1H, Ar-CH), 4.92 (d, *J* = 16.5 Hz, 1H, NCH_2_), 4.58 (d, *J* = 16.5 Hz, 1H, NCH_2_), 3.69 (s, 3H, OCH_3_), 2.04 (s, 3H, =CCH_3_). ^13^C NMR (101 MHz, DMSO-*d_6_*) δ 168.97 (C=O), 158.48 (Ar-C-O), 153.71 (C=O), 143.42 (Ar-C), 138.98 (C-N), 131.59 (2 × Ar-C), 131.52 (2 × Ar-C), 129.08 (2 × Ar-C), 128.01 (Ar-C), 120.81 (Br-Ar-C), 114.19 (2 × Ar-C), 109.58 (C=C), 55.44 (CH_3_), 53.73 (CH), 44.29 (CH_2_), 16.59 (CH_3_). HRMS (ESI): *m*/*z* calcd for C_20_H_20_BrN_3_O_3_ [M + Na]^+^ 452.0586, found 452.0571.

*4-(4-bromophenyl)-6-methyl-2-oxo-1-propyl-1,2,3,4-tetrahydropyrimidine-5-carboxamide* (**7f**). White solid, m.p.: 238.4–239.5 °C, yield: 21.7%, *R*f value: 0.5 (CH_2_Cl_2_: CH_3_OH=10:1). 1H NMR (400 MHz, DMSO) δ 7.60 (d, J = 3.0 Hz, 1H, Ar-H), 7.49 (d, J = 8.4 Hz, 2H, 2 × Ar-H), 7.15 (t, J = 7.8 Hz, 2H, 2 × Ar-H), 5.09 (d, J = 2.6 Hz, 1H, Ar-CH), 3.73–3.61 (m, 1H, NCH_2_), 2.47 (d, J = 1.6 Hz, 3H, =CCH_3_), 2.13 (s, 3H, NCH_2_, CH_2_), 0.72 (t, J = 7.4 Hz, 3H, CH_3_). ^13^C NMR (101 MHz, DMSO-*d_6_*) δ 169.36 (C=O), 153.59 (C=O), 143.56 (Ar-C), 138.96 (C-N), 131.74 (2 × Ar-C), 129.06 (2 × Ar-C), 120.88 (Br-Ar-C), 109.67 (C=C), 53.71 (CH), 43.37 (CH_2_), 23.24 (CH_2_), 16.60 (CH_3_), 11.46 (CH_3_). HRMS (ESI): *m*/*z* calcd for C_15_H_18_BrN_3_O_2_ [M + Cl]^−^ 386.0271, found 386.0269.

#### 3.2.8. N^1^-Substituted 4-(4-bromophenyl)-6-methyl-2-oxo-N-phenyl-1,2,3,4-hydropyrimidine-5-carboxamides **8a**, **8d**, **8e**

*Methyl 4-(4-(4-bromophenyl)-6-methyl-2-oxo-5-(phenylcarbamoyl)-3,4-dihydropyrimidin-1(2H)-yl)butanoate* (**8a**). White solid, m.p.: 182.4–185.0 °C, yield: 25.2%, *R*f value: 0.5 (CH_2_Cl_2_: CH_3_OH=10:1). ^1^H NMR (400 MHz, DMSO) δ 9.89 (s, 1H, NH_2_), 7.77 (d, *J* = 3.0 Hz, 1H, NH_2_), 7.54 (dd, *J* = 15.5, 8.1 Hz, 4H, 4 × Ar-H), 7.27 (t, *J* = 7.9 Hz, 2H, 2 × Ar-H), 7.22 (d, *J* = 8.4 Hz, 2H, 2 × Ar-H), 7.03 (t, *J* = 7.4 Hz, 1H, Ar-H), 5.22 (s, 1H, Ar-CH), 3.86–3.76 (m, 1H, NCH_2_), 3.59 (d, *J* = 4.8 Hz, 3H, CH_3_), 3.43 (ddd, *J* = 14.4, 8.9, 5.6 Hz, 1H, NCH_2_), 2.24 (d, *J* = 7.4 Hz, 2H, CH_2_), 2.15 (s, 3H, =CCH_3_), 1.80 (dd, *J* = 13.1, 5.2 Hz, 1H, COCH_2_), 1.70–1.60 (m, 1H, COCH_2_). ^13^C NMR (101 MHz, DMSO-*d_6_*) δ 173.33 (C=O), 166.18 (C=O), 153.36 (C=O), 143.19 (Ar-C), 139.38 (C-N), 138.66 (Ar-C-NH), 131.75 (2 × Ar-C), 129.02 (2 × Ar-C), 128.82 (2 × Ar-C), 123.80 (Ar-C), 120.91 (2 × Ar-C), 120.04 (Br-Ar-C), 110.23 (C=C), 53.86 (CH), 51.74 (CH_3_), 40.95 (CH_2_), 30.71 (CH_2_), 25.16 (CH_2_), 16.66 (CH_3_). HRMS (ESI): *m*/*z* calcd for C_23_H_24_BrN_3_O_4_ [M + H]^+^ 486.1028, found 486.1016.

*1-(4-bromobenzyl)-4-(4-bromophenyl)-6-methyl-2-oxo-N-phenyl-1,2,3,4-tetrahydropyrimidine-5-carboxamide* (**8d**). White solid, m.p.: 171.8–175.2 °C, yield: 16.7%, *R*f value: 0.4 (CH_2_Cl_2_: CH_3_OH=10:1). ^1^H NMR (400 MHz, DMSO-*d*_6_) δ 9.87 (s, 1H, NH_2_), 7.94 (s, 1H, NH_2_), 7.54–7.45 (m, 6H, 6 × Ar-H), 7.26–7.15 (m, 4H, 4 × Ar-H), 7.10 (d, *J* = 8.2 Hz, 2H, 2 × Ar-H), 6.98 (t, *J* = 7.3 Hz, 1H, Ar-H), 5.28 (s, 1H, Ar-CH), 4.93 (d, *J* = 17.0 Hz, 1H, NCH_2_), 4.72 (d, *J* = 11.6 Hz, 1H, NCH_2_), 2.00 (s, 3H, =CCH_3_). ^13^C NMR (100 MHz, DMSO-*d_6_*) δ 165.58 (C=O), 152.83 (C=O), 144.02 (Ar-C), 139.51 (C-N), 139.06 (Ar-C-NH), 131.75 (Ar-C), 128.92 (4 × Ar-C), 123.55 (6 × Ar-C), 120.79 (Ar-C), 120.04 (4 × Ar-C), 105.40 (C=C), 55.00 (CH), 48.99 (CH_2_), 17.44 (CH_3_). HRMS (ESI): *m*/*z* calcd for C_25_H_21_Br_2_N_3_O_2_ [M + H]^+^ 556.0058, found 556.0043.

*4-(4-bromophenyl)-1-(4-methoxybenzyl)-6-methyl-2-oxo-N-phenyl-1,2,3,4-tetrahydropyrimidine-5-carboxamide* (**8e**). White solid, m.p.: 191.7–193.5 °C, yield: 28.7%, *R*f value: 0.5 (CH_2_Cl_2_: CH_3_OH=10:1). ^1^H NMR (400 MHz, DMSO-*d*_6_) δ 9.90 (s, 1H, NH_2_), 7.92 (d, *J* = 3.0 Hz, 1H, NH_2_), 7.53 (d, *J* = 8.4 Hz, 4H, 4 × Ar-H), 7.29–7.20 (m, 4H, 4 × Ar-H), 7.09 (d, *J* = 8.6 Hz, 2H, 4 × Ar-H), 7.03 (d, *J* = 7.4 Hz, 1H, Ar-H), 6.88 (d, *J* = 8.7 Hz, 2H, 2 × Ar-H), 5.31 (s, 1H, Ar-CH), 4.97 (d, *J* = 16.0 Hz, 1H, NCH_2_), 4.69 (d, *J* = 16.2 Hz, 1H, NCH_2_), 3.74 (s, 3H, OCH_3_), 2.06 (s, 3H, =CCH_3_). ^13^C NMR (101 MHz, DMSO-*d_6_*) δ 166.19 (C=O), 158.68 (Ar-C-O), 153.74 (C=O), 143.43 (Ar-C), 139.48 (C-N), 139.05 (Ar-C-NH), 131.86 (2 × Ar-C), 131.61 (2 × Ar-C), 129.15 (2 × Ar-C), 129.10 (2 × Ar-C), 128.21 (Ar-C), 123.95 (Ar-C), 121.09 (2 × Ar-C), 120.28 (Br-Ar-C), 114.39 (2 × Ar-C), 109.96 (C=C), 55.60 (CH_3_), 54.35 (CH), 44.57 (CH_2_), 16.94 (CH_3_). HRMS (ESI): *m*/*z* calcd for C_26_H_24_BrN_3_O_3_ [M + H]^+^ 506.1079, found 506.1069.

### 3.3. N^1^ and N^3^-Substituted Ethyl 4-(4-bromophenyl)-6-methyl-2-oxo-1,2,3,4-tetrahydropyrimidine-5-carboxylates

*Ethyl 4-(4-bromophenyl)-1,3-bis(2-chlorobenzyl)-6-methyl-2-oxo-1,2,3,4-tetrahydropyrimidine-5-carboxylate.* White solid, m.p.: 145.1-146.6 °C, yield: NA. ^1^H NMR (400 MHz, CDCl_3_) δ 7.48-7.41 (m, 2H, 2 × Ar-H), 7.41 -7.33 (m, 3H, 2 × Ar-H), 7.31-7.24 (m, 3H, 2 × Ar-H), 7.24-7.12 (m, 4H, 4 × Ar-H), 5.38 (s, 1H, Ar-CH), 5.29 (d, J = 15.7 Hz, 1H, NCH_2_), 5.12 (s, 2H, NCH_2_), 4.11 (q, J = 7.1 Hz, 2H, OCH_2_), 4.05 (d, J = 15.7 Hz, 1H, NCH_2_), 2.39 (s, 3H, =CCH_3_), 1.19 (t, J = 7.1 Hz, 3H, CH_3_). ^13^C NMR (100 MHz, DMSO-d_6_) δ 165.47 (C=O), 153.08 (C=O), 149.52 (Ar-C), 140.96 (C-N), 135.74 (Ar-C), 134.47 (Ar-C), 133.00(Ar-C), 132.20(Ar-C), 131.56 (2 × Ar-C), 130.12 (2 × Ar-C), 129.95 (Ar-C), 129.87 (Ar-C), 129.78 (Ar-C), 129.41 (Ar-C), 129.22 (Ar-C), 127.95 (Ar-C), 127.83 (Ar-C), 127.43 (Ar-C), 121.70 (Br-Ar-C), 104.15 (C=C), 60.54 (CH), 57.84 (CH_2_), 47.89 (CH_2_), 45.28 (CH_2_), 16.29 (CH_3_), 14.48 (CH_3_). HRMS (ESI): m/z calcd for C_28_H_25_BrCl_2_N_2_O_3_ [M+H]^+^ 587.0504, found 587.0520.

### 3.4. The Information on Reaction Condition Optimization

In order to investigate the effects of reaction conditions on N^1^ selectivity, we designed orthogonal tests table of different aspects to optimize the reaction, including temperature, time, amount of solvent, dosing interval, the presence of a phase transfer catalyst, different mole equivalent of base and halohydrocarbons. Each condition set include two variables of low dose and high dose (Table S2 in Supplementary Materials). Orthogonal test is widely used in everyday studies and research, because it could handle a complex issue with much lower cost and less time. Orthogonal tests *L*_8_(_2_^7^) were applied to analyze the influence of the seven factors above on the selectivity and yield of N1-alkylation of DHPMs.

Results of orthogonal tests for N^1^-alkylation of DHPMs in tetrabutylammonium hydroxide system were shown in Table S2 and statistics analysis wascarried out with the extremely analysis method. The change of all conditions had no effect on N^1^-alkylation selectivity. According to the importance of influence, they were sorted into temperature (R 36.9), dosing interval (R 22.3), reaction time (R 19.5), phase transfer catalyst (R 16.5) and mole ratio of halohydrocarbons (R 11.9). Two other factors, the mole equivalent of base and the amount of solvent, had little effect on yield (R<10). Therefore, the yield could be increased by raising the reaction temperature and prolonging the reaction time. Meanwhile the selectivity was maintained.

In accordance with the requirements of saving raw materials and lowering costs, 1.7 equivalents of tetrabutylammonium hydroxide, and other conditions selected better factors for orthogonal tests were selected. The N^1^-alkylation of DHPMs was synthesized from compound **1**, compound **2**. The optimal conditions were the mole ratio 1:1.8:1.7 (compound **1**, compound **2** and tetrabutylammonium hydroxide), DMF 20 mL/g, TBA was added as a catalyst. After the reaction of compound **1** and TBA in DMF for 1.5 h, compound **2** and potassium iodide(KI) were added to the mixture and stirred for 16 h at 45 °C.

### 3.5. In Vitro Studies

#### 3.5.1. Cell Culture

U87, U251, Hela and A549 cell lines were obtained from Stem Cell Bank, Chinese Academy of Sciences. They were maintained in 75 cm^2^ culture flasks at 37 °C in a humidified air incubator with 5% CO2. The high-glucose Dulbecco´s modified Eagle medium (DMEM) supplemented with 10% fetal bovine serum (FBS; Gemini, New York, NY, USA), and penicillin-streptomycin (Solarbio; Beijing, China) was used to culture cells. For all cell lines, the medium was renewed every 2 days until cells reach approximately 90–95% confluence. Then, they were detached by trypsin (Beyotime, China) and before the experiments, cells were counted using a hemocytometer and suitably diluted in the adequate complete culture medium.

#### 3.5.2. Preparation of Compounds Solutions

BIIB021 was purchased from Aladdin Industrial Co. (Shanghai, China). The remaining compounds were synthesized in the authors laboratory. All compounds were dissolved individually in DMSO in a concentration of 10 mM and stored at −20 °C. From this stock solution, the various working solutions of the compounds in different concentrations were prepared by adequate dilutions in the complete culture medium before each experiment.

#### 3.5.3. MTT Assay

The in vitro antiproliferative effects were evaluated by the 3-(4,5-dimethylthiazol-2-yl)-2,5-diphenyltetrazolium bromide (MTT; Sigma-Aldrich, St Louis, MO, USA) assay [31,32]. After reaching confluence, cells were trypsinized and counted using a hemocytometer. Then, cell suspension/well with density of 4 × 10^4^ cells/mL was seeded in 96-well culture plates and left to adhere for 24 h. After adherence, the medium was replaced by the several solutions of the compounds in study (10 µM for preliminary studies and 1.25, 2.5, 5, 10, 20, and 40 µM for concentration-response studies) in the appropriate culture medium for approximately 72 h. Untreated cells were used as the negative control. Each experiment was performed in triplicate and independently repeated. Then, the medium was removed, 20 µL of the MTT solution (5 mg/mL), prepared in the appropriate serum-free medium, was added to each well, followed by incubation for approximately 3 h at 37 °C. Then, the MTT containing medium was removed and the formazan crystals were dissolved in DMSO. The absorbance was measured at 570 nm using a microplate reader Bio-rad Xmark spectrophotometer (Molecular Devices, Sunnyvale, CA, USA). After background subtraction, cell proliferation values were expressed as percentage relatively to the absorbance determined in negative control cells.

### 3.6. In Vivo Studies on Xenograft Model

C57 mice (6−8 weeks old, male) were used to establish the xenograft tumors following our published Protocol [33,34]. In brief, GL261 cells (5 × 10^6^) were inoculated subcutaneously in right frank regions. The mice were divided into four groups randomly as control, BIIB021 (30 mg/kg), 303 (100 mg/kg), and 305(100 mg/kg) with eight mice per group. The mice were intra-gastric administration once a day for compound **3d** and **3g** starting from the second day, and body weights was measured every 3 days. At the end of treatment, animals were euthanized and the tumors were stripped and weighed after two weeks. All data were expressed as mean ± SD (*n* = 5). * *p* < 0.05, compared with control group. The use of animals was approved by the Animal Experimentation Ethics Committee of Yantai University (protocol number 20180601) in accordance with the guidelines for ethical conduct in the care and use of animals.

### 3.7. Log P Properties

The logarithm of the partition coefficient (Log P) properties of compounds were calculated by ACD/labs 6.00.

### 3.8. Pharmacophore Requirements

The GALAHAD module of Sybyl-X 2.0 (Certara, Princeton, NJ, USA) was used to generate pharmacophore. Thirteen DHPMs derivatives were selected with good activity against anticancer. All the structures are attached in Table S3 [20,35,36]. The final pharmacophore models were achieved with follow operations, including a population size value of 20, a maximum generation value of 100 and the value of molecular required hitting was 8.

## 4. Conclusions

A wide range of organic bases had been selected to study the N^1^ and N3 dialkylation of DHPMs. Selective alkylation of N^1^ was achieved with the use of tetrabutylammonium hydroxide. All the synthesized derivatives were screened for their anti-proliferative activity in U87, U251, Hela and A549 cell lines using the MTT assay. The study demonstrated that these compounds were more selective toward glioma tumor types. Introduction of the aryl or alkyl chain in the R^3^, and low electron-donating group in the R^1^ of DHPMs exhibited potent anti-proliferative activity. The in vivo efficacy study showed that compound **3d** may have the potential to serve as lead compound for novel anti-tumor drugs to treat glioma. The study may provide a foundation for the future development of DHPMs as a new anti-tumor drug.

## Figures and Tables

**Table 1:**
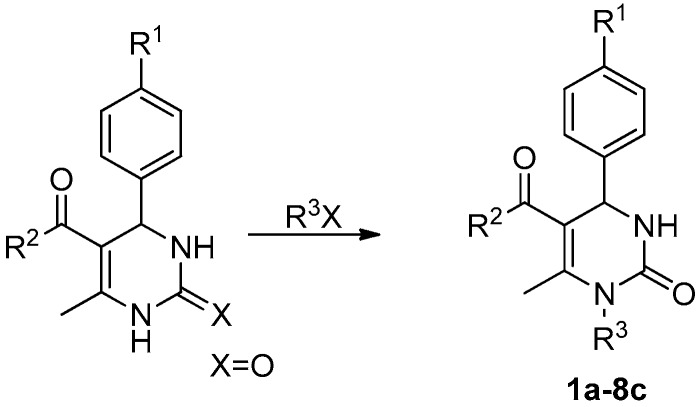
Preparation of N^1^-alkylated DHPMs with different halohydrocarbons.

DHPMs (1a–8e)	R^1^	R^2^	R^3^	N^1^-Alkylation Yield (%) ^a^	Log P (Partition Coefficient)
**1a**	Br	OEt	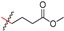	8.0	3.33
**1b**	Br	OEt	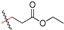	52.2	3.65
**1c**	Br	OEt	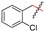	35.1	5.27
**1d**	Br	OEt	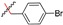	32.3	5.45
**1e**	Br	OEt	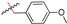	43.6	4.59
**1f**	Br	OEt	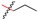	20.0	3.91
**1g**	Br	OEt	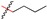	69.7	4.44
**1h**	Br	OEt	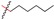	55.8	5.50
**1i**	Br	OEt	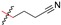	27.2	2.71
**1j**	Br	OEt	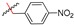	9.4	4.41
**2a**	OCH_3_	OEt	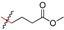	21.7	2.47
**3a**	Ph	OEt	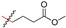	33.5	4.32
**3c**	Ph	OEt	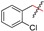	25.8	6.26
**3d**	Ph	OEt	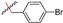	13.3	6.43
**3e**	Ph	OEt	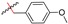	24.8	5.58
**3g**	Ph	OEt	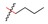	19.9	5.43
**3h**	Ph	OEt	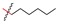	50.5	6.49
**4a**	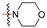	OEt	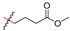	20.3	1.70
**5a**	NO_2_	OEt	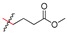	17.8	2.29
**6a**	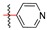	OEt	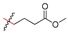	21.7	1.07
**7c**	Br	NH_2_	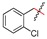	14.6	3.92
**7d**	Br	NH_2_	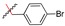	58.2	4.09
**7e**	Br	NH_2_	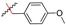	73.8	3.24
**7f**	Br	NH_2_	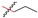	21.7	2.55
**8a**	Br	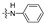	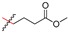	25.2	4.36
**8d**	Br	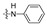	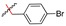	16.7	6.47
**8e**	Br	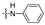	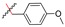	28.7	5.62

^a^ The yields relate to the use of tetrabutylammonium hydroxide as a base.

**Table 2:** Survival rate of all compounds against U87, U251, Hela, A549 cell lines at 72 h.

DHPMs (1a–8e)	Survival Rate of Four Different Cells (%)			
	U87 ^a^	U251 ^a^	Hela ^a^	A549 ^a^
**1a**	87.34 ± 1.24	67.14 ± 4.61	69.81 ± 2.04	76.80 ± 1.76
**1b**	84.93 ± 0.72	78.25 ± 7.88	71.44 ± 0.67	59.48 ± 2.63
**1c**	97.83 ± 4.32	85.20 ± 1.16	71.96 ± 0.96	62.86 ± 0.97
**1d**	50.83 ± 0.25	51.07 ± 2.56	53.71 ± 1.08	73.26 ± 2.69
**1e**	89.41 ± 1.47	71.65 ± 4.64	51.05 ± 1.51	54.10 ± 1.44
**1f**	95.09 ± 12.76	74.75 ± 0.79	62.48 ± 1.41	61.86 ± 1.24
**1g**	84.04 ± 3.08	72.74 ± 9.08	51.05 ± 1.42	50.68 ± 1.22
**1h**	60.05 ± 1.55	56.40 ± 4.21	51.05 ± 0.63	59.28 ± 2.97
**1i**	94.46 ± 5.33	80.10 ± 7.98	62.48 ± 1.55	69.93 ± 0.34
**1j**	60.69 ± 1.89	63.27 ± 3.40	65.72 ± 0.39	66.60 ± 1.35
**2a**	85.54 ± 3.15	72.22 ± 9.37	67.90 ± 1.62	78.92 ± 1.62
**3a**	65.62 ± 3.77	48.23 ± 3.97	59.56 ± 3.87	58.92 ± 2.41
**3c**	100.69 ± 2.75	85.60 ± 4.04	73.81 ± 5.73	51.92 ± 3.35
**3d**	51.98 ± 1.64	49.49 ± 4.73	63.57 ± 2.74	64.94 ± 4.16
**3e**	70.94 ± 5.16	71.28 ± 3.76	57.38 ± 0.40	58.35 ± 4.25
**3g**	54.27 ± 0.88	51.07 ± 4.32	44.85 ± 1.08	43.70 ± 2.38
**3h**	60.37 ± 2.71	51.21 ± 0.58	46.02 ± 0.85	55.16 ± 2.43
**4a**	71.26 ± 2.08	77.28 ± 5.38	62.32 ± 2.26	81.36 ± 1.63
**5a**	75.53 ± 4.89	76.22 ± 5.48	47.96 ± 5.00	52.22 ± 0.19
**6a**	81.20 ± 4.44	82.03 ± 6.38	68.80 ± 5.39	68.04 ± 3.02
**7c**	81.02 ± 2.98	97.29 ± 3.47	74.26 ± 7.29	91.51 ± 1.21
**7d**	78.91 ± 4.81	84.79 ± 5.14	65.73 ± 3.44	74.22 ± 1.96
**7e**	76.22 ± 3.21	83.07 ± 2.22	66.91 ± 2.78	68.17 ± 7.73
**7f**	77.11 ± 6.22	81.09 ± 4.01	78.32 ± 2.47	71.78 ± 4.25
**8a**	81.22 ± 4.56	89.27 ± 3.64	66.91 ± 4.92	72.03 ± 5.56
**8d**	68.21 ± 2.16	71.53 ± 5.85	78.32 ± 2.28	71.29 ± 3.84
**8e**	81.86 ± 0.71	73.90 ± 2.22	79.80 ± 3.79	85.65 ± 2.39

^a^ U87, U251, HeLa, A549 cell lines were exposed at concentrations 10 µM at 72 h.

**Table 3:** Selected compounds studied for half maximal inhibitory concentration (IC50) in U87 and U251 cell lines.

Compound	IC_50_ (µM)	
	U87	U251
**1d**	9.72 ± 0.29	13.91 ± 0.86
**1h**	9.30 ± 0.81	14.01 ± 0.76
**3d**	12.02 ± 0.5	6.36 ± 0.73
**3g**	9.52 ± 0.81	7.32 ± 0.86
**BIIB021 ^a^**	2.07 ± 0.13	0.3 ± 0.043

^a^**BIIB021** as a positive control.

**Table 4:** Inhibitory effects of compounds **3d** and **3g** on the xenograft tumor growth of GL261 in C57 mice.

Groups	Dosage (mg/kg)	Number Initial/End	Body Weight (g)		Tumor Weight (g)	IR (%)
			Initial	End		
**Control**	0	8/8	20.2 ± 0.4	22.0 ± 1.2	0.83 ± 0.24	
**3d**	100	8/8	19.6 ± 0.6	21.5 ± 1.5	0.37 ± 0.19	54.9
**3g**	100	8/8	19.8 ± 0.5	21.8 ± 0.8	0.54 ± 0.25	34.3
**BIIB021**	30	8/8	20.1 ± 0.7	21.3 ± 1.0	0.33 ± 0.17	59.7

## Supplementary Materials

The following are available online. Copies of the ^1^H-NMR and ^13^C-NMR spectra of the compounds and Tables S1, S2 and S3 are available online.
