# Supplementary material for: Synthesis, Characterization, and Anticancer Activities Evaluation of Compounds Derived from 3,4-Dihydropyrimidin-2(1H)-one
Source: Molecules. 2019 Mar 3;24(5):891. doi: 10.3390/molecules24050891 (PMC6429579; doi:10.3390/molecules24050891)
Supplement: Supplementary file 1 [file molecules-24-00891-s001.zip › molecules-433653-suppl/molecules-433653-Supplementary materials-Table S1.pdf]

Supplementary Materials

Synthesis, Characterization, and Anticancer Activities Evaluation of Compounds Derived from 3,4-Dihydropyrimidin-2(1*H*)-one

Ye Liu [1], Jiaqi Liu [1], Renmei Zhang [1], Yan Guo [1], Hongbo Wang [1], Qingguo Meng [1], Yuan Sun [2] and Zongliang Liu [1,*]

[1] School of Pharmacy, Key Laboratory of Molecular Pharmacology and Drug Evaluation (Yantai University), Ministry of Education, Collaborative Innovation Center of Advanced Drug Delivery System and Biotech Drugs in Universities of Shandong, Yantai University, Yantai 264005, China; liuyeer126@126.com (Y.L.); jiaqi.miami@gmail.com (J.L.); zhangrenmeigrace@126.com (R.Z.); 18865672173@163.com (Y.G.); hongbowangyt@163.com (H.W.); qinggmeng@163.com (Q.M.)

[2] Department of Biochemistry and Molecular Medicine, School of Medicine, University of California Davis, Sacramento, CA 95817, USA; ysysun@ucdavis.edu

* Correspondence: lzl_0_0@126.com; Tel.: +86-0535-6706023

Table S1. Results of N[1]-alkylation of DHPMs reaction with different bases.

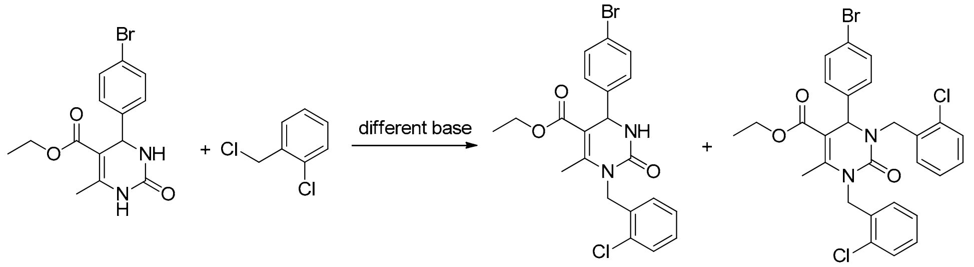

Entry	Base	Additive	Reaction time (h)	Temperature (°C)	N1-alkylation Yield[a] (%)	N1 and N3-dialkylation Yield (%)
1	potassium carbonate	KI TBA	24	r.t.[b]	\	\
2	cesium carbonate	KI TBA	22	r.t.	25	27
3	lithium hydroxide	KI TBA	22	r.t	12	31
4	sodium hydride	\	1.5	0-5 then r.t.	9	18
5	triethylamine	KI TBA	22	r.t.	\	\
6	1,8-diazabicyclo[5.4.0]undec-7-ene	KI TBA	22	r.t.	\	\
7	tetramethylguanidine	KI TBA	22	r.t.	\	\
8	tetrabutylammonium hydroxide	KI TBA	22	r.t.	23	NA
9	potassium tert-butoxide	KI TBA	16	r.t.	13	20

[a] The separation yield.

[b] Room temperature.

Table S2. Orthogonal tests for N1-alkylation of DHPMs in tetrabutylammonium hydroxide system.

**Entry**	**Mole ratio of halohydrocarbons**	**Mole equivalent of base**	**Temperature (°C)**	**Reaction time (h)**	**interval time[a] (h)**	**Phase transfer catalyst**	**Amount of solvent (ml/g)**	**N1-alkylation Yield (%)**	**N3-dialkylation Yield (%)**
1	1.5	1.7	15	24	1.5	TBA	20	17.2	NA
2	1.8	1.7	45	24	0.5	TBA	10	21.9	NA
3	1.5	2.0	45	24	1.5	\	10	21.2	NA
4	1.8	2.0	15	24	0.5	\	20	11.3	NA
5	1.5	1.7	45	16	0.5	\	20	21.6	NA
6	1.8	1.7	15	16	1.5	\	10	19.0	NA
7	1.5	2.0	15	16	0.5	TBA	10	15.4	NA
8	1.8	2.0	45	16	1.5	TBA	20	35.1	NA
Sum of low dose yields	77.5	79.7	62.9	91.1	70.2	89.6	77.5		
Sum of high dose yields	85.2	83.0	99.8	71.6	92.5	73.1	85.2		
Range(R)	11.9	3.3	36.9	19.5	22.3	16.5	7.7		

[a] interval time between compound **1** and compound **2**.

Table S3. Dataset used for Pharmacophore analysis.

**Cpd.**	**Structure**	**In Vitro Cell Viability**
**1d**	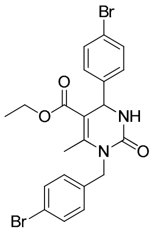	9.72 [a]

**1h**	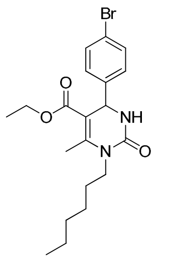	9.30 [a]
**1j**	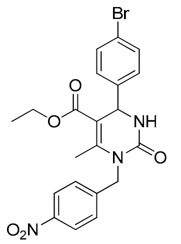	39.31 [b]
**3d**	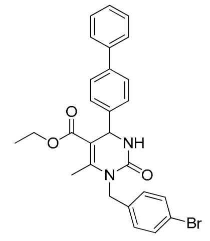	6.36 [a]
**3g**	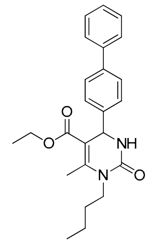	7.32 [a]
**3h**	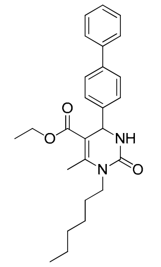	48.79 [b]
**1**	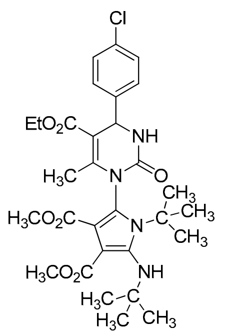	10.36 [a]

**2**	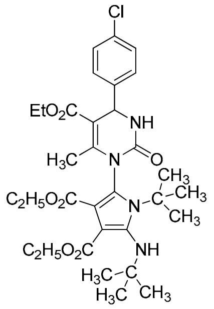	9.90 [a]
**3**	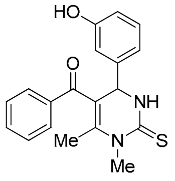	0.68 [a]
**4**	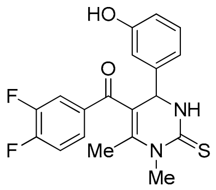	0.48 [a]
**5**	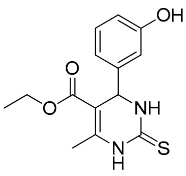	1.41 [a]
**6**	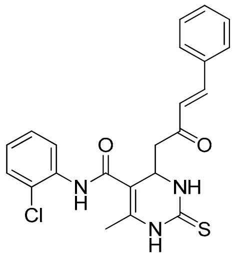	71 [b]
**7**	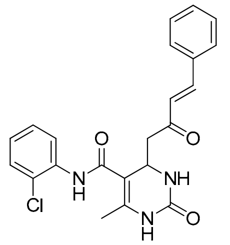	79 [b]

[a] Compounds **1d**, **1h**, **3d**, **3g, 1, 2, 3, 4** and **5** were IC_{50} values (μM). [b] Compounds **1j**, **3h, 6** and **7** were cytotoxic activity (%).
