# Supplementary figures and images for: Synthesis, Characterization, and Anticancer Activities Evaluation of Compounds Derived from 3,4-Dihydropyrimidin-2(1H)-one
Source: Molecules. 2019 Mar 3;24(5):891. doi: 10.3390/molecules24050891 (PMC6429579; doi:10.3390/molecules24050891)

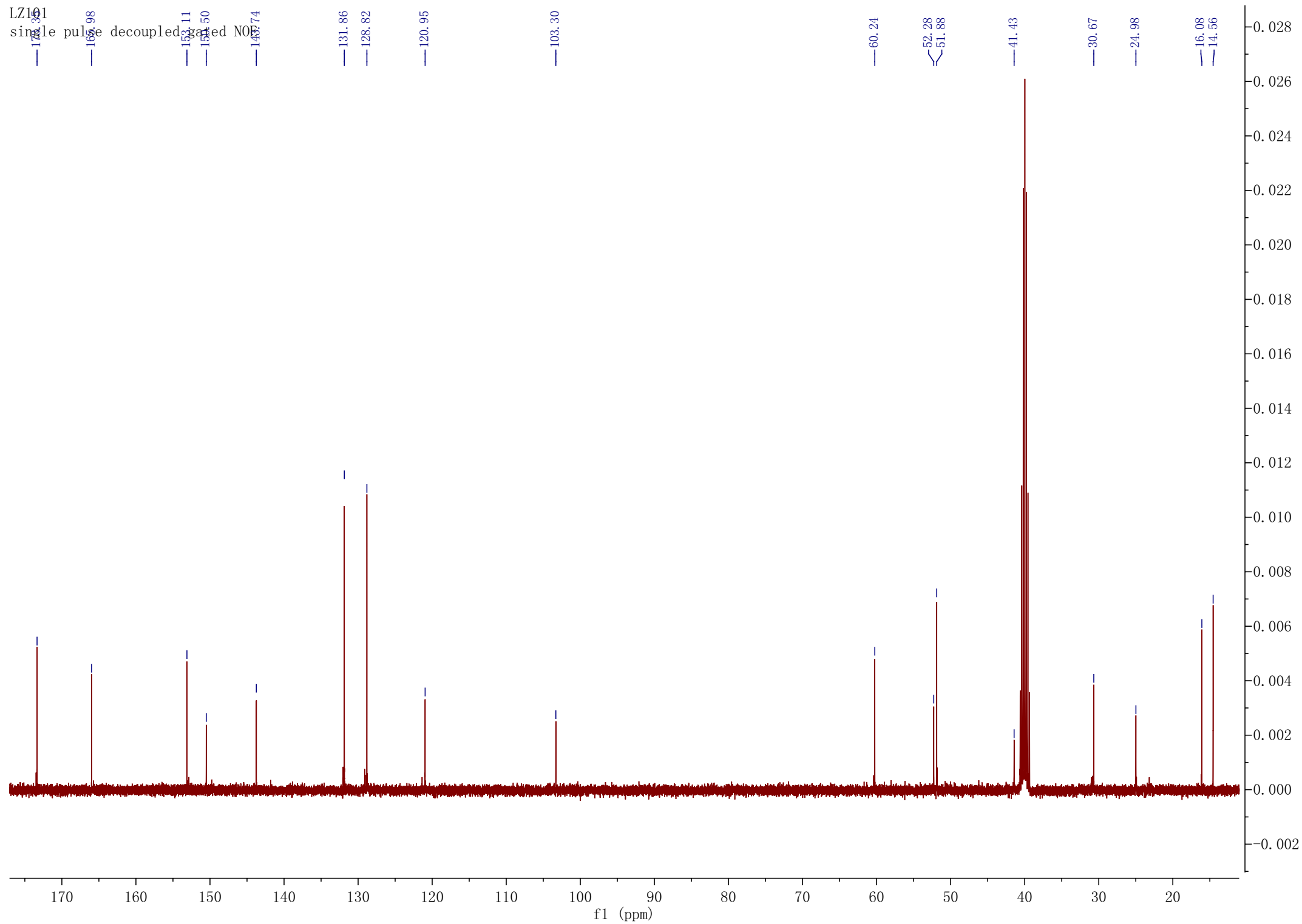

Supplement: Supplementary file 1 [file molecules-24-00891-s001.zip › molecules-433653-suppl/13C-NMR/1a.pdf]

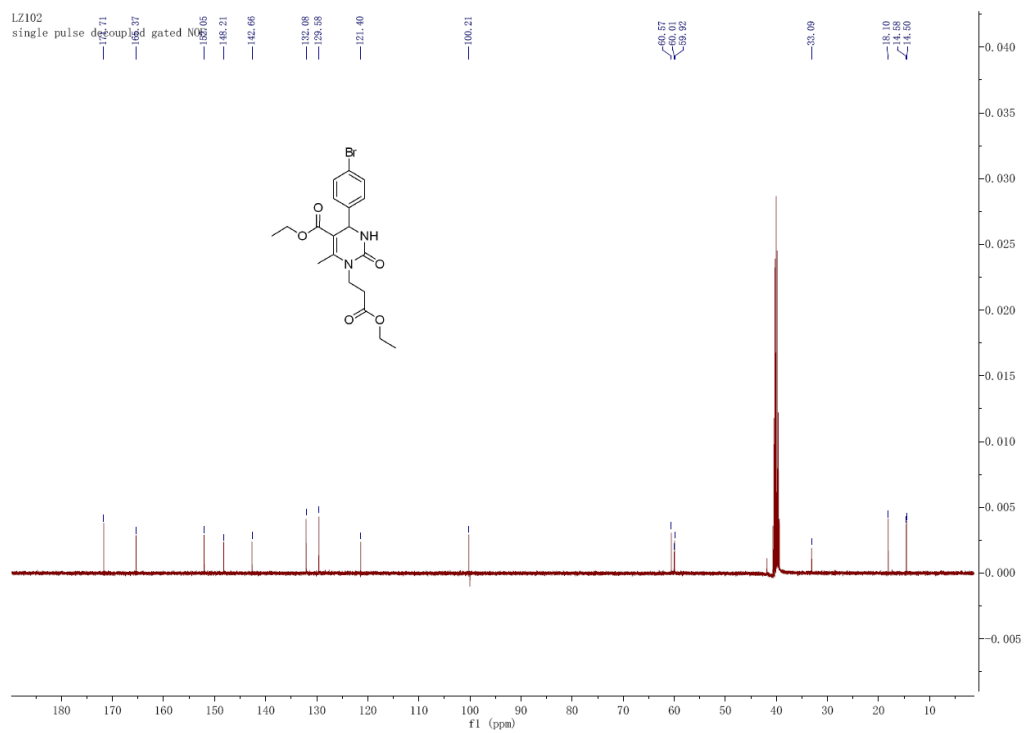

Supplement: Supplementary file 1 [file molecules-24-00891-s001.zip › molecules-433653-suppl/13C-NMR/1b.pdf]

LZ103  
single pulse decoupled gated NOE

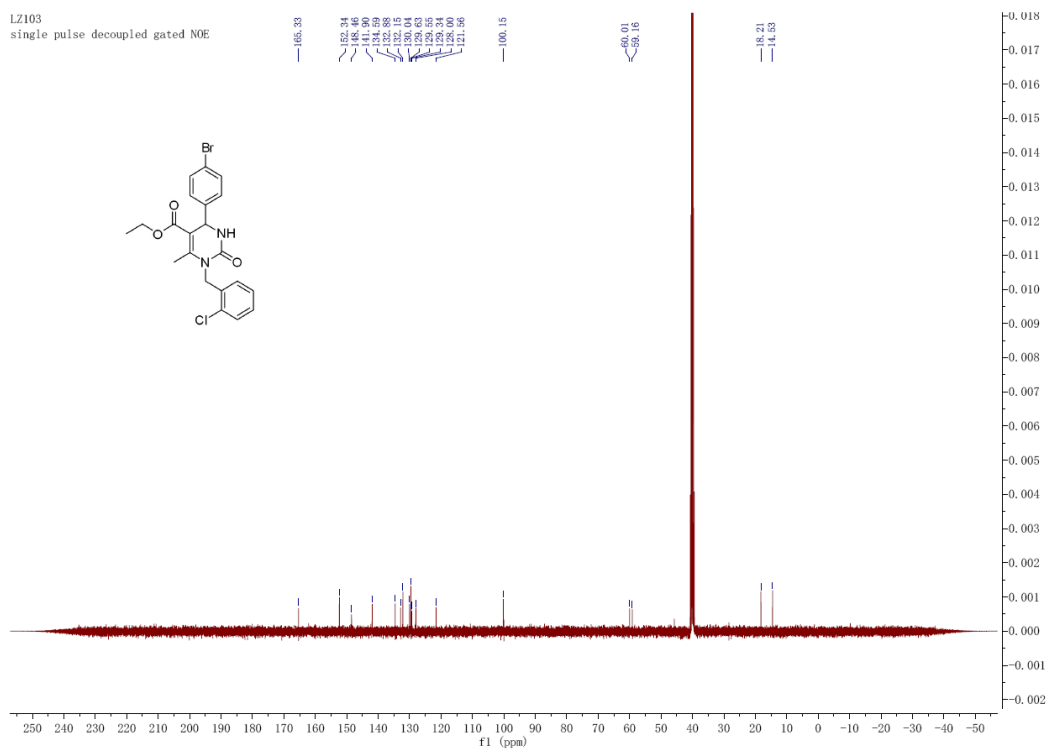

Supplement: Supplementary file 1 [file molecules-24-00891-s001.zip › molecules-433653-suppl/13C-NMR/1c.pdf]

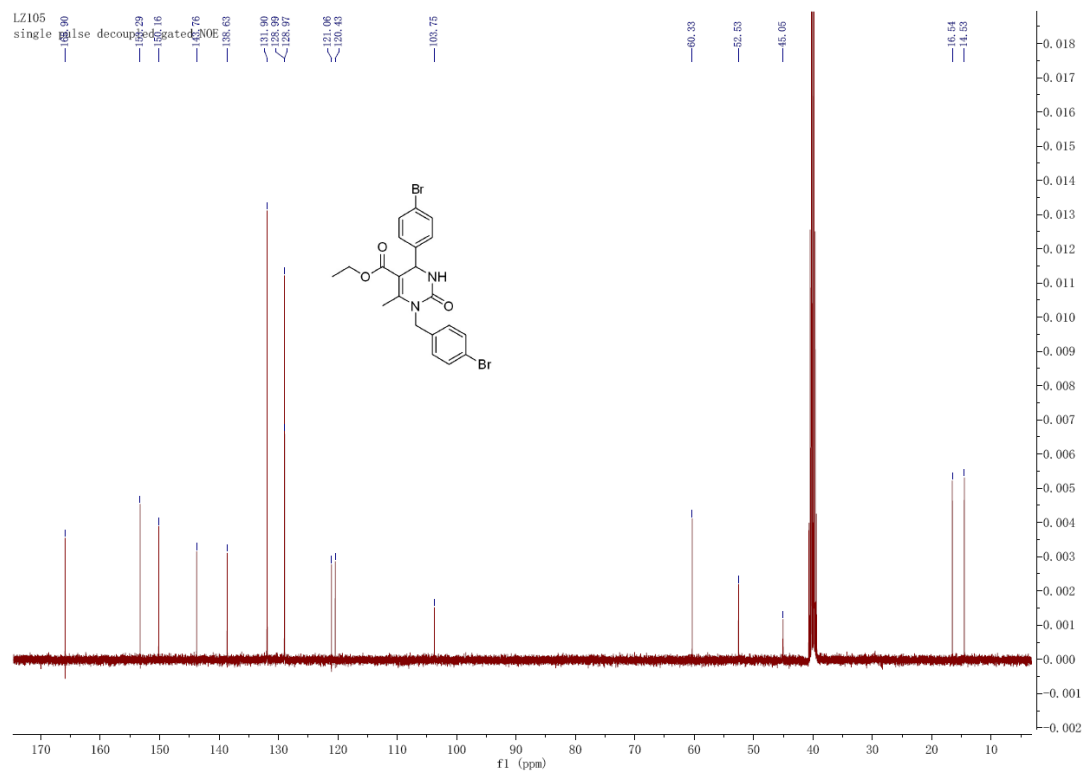

Supplement: Supplementary file 1 [file molecules-24-00891-s001.zip › molecules-433653-suppl/13C-NMR/1d.pdf]

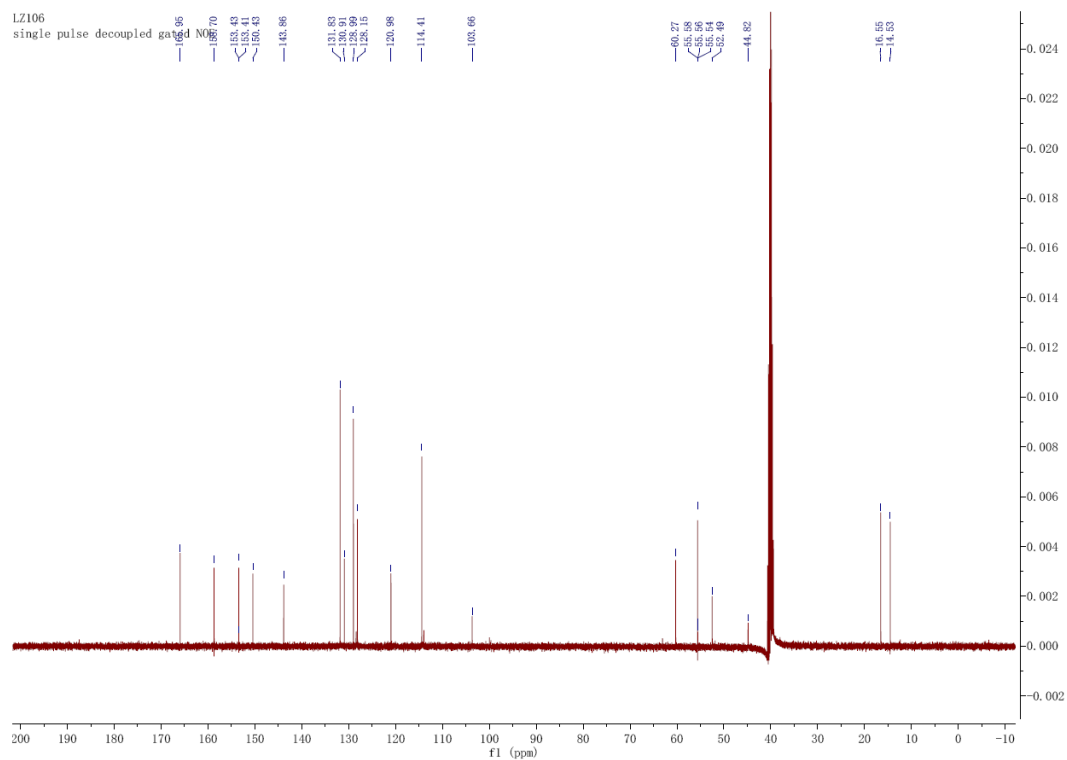

Supplement: Supplementary file 1 [file molecules-24-00891-s001.zip › molecules-433653-suppl/13C-NMR/1e.pdf]

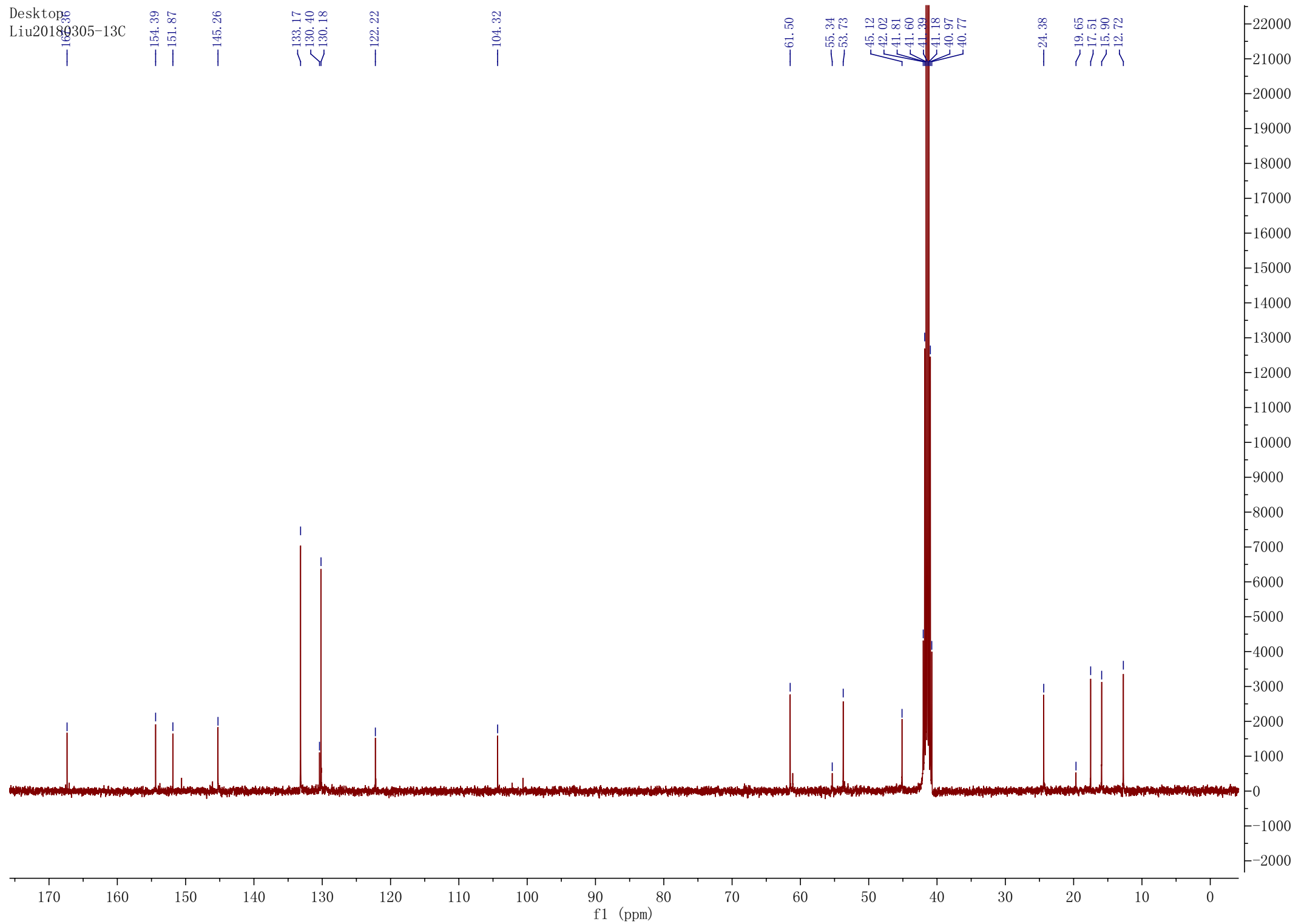

Supplement: Supplementary file 1 [file molecules-24-00891-s001.zip › molecules-433653-suppl/13C-NMR/1f.pdf]

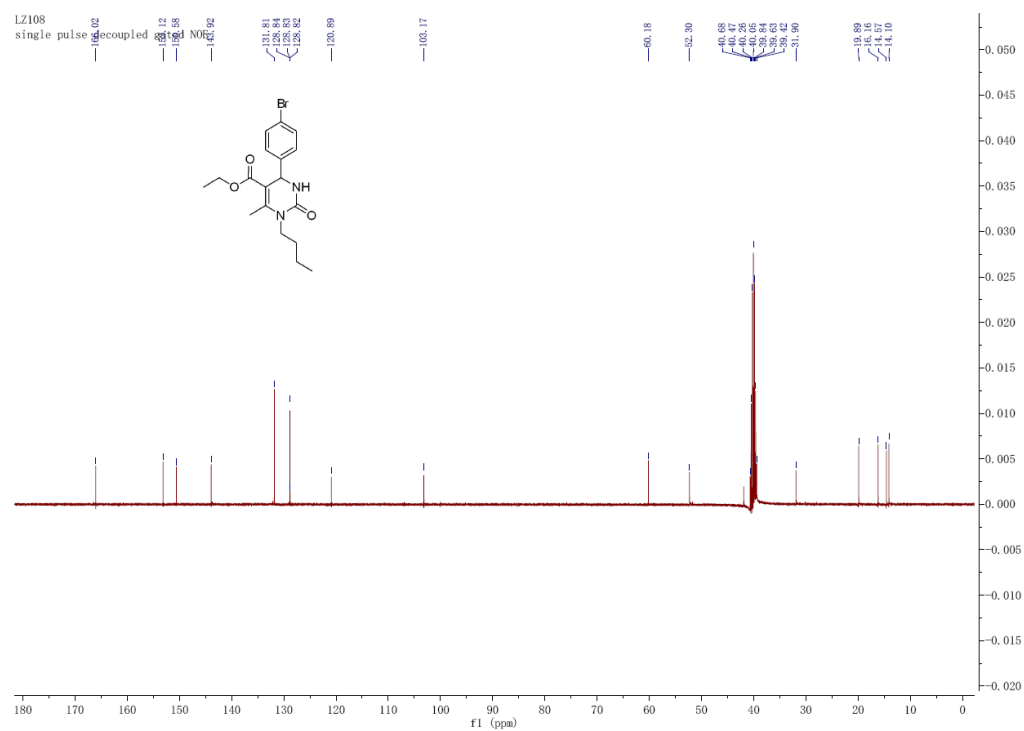

Supplement: Supplementary file 1 [file molecules-24-00891-s001.zip › molecules-433653-suppl/13C-NMR/1g.pdf]

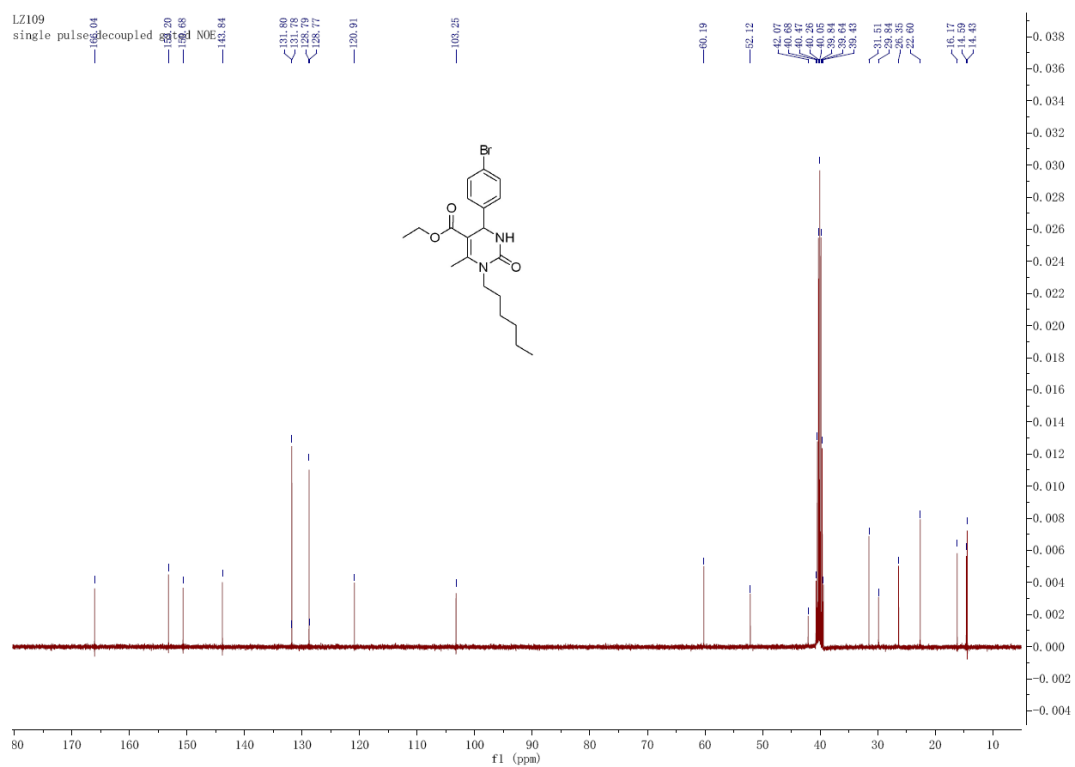

Supplement: Supplementary file 1 [file molecules-24-00891-s001.zip › molecules-433653-suppl/13C-NMR/1h.pdf]

LZ110

single pulse decoupled gated NOE

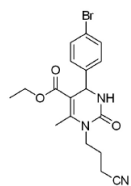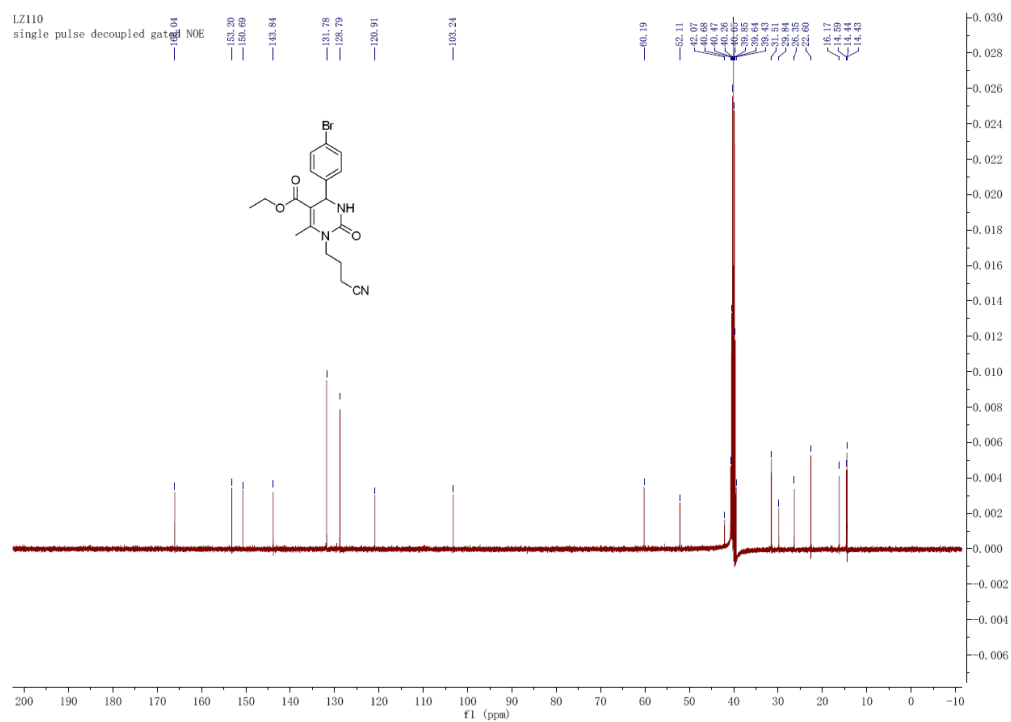

Supplement: Supplementary file 1 [file molecules-24-00891-s001.zip › molecules-433653-suppl/13C-NMR/1i.pdf]

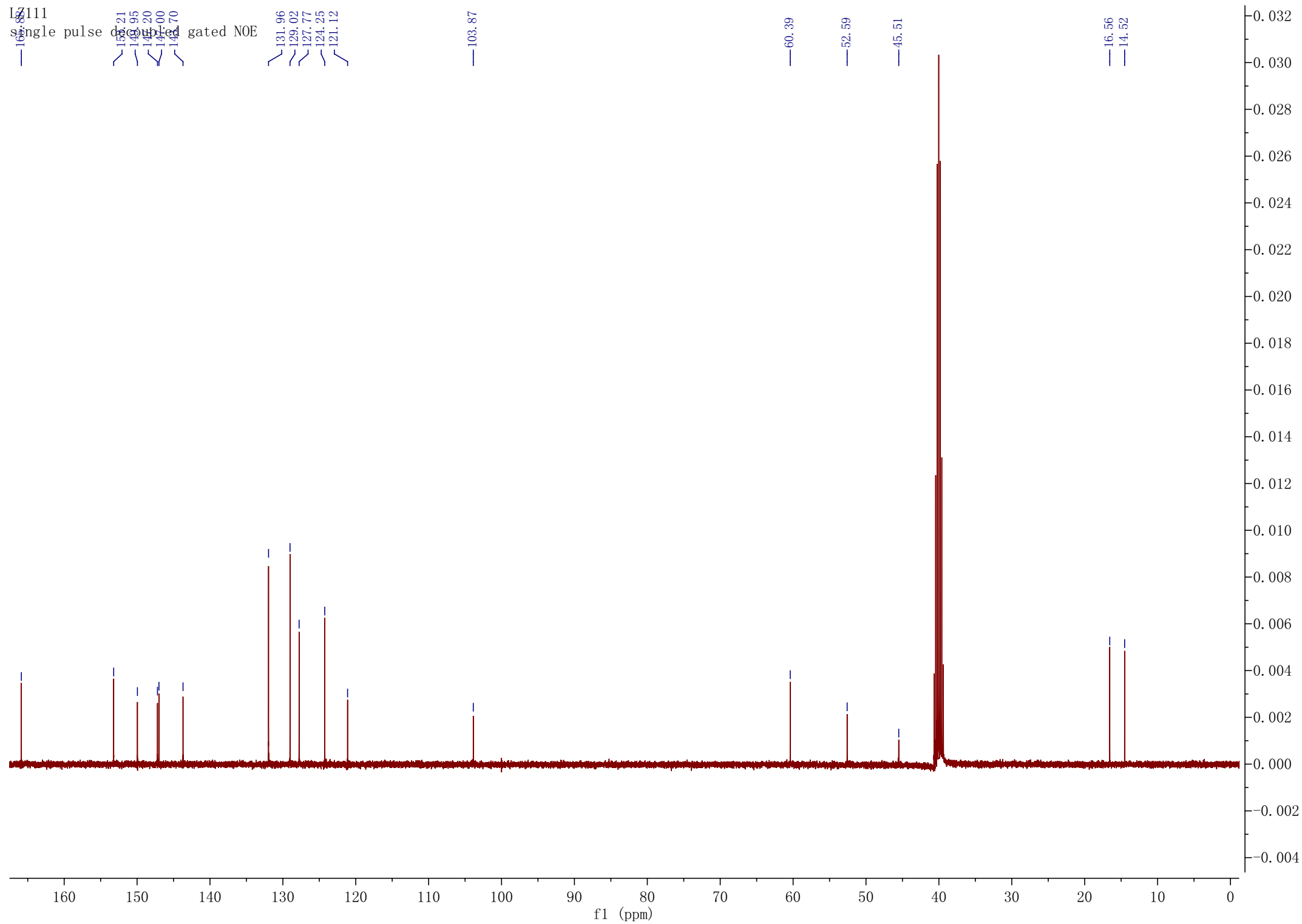

Supplement: Supplementary file 1 [file molecules-24-00891-s001.zip › molecules-433653-suppl/13C-NMR/1j.pdf]

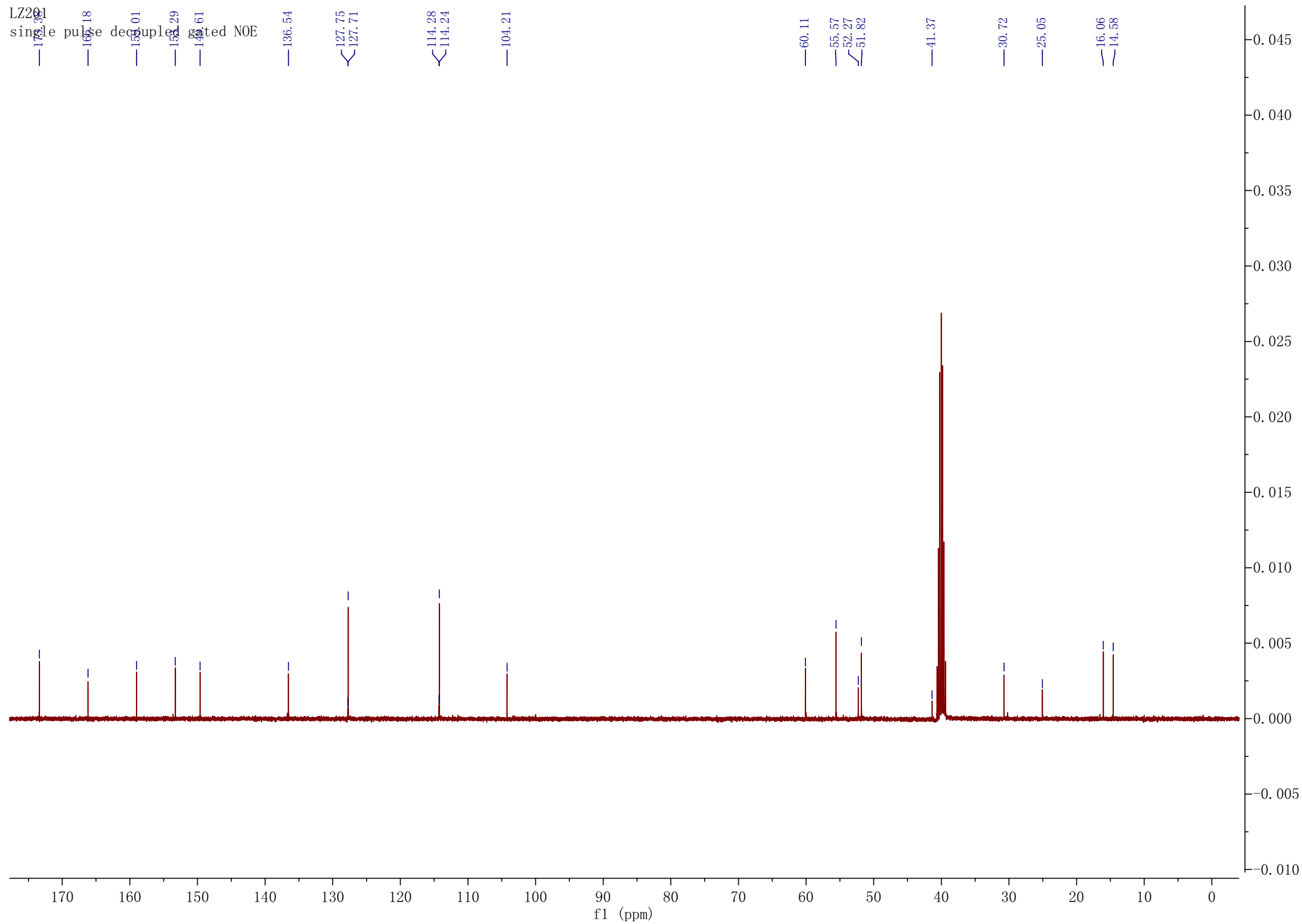

Supplement: Supplementary file 1 [file molecules-24-00891-s001.zip › molecules-433653-suppl/13C-NMR/2a.pdf]

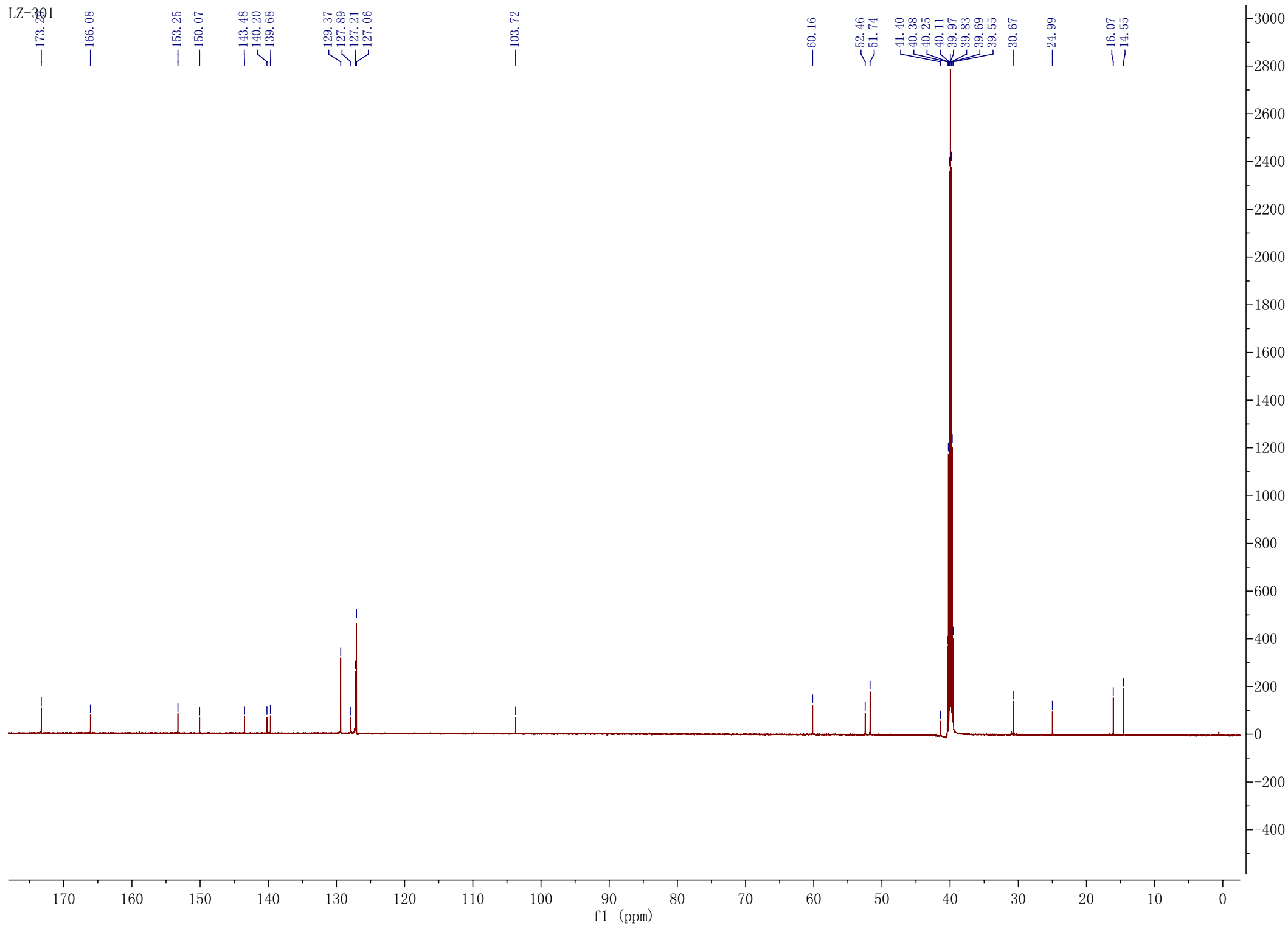

Supplement: Supplementary file 1 [file molecules-24-00891-s001.zip › molecules-433653-suppl/13C-NMR/3a.pdf]

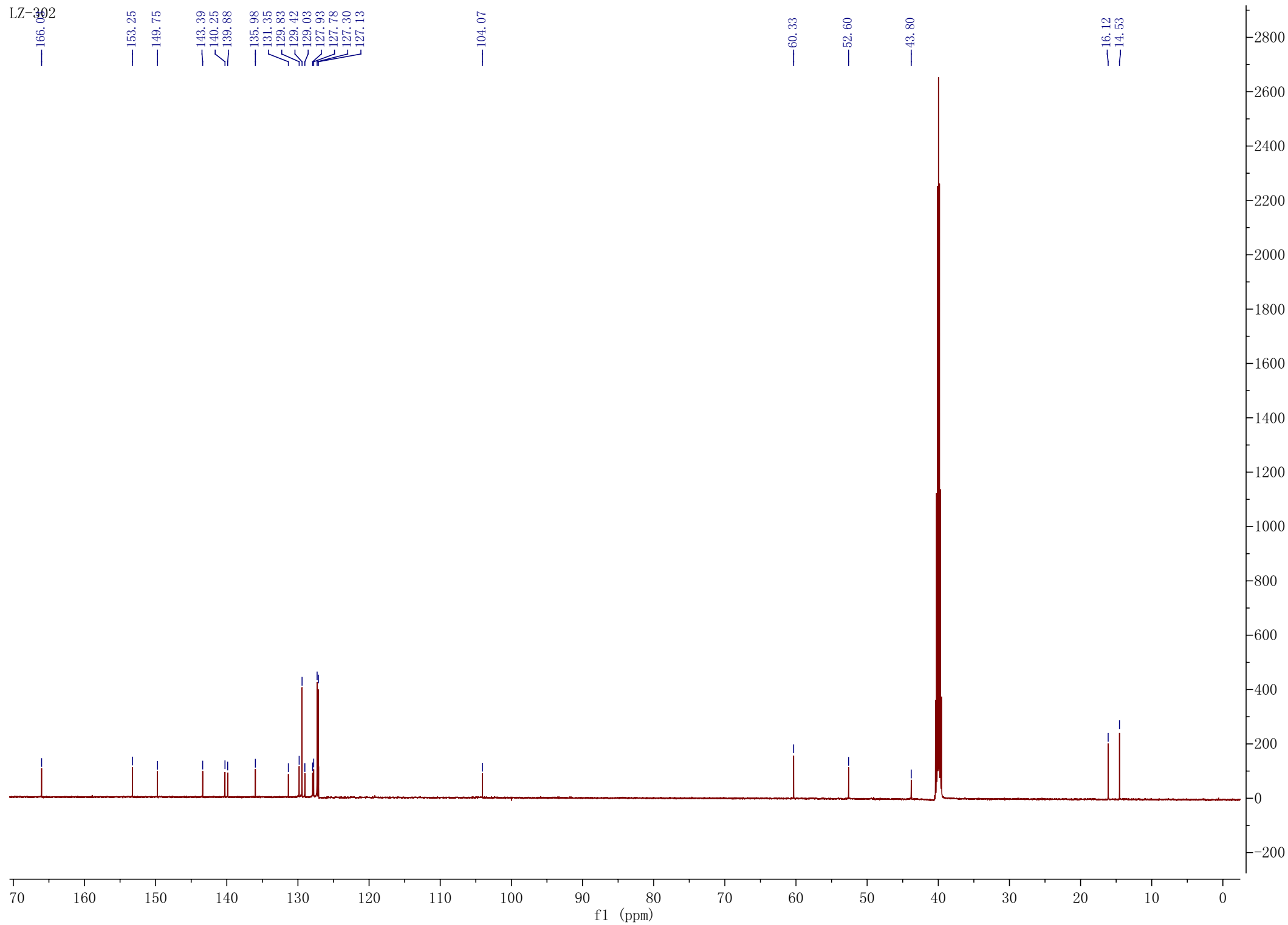

Supplement: Supplementary file 1 [file molecules-24-00891-s001.zip › molecules-433653-suppl/13C-NMR/3c.pdf]

LZ-303

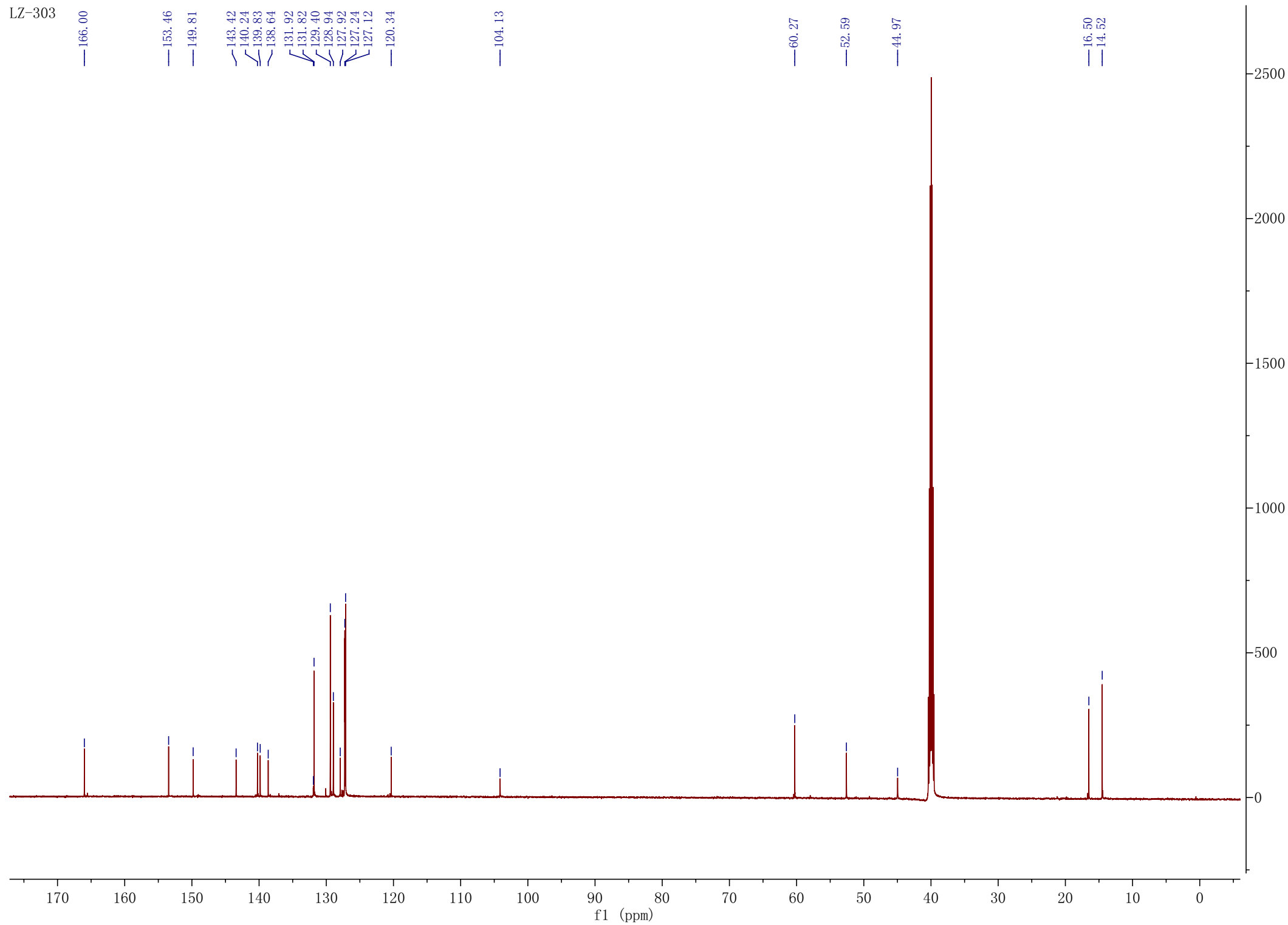

Supplement: Supplementary file 1 [file molecules-24-00891-s001.zip › molecules-433653-suppl/13C-NMR/3d.pdf]

LZ-304

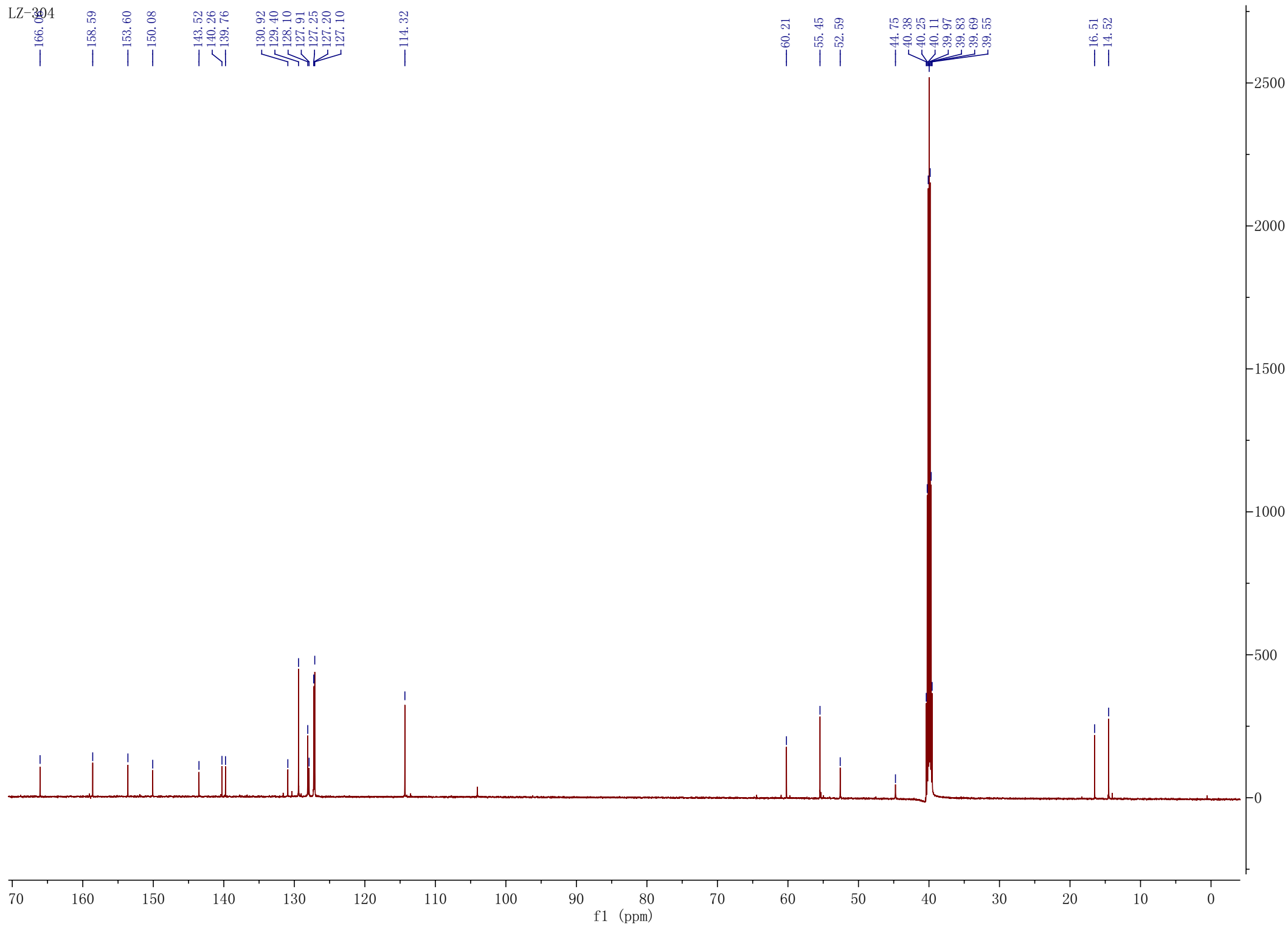

Supplement: Supplementary file 1 [file molecules-24-00891-s001.zip › molecules-433653-suppl/13C-NMR/3e.pdf]

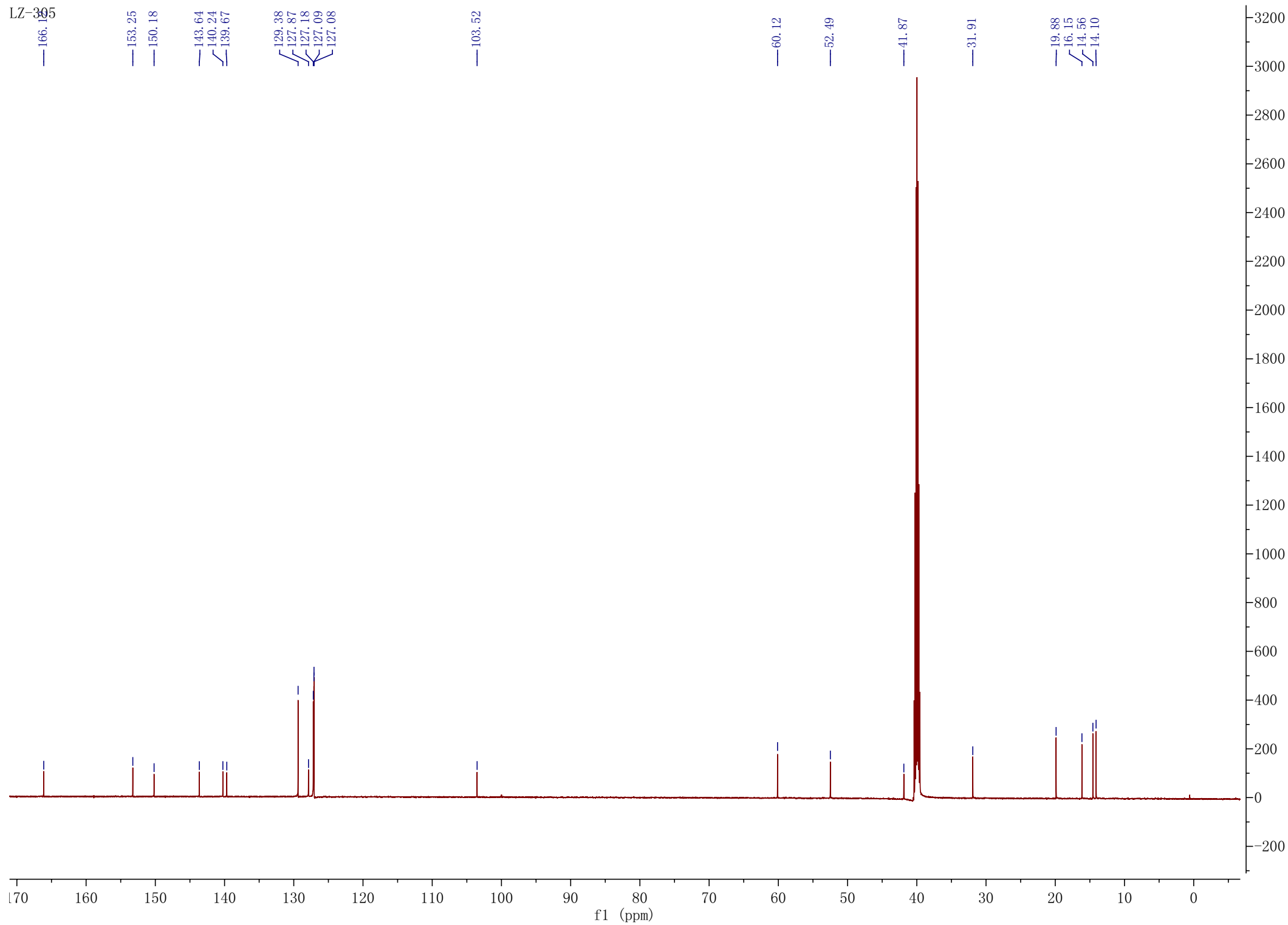

Supplement: Supplementary file 1 [file molecules-24-00891-s001.zip › molecules-433653-suppl/13C-NMR/3g.pdf]

LZ-306

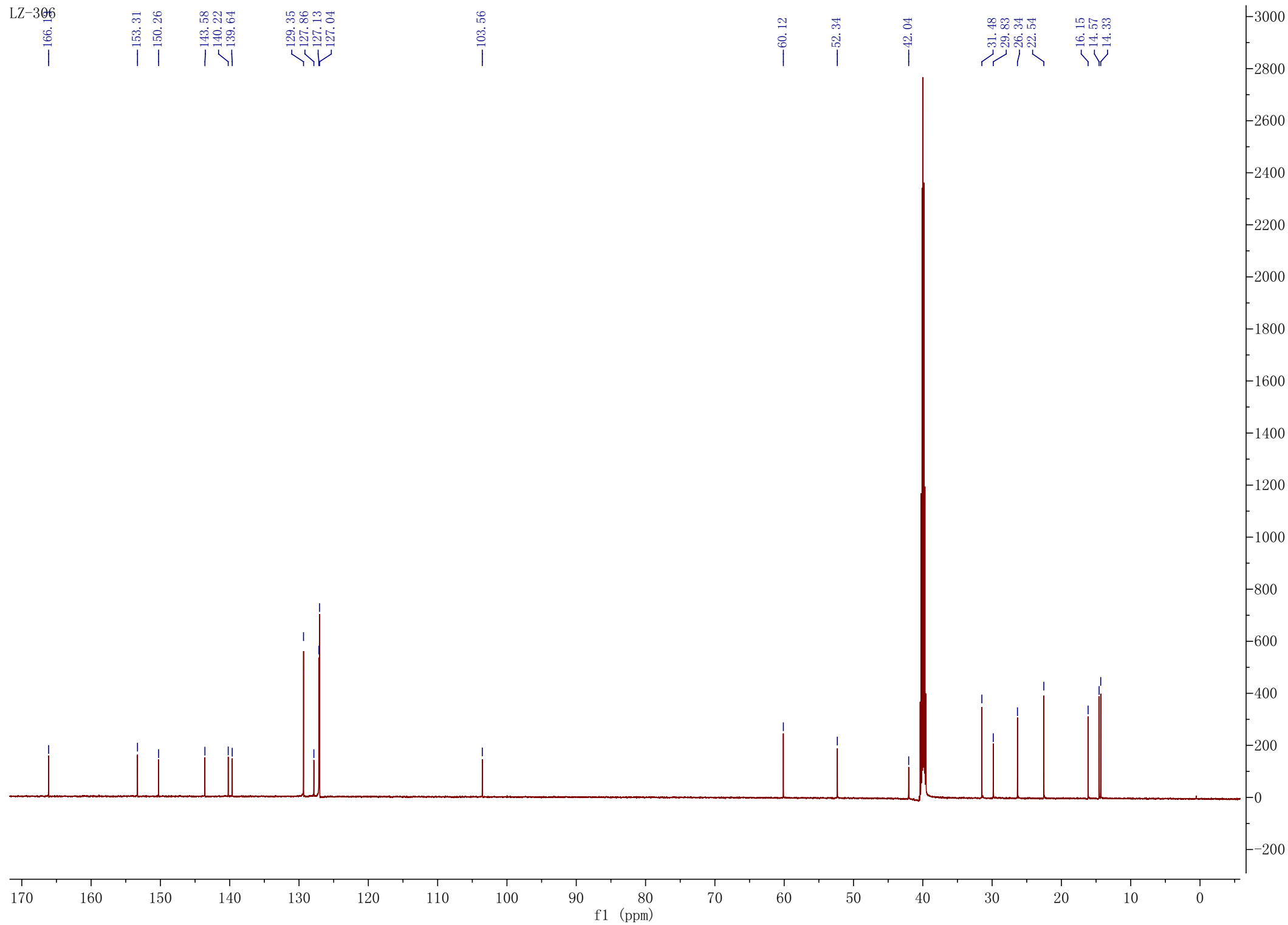

Supplement: Supplementary file 1 [file molecules-24-00891-s001.zip › molecules-433653-suppl/13C-NMR/3h.pdf]

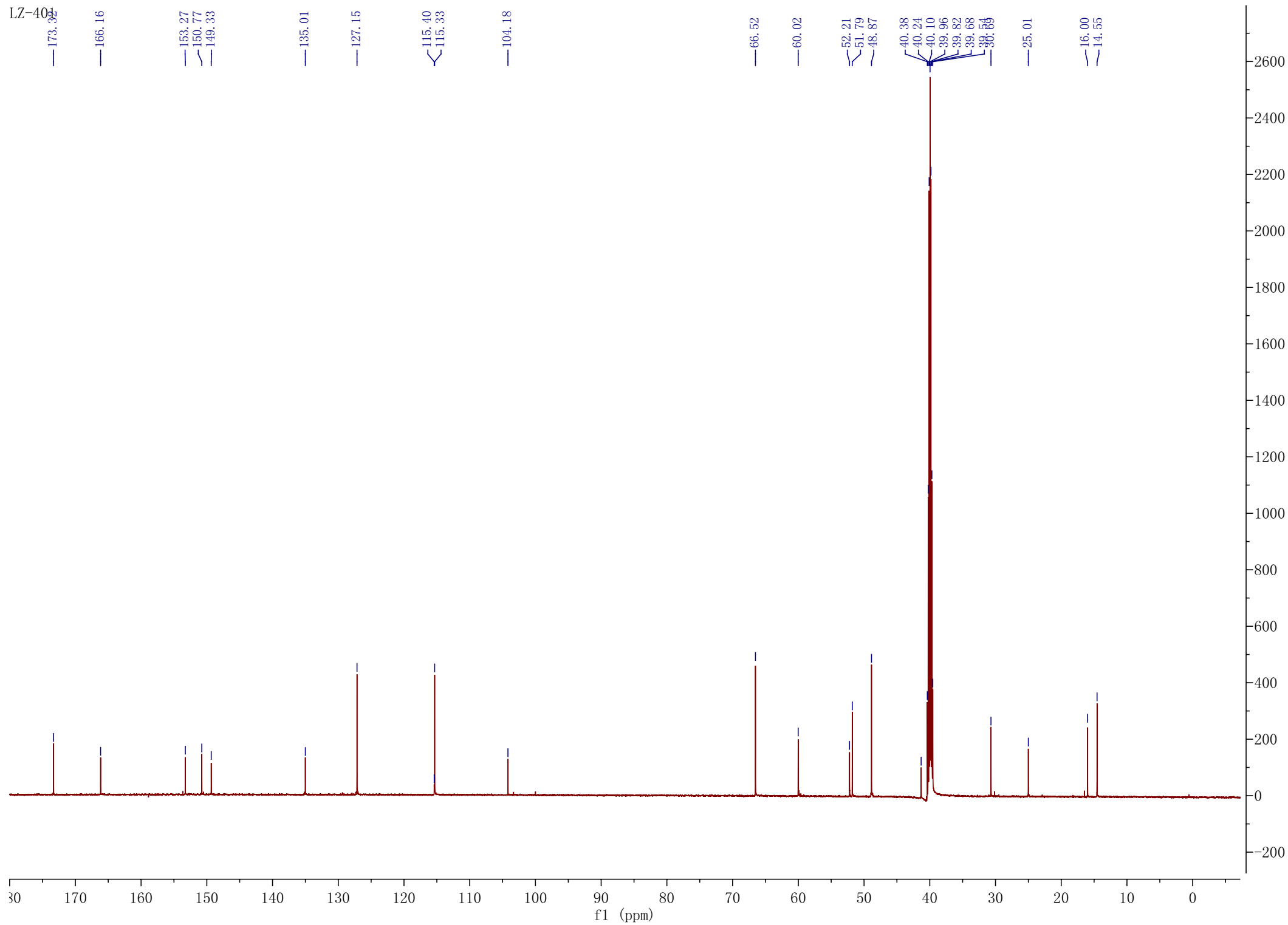

Supplement: Supplementary file 1 [file molecules-24-00891-s001.zip › molecules-433653-suppl/13C-NMR/4a.pdf]

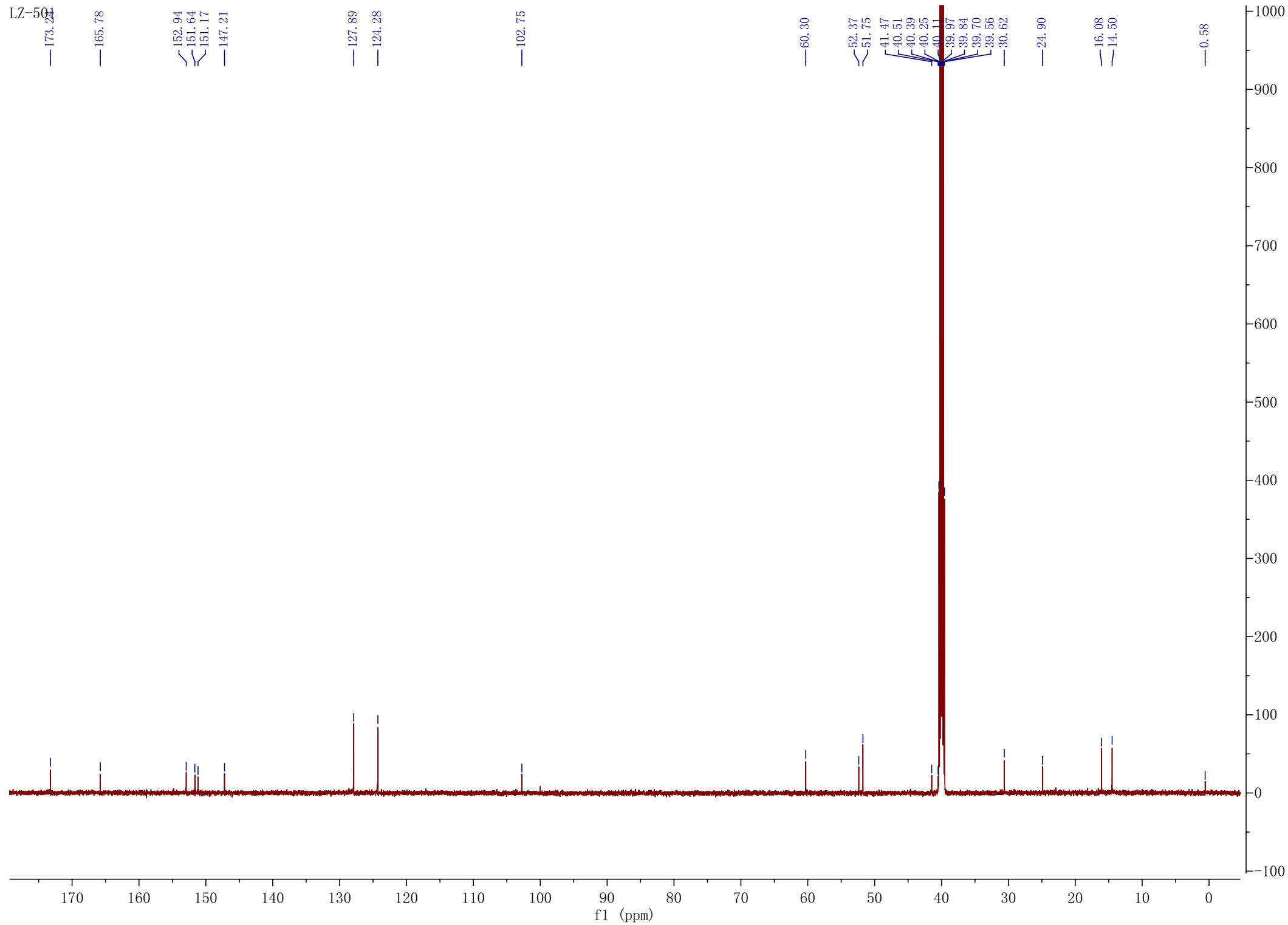

Supplement: Supplementary file 1 [file molecules-24-00891-s001.zip › molecules-433653-suppl/13C-NMR/5a.pdf]

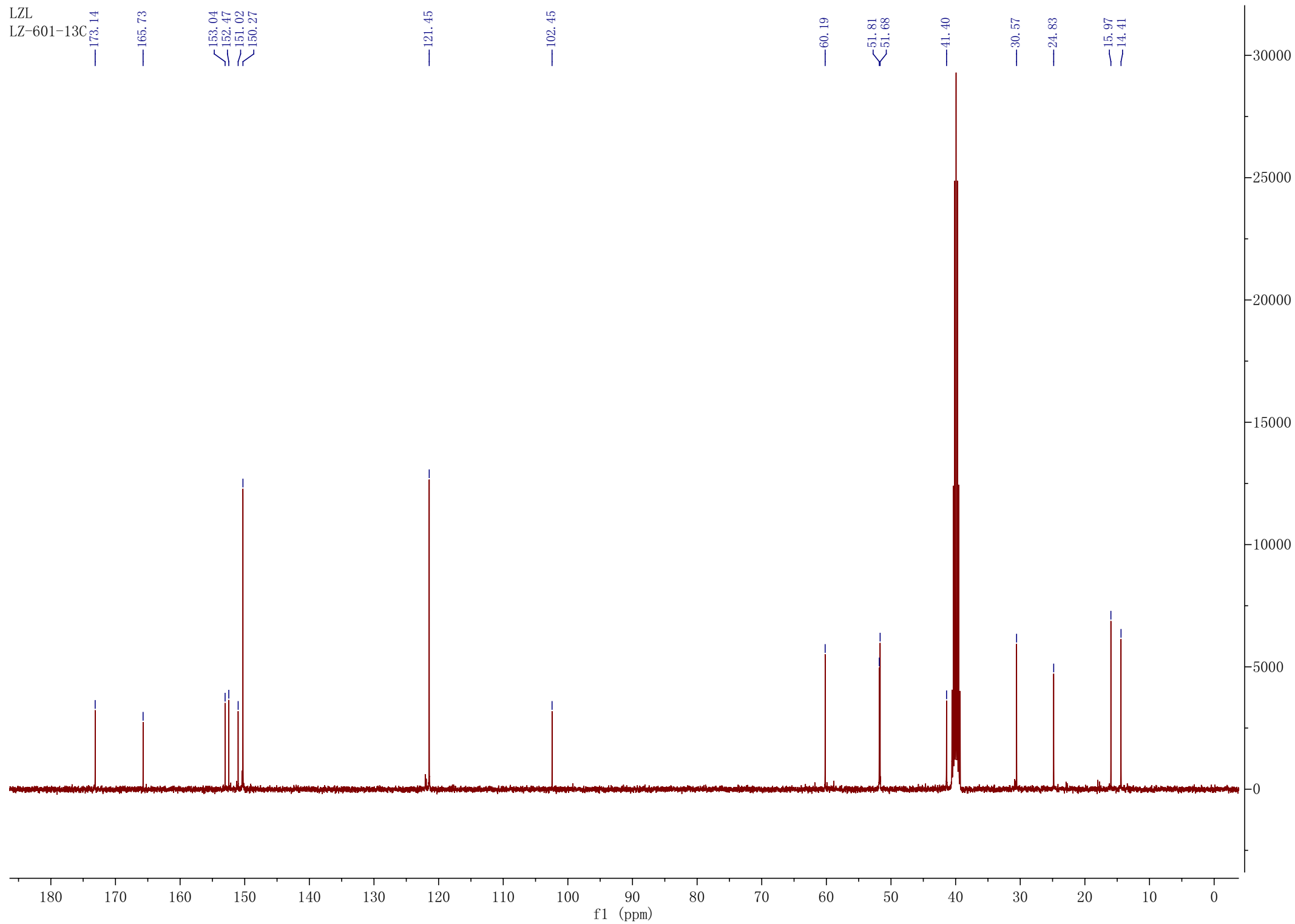

Supplement: Supplementary file 1 [file molecules-24-00891-s001.zip › molecules-433653-suppl/13C-NMR/6a.pdf]

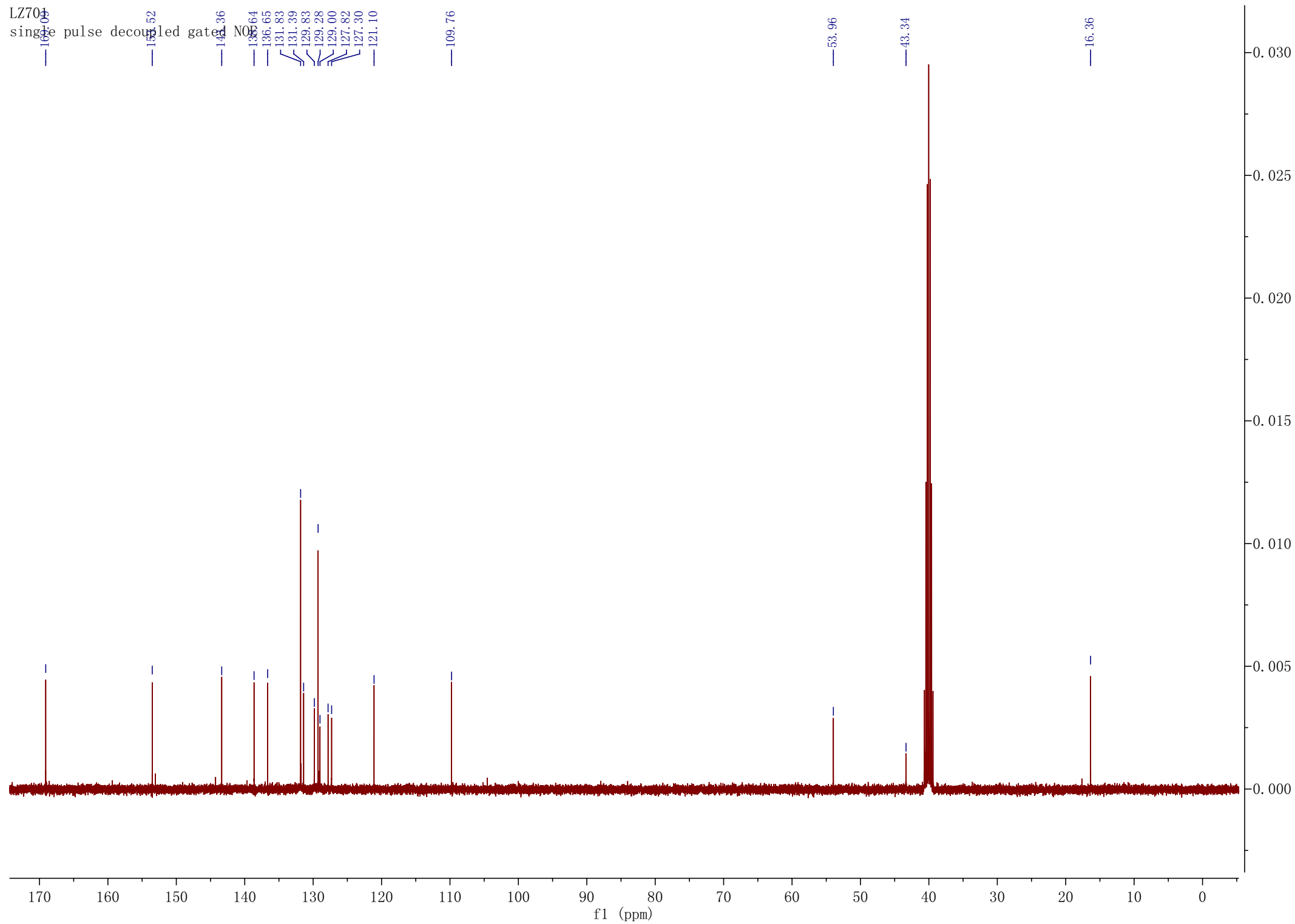

Supplement: Supplementary file 1 [file molecules-24-00891-s001.zip › molecules-433653-suppl/13C-NMR/7c.pdf]

LZ702

single pulse decoupled gated NOE

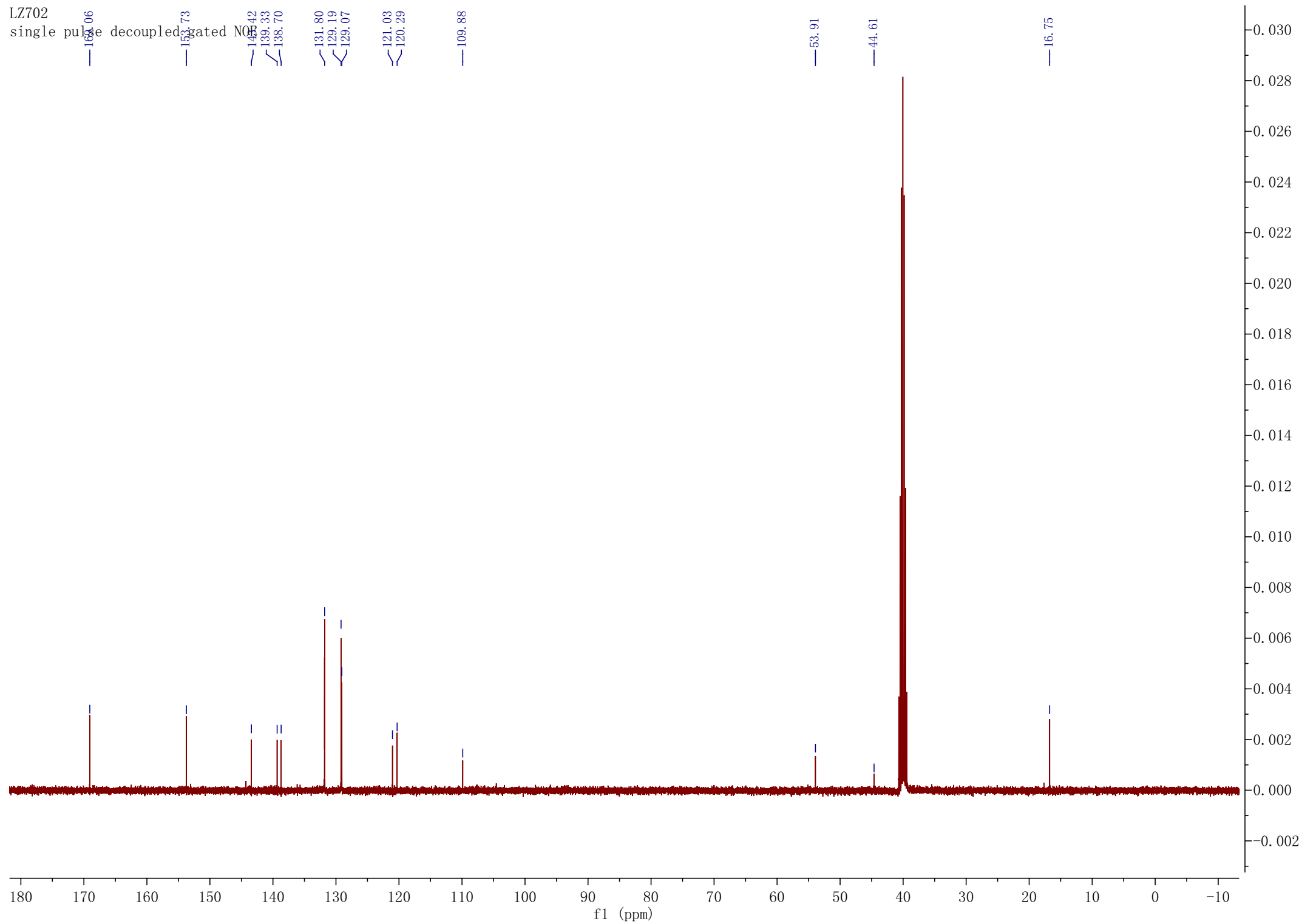

Supplement: Supplementary file 1 [file molecules-24-00891-s001.zip › molecules-433653-suppl/13C-NMR/7d.pdf]

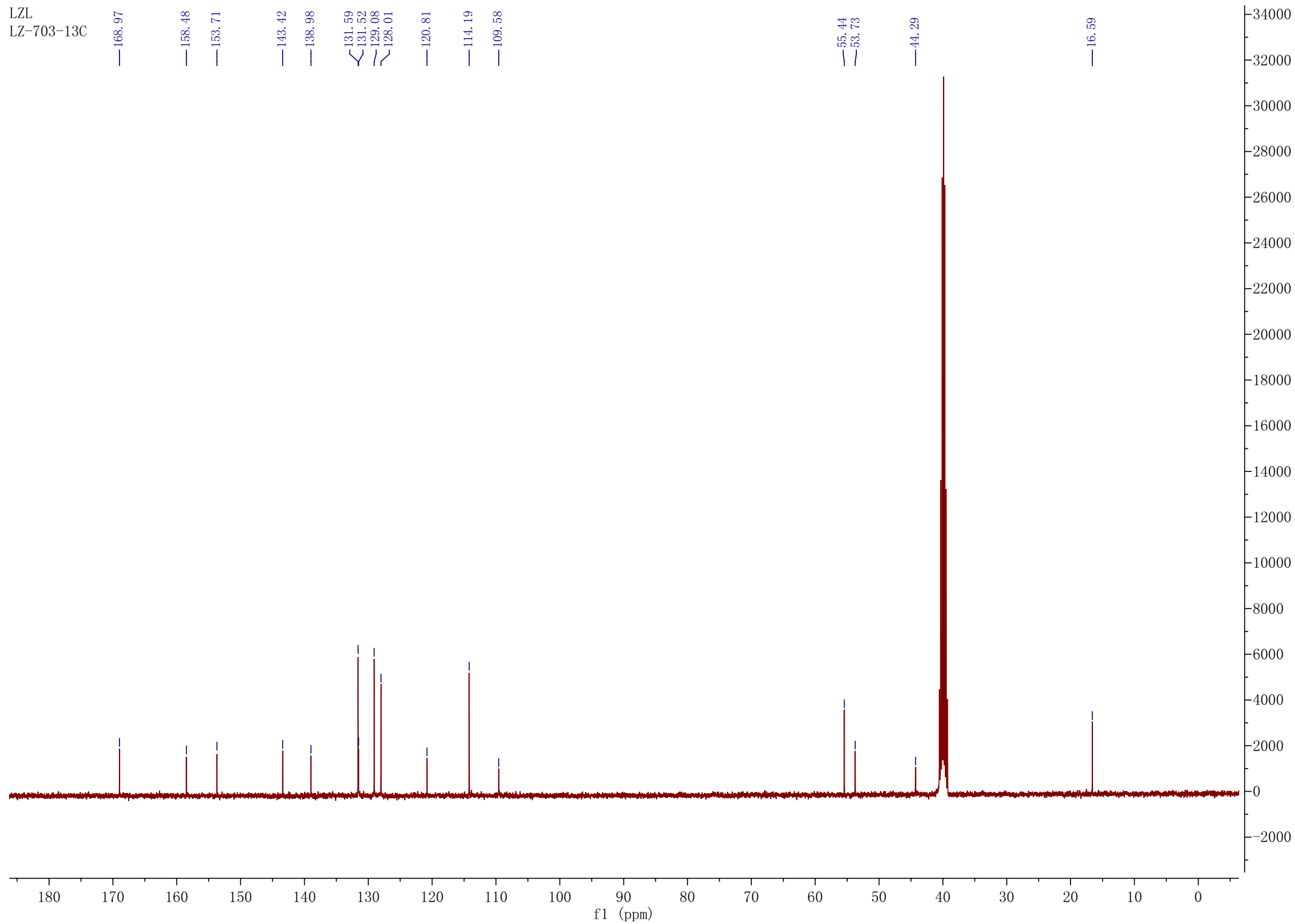

Supplement: Supplementary file 1 [file molecules-24-00891-s001.zip › molecules-433653-suppl/13C-NMR/7e.pdf]

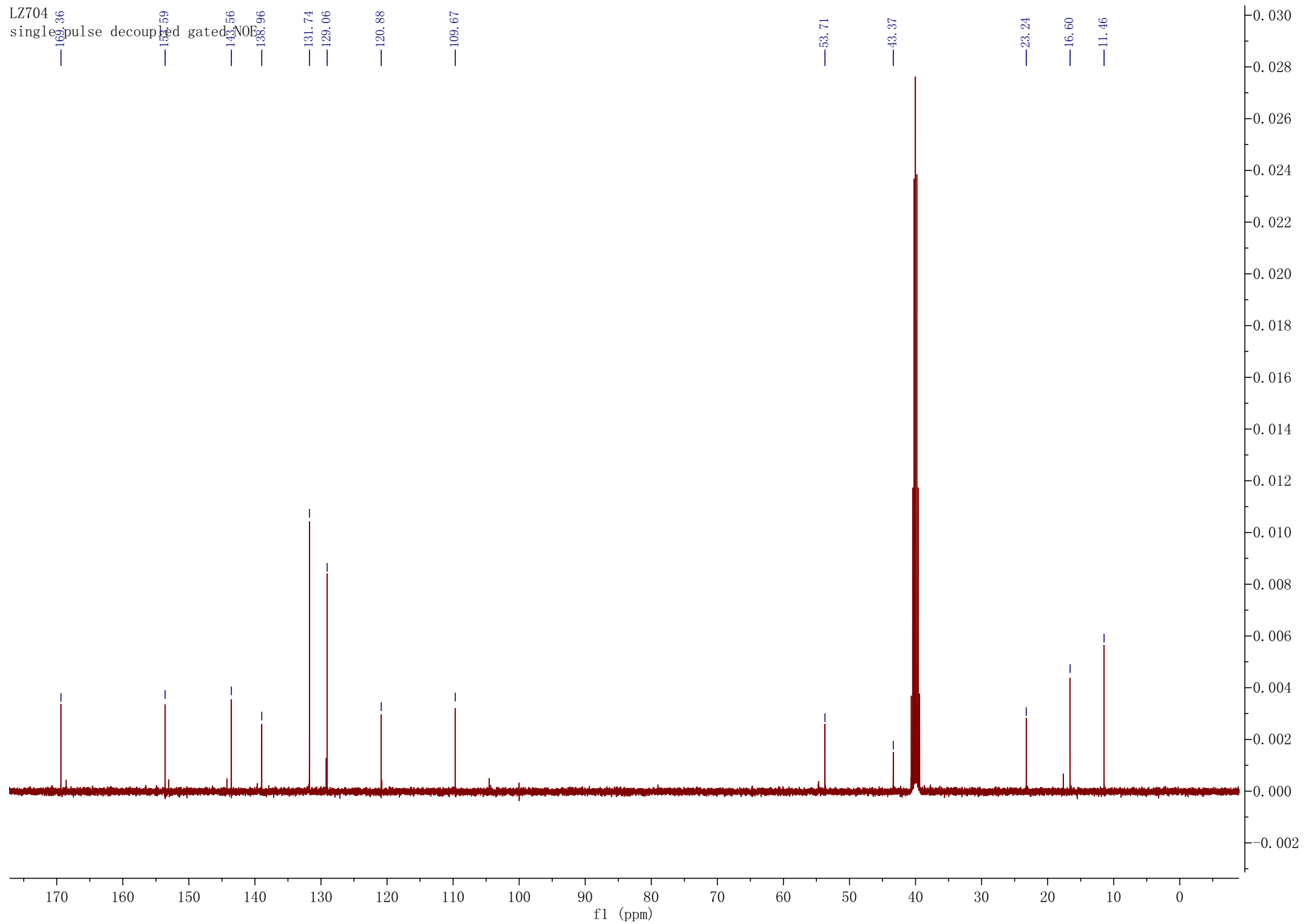

Supplement: Supplementary file 1 [file molecules-24-00891-s001.zip › molecules-433653-suppl/13C-NMR/7f.pdf]

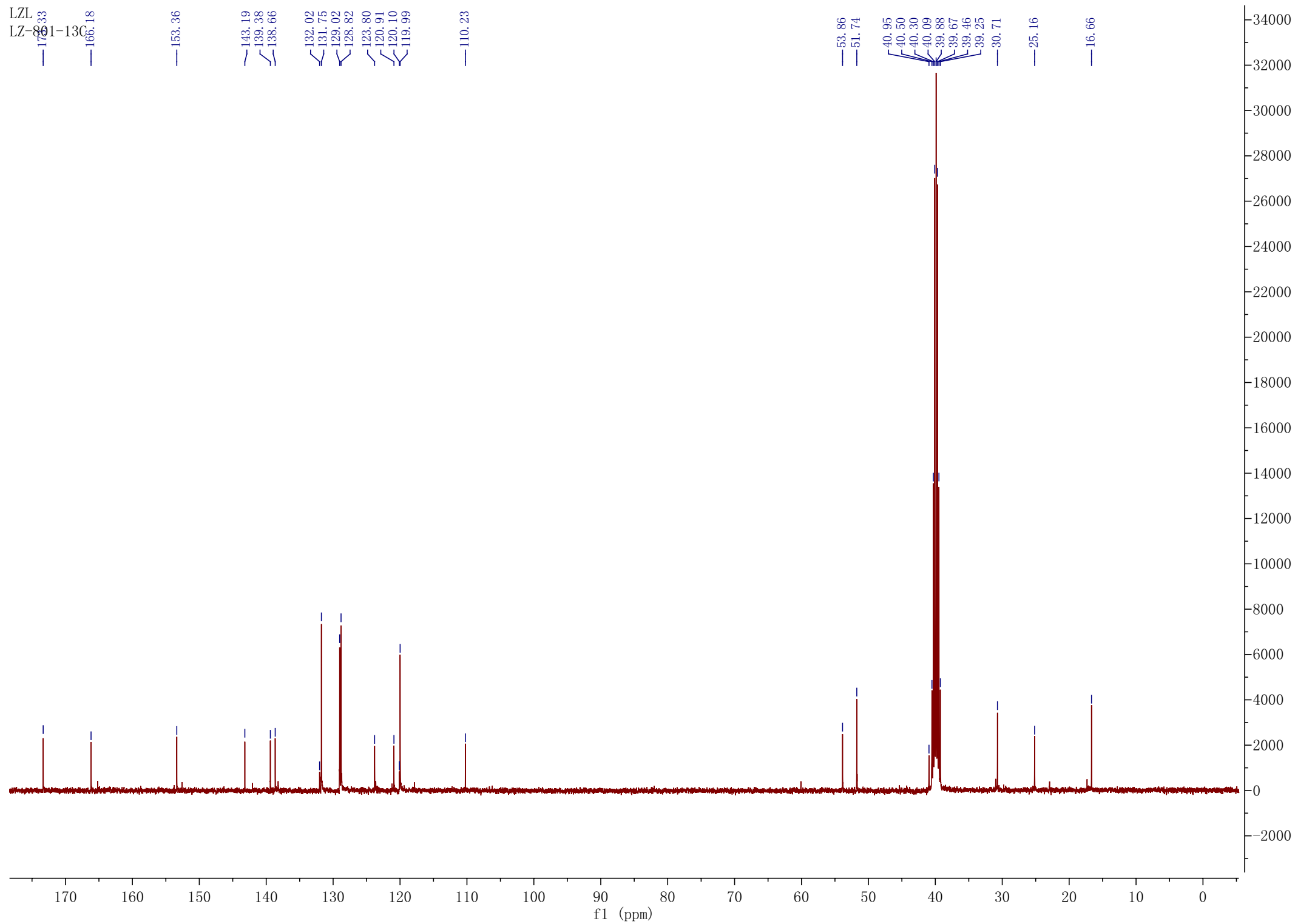

Supplement: Supplementary file 1 [file molecules-24-00891-s001.zip › molecules-433653-suppl/13C-NMR/8a.pdf]

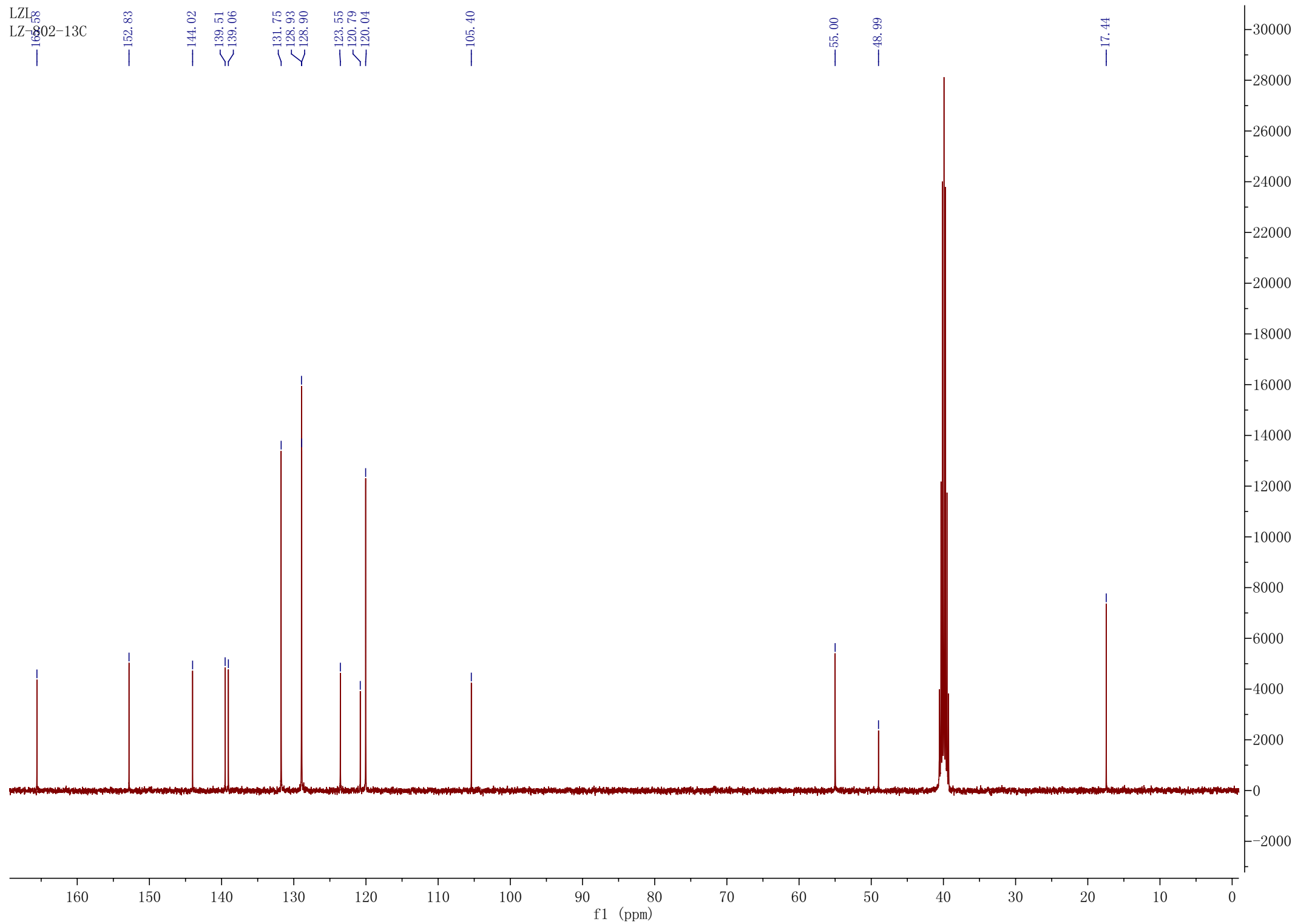

Supplement: Supplementary file 1 [file molecules-24-00891-s001.zip › molecules-433653-suppl/13C-NMR/8d.pdf]

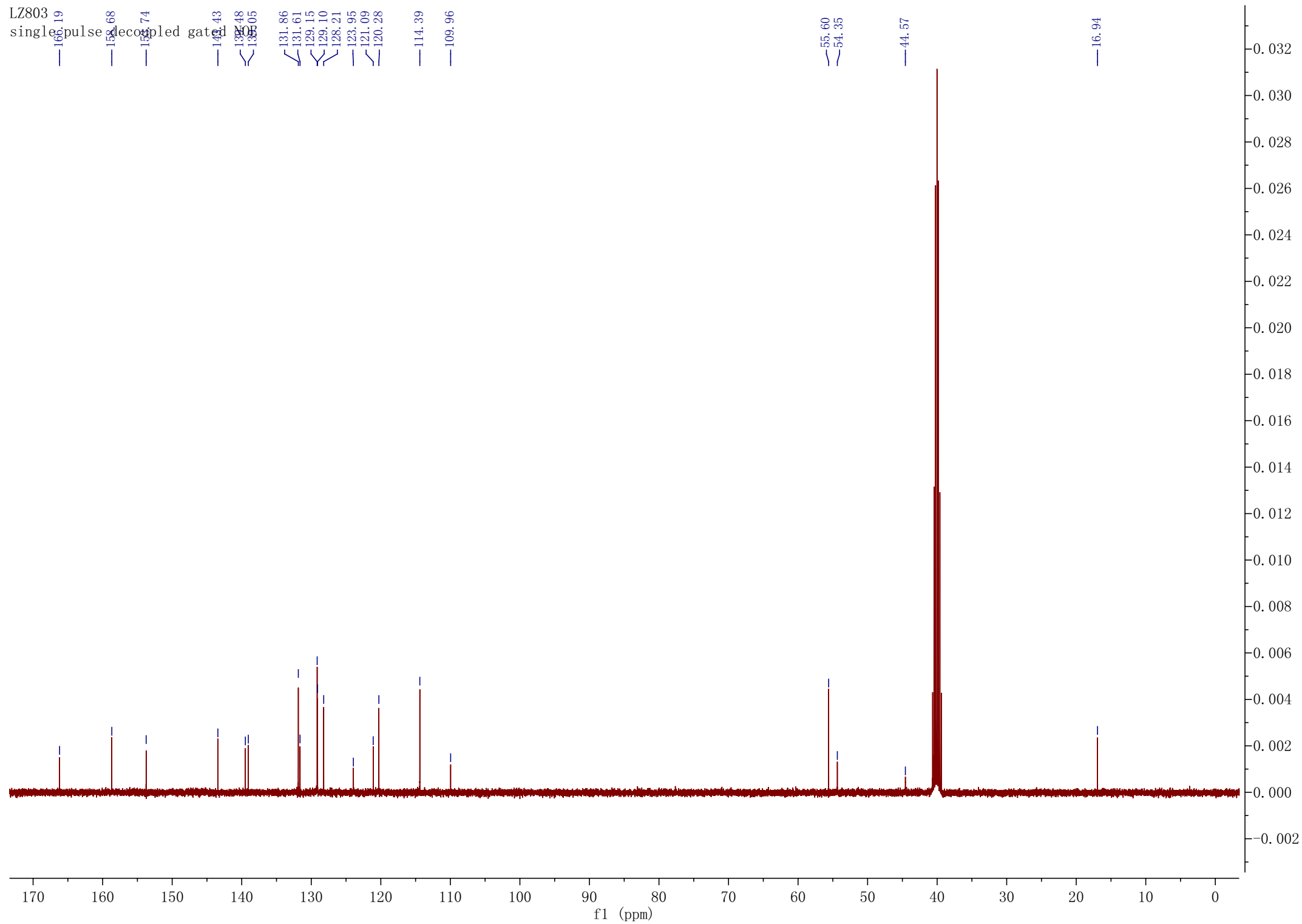

Supplement: Supplementary file 1 [file molecules-24-00891-s001.zip › molecules-433653-suppl/13C-NMR/8e.pdf]

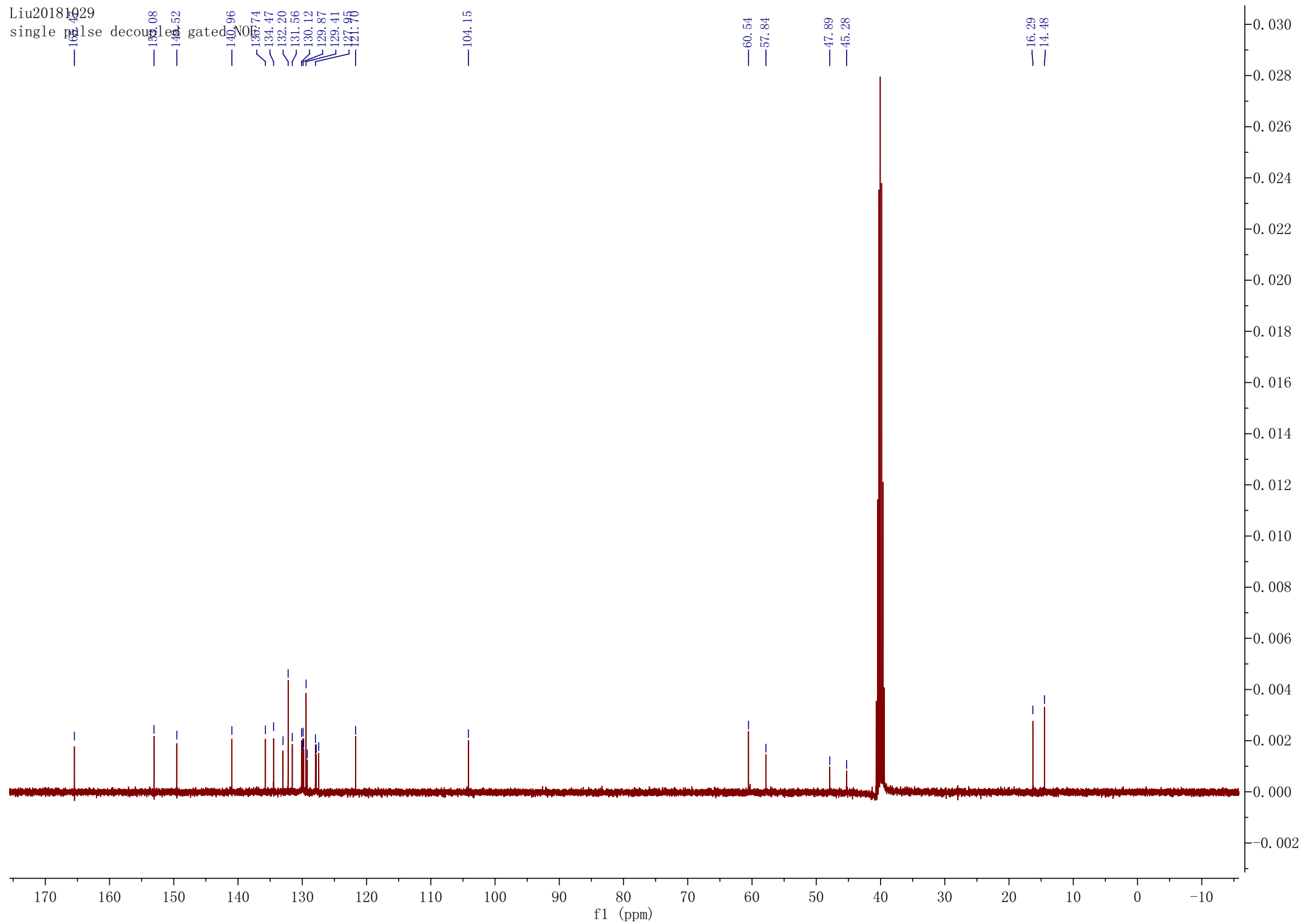

Supplement: Supplementary file 1 [file molecules-24-00891-s001.zip › molecules-433653-suppl/13C-NMR/N1 and N3 dialkylation of DHPMs.pdf]

LZL20160323  
Liu2016032301-1H

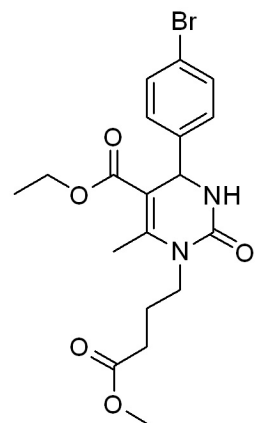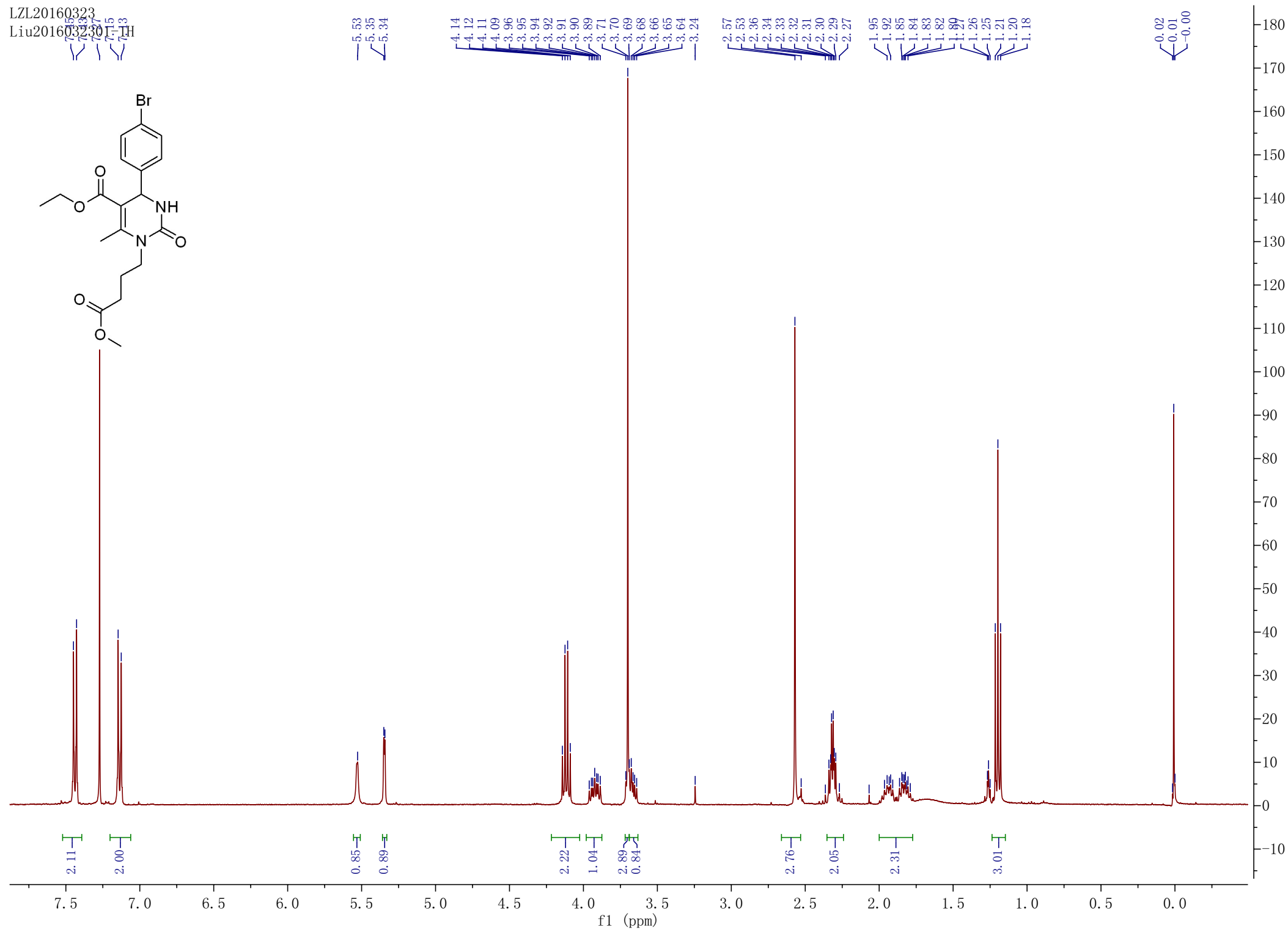

Supplement: Supplementary file 1 [file molecules-24-00891-s001.zip › molecules-433653-suppl/1H-NMR/1a.pdf]

LZL20160623  
Liu20160623-1H

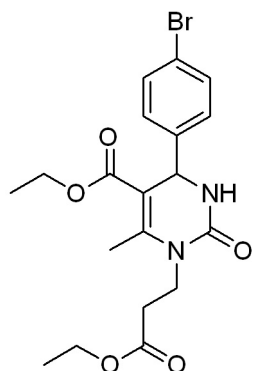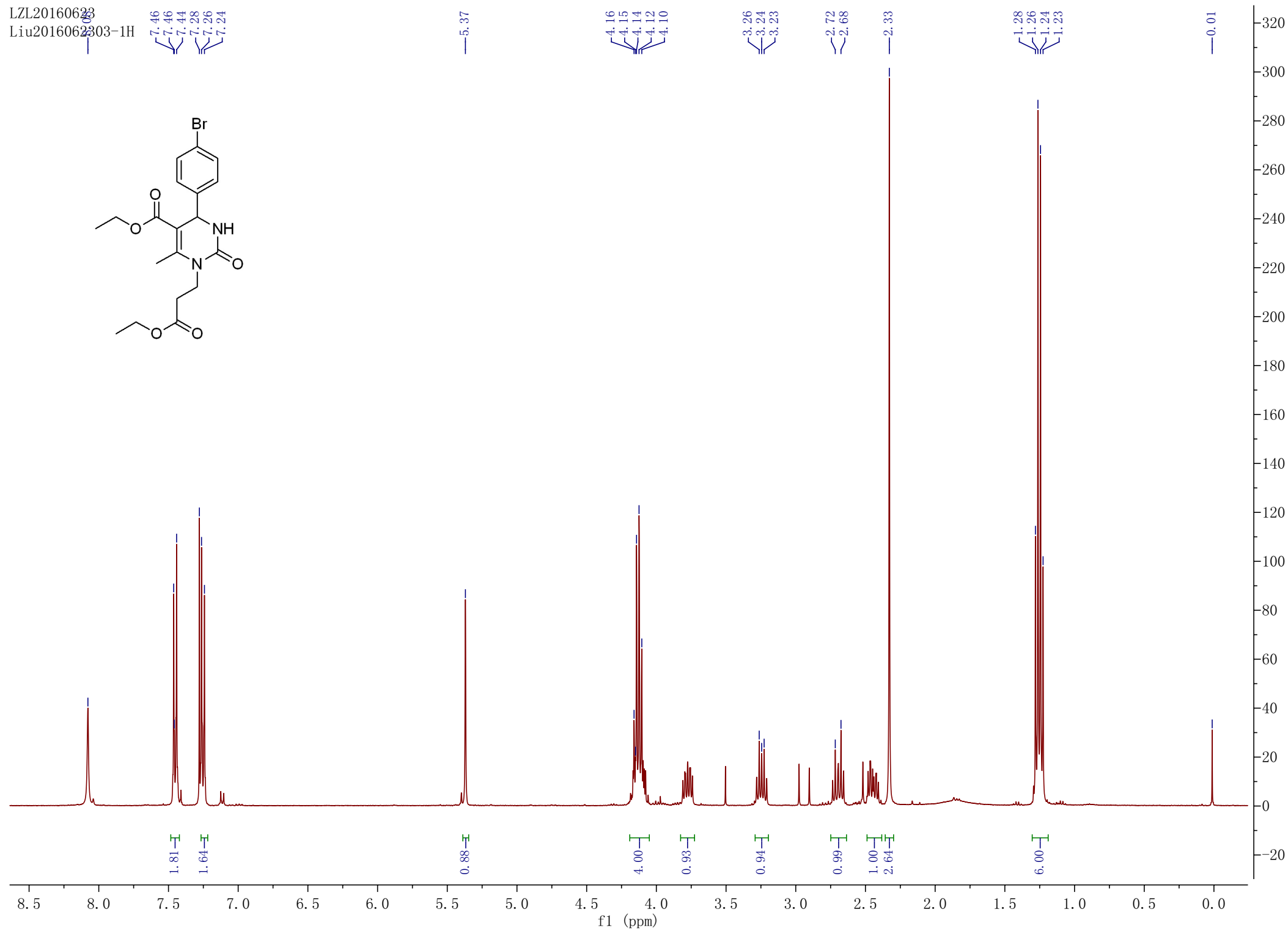

Supplement: Supplementary file 1 [file molecules-24-00891-s001.zip › molecules-433653-suppl/1H-NMR/1b.pdf]

LZL20160315  
Liu216031509-1H

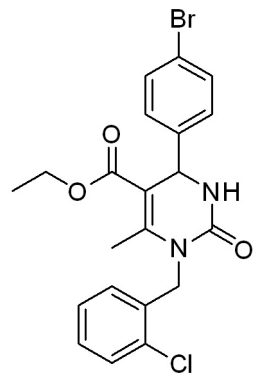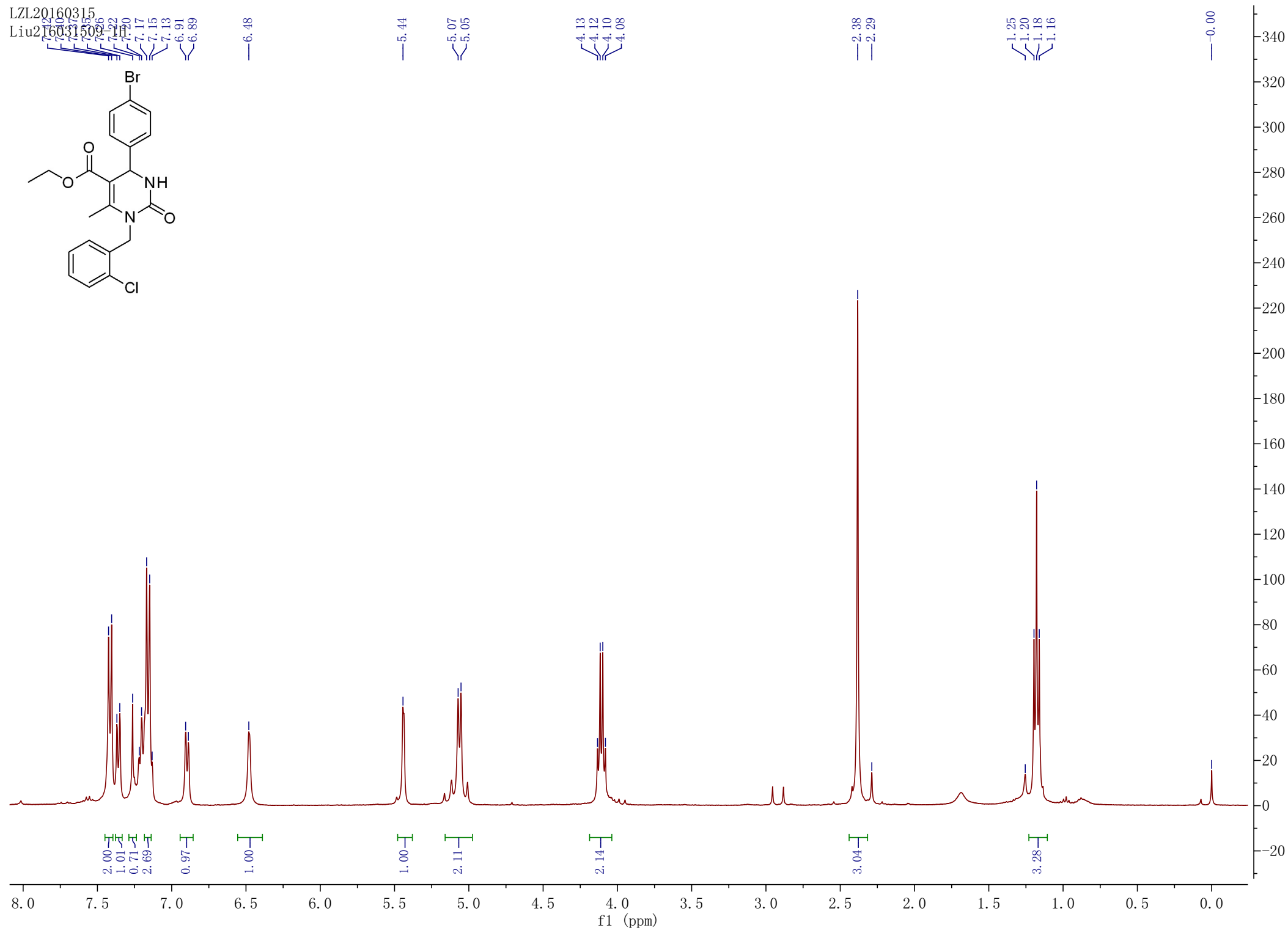

Supplement: Supplementary file 1 [file molecules-24-00891-s001.zip › molecules-433653-suppl/1H-NMR/1c.pdf]

LZL20160615  
Liu2016061502-1H

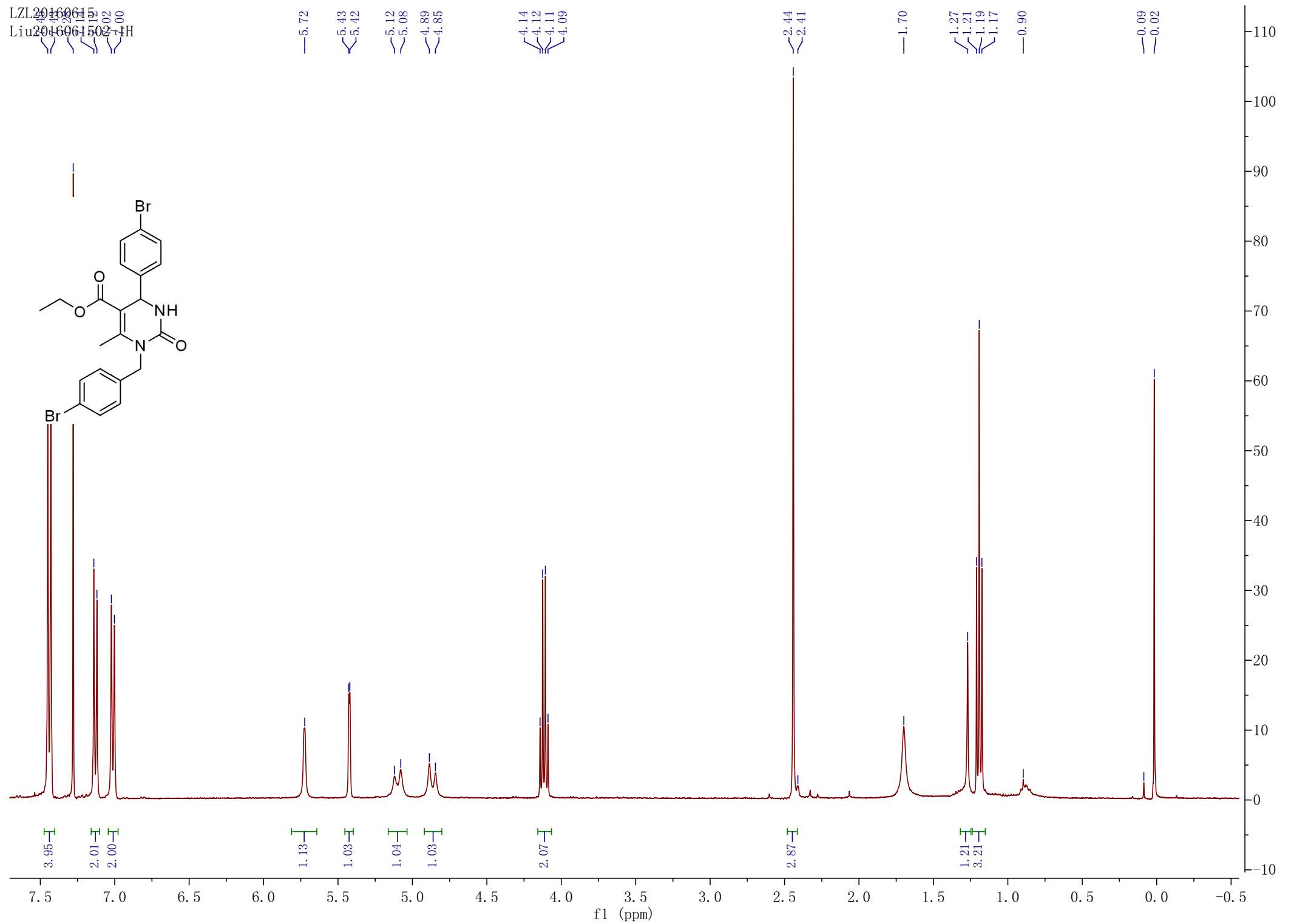

Supplement: Supplementary file 1 [file molecules-24-00891-s001.zip › molecules-433653-suppl/1H-NMR/1d.pdf]

LZ120160617  
Liu2016061702-1H

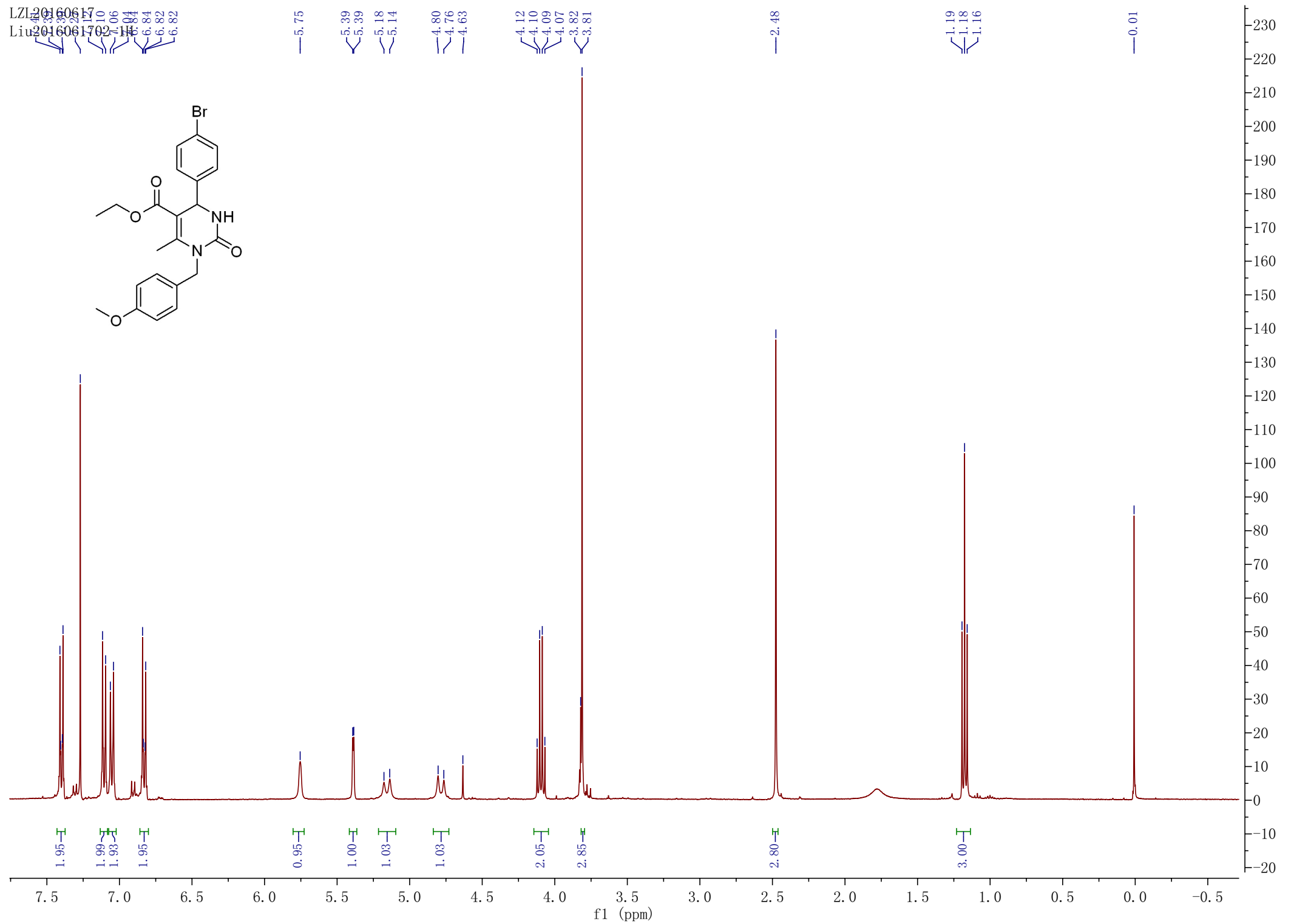

Supplement: Supplementary file 1 [file molecules-24-00891-s001.zip › molecules-433653-suppl/1H-NMR/1e.pdf]

LZL20160323  
Liu2016032302-H

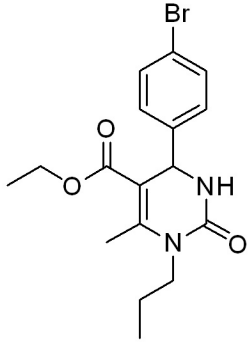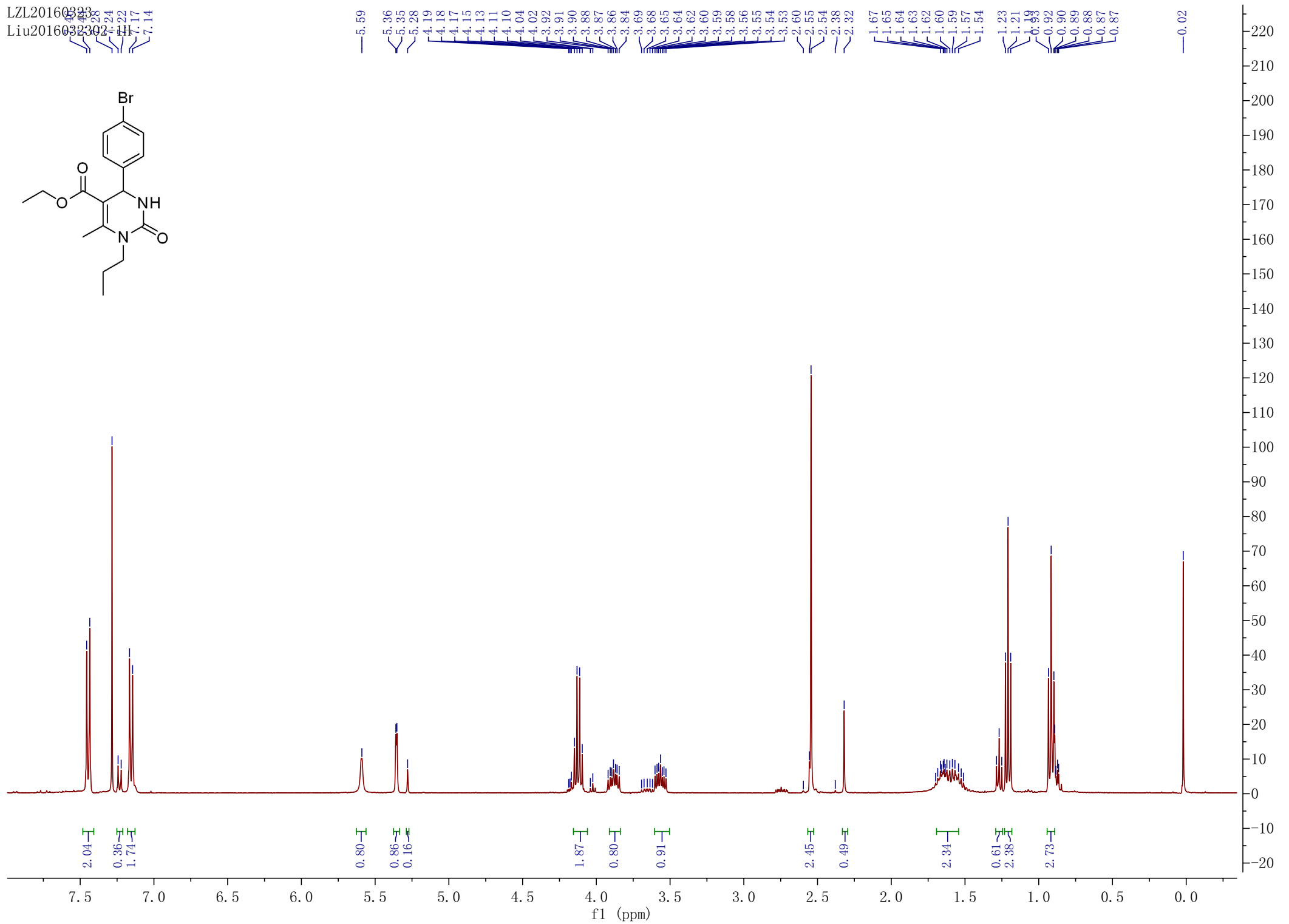

Supplement: Supplementary file 1 [file molecules-24-00891-s001.zip › molecules-433653-suppl/1H-NMR/1f.pdf]

LZL20160425  
Liu2016042503-1H

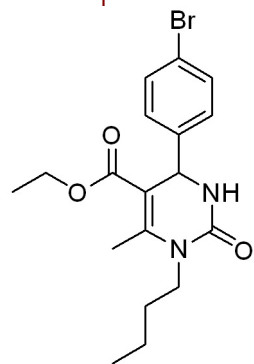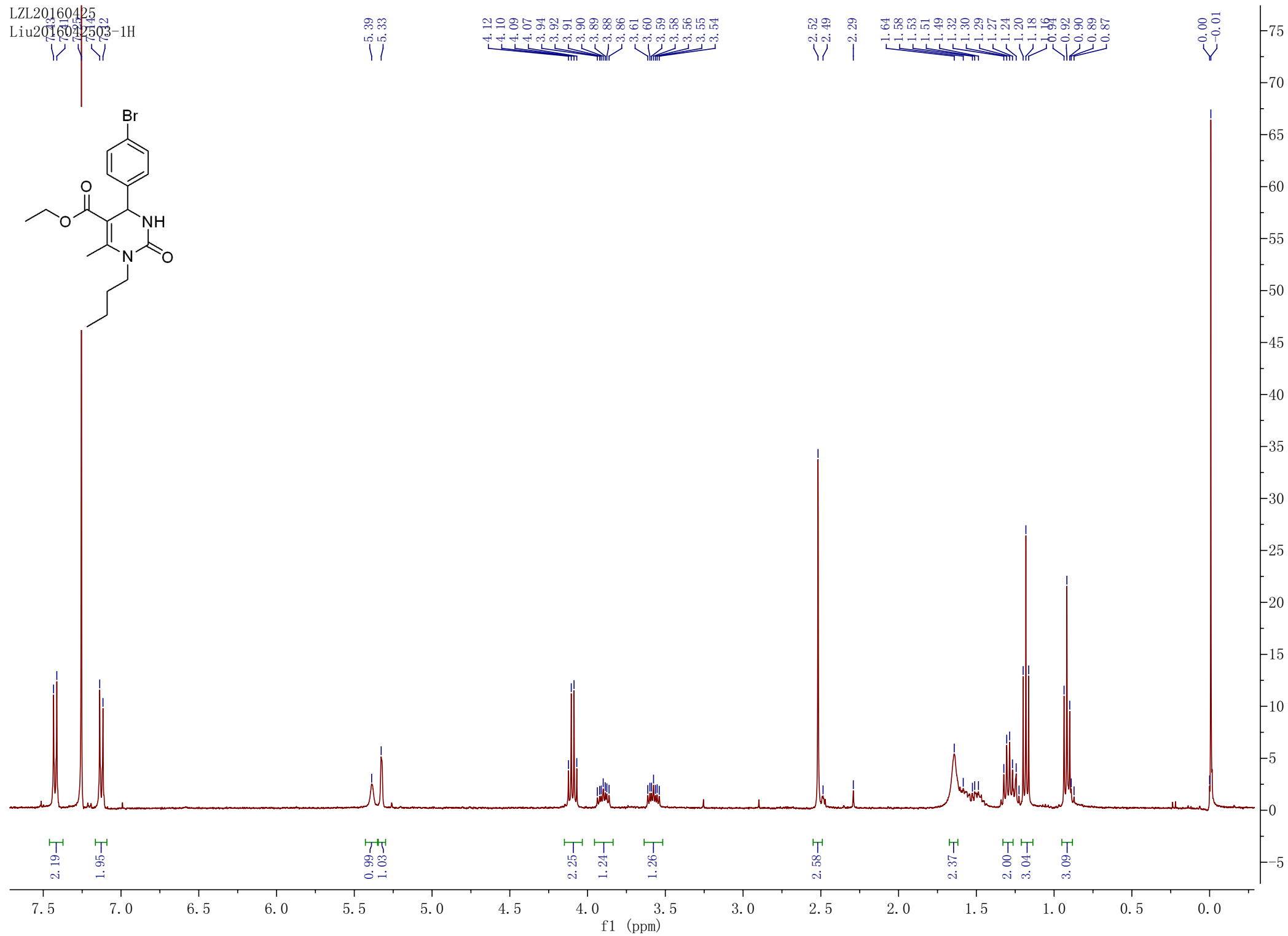

Supplement: Supplementary file 1 [file molecules-24-00891-s001.zip › molecules-433653-suppl/1H-NMR/1g.pdf]

LZL20161018  
Liu2016101802-11

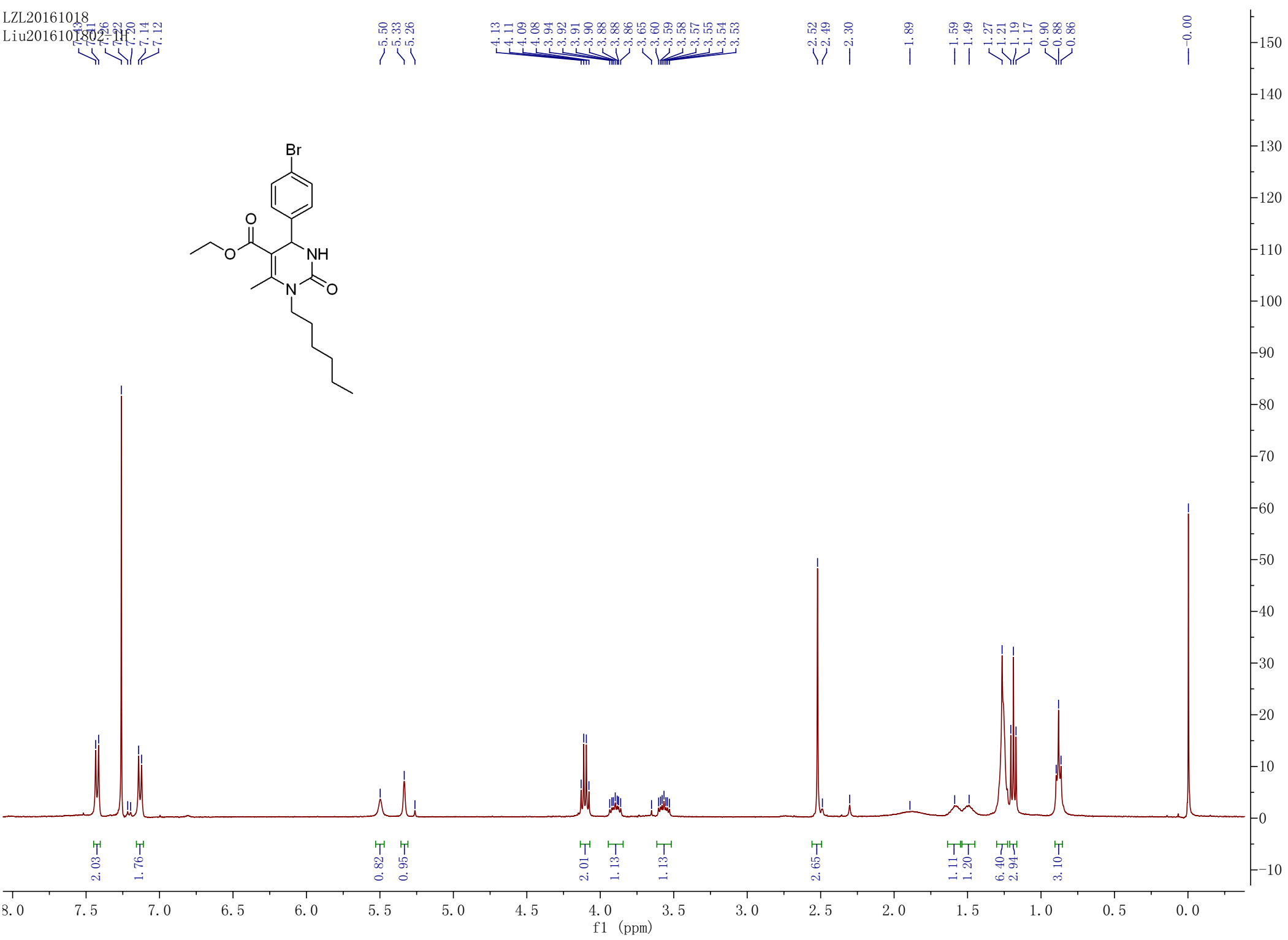

Supplement: Supplementary file 1 [file molecules-24-00891-s001.zip › molecules-433653-suppl/1H-NMR/1h.pdf]

LZL20170702

Liu2017070202-11

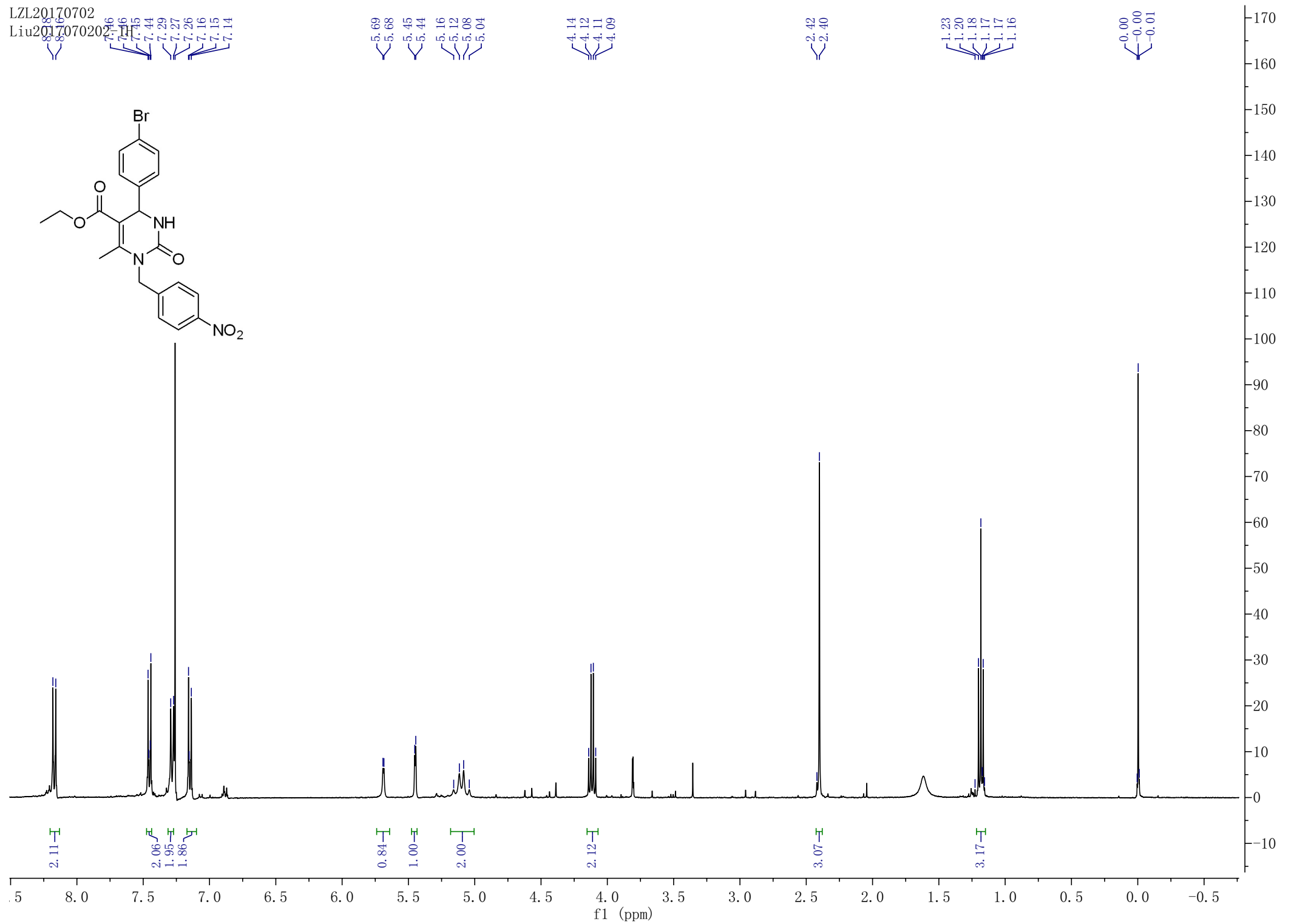

Supplement: Supplementary file 1 [file molecules-24-00891-s001.zip › molecules-433653-suppl/1H-NMR/1j.pdf]

LZL20161009  
Liu2016100804-1

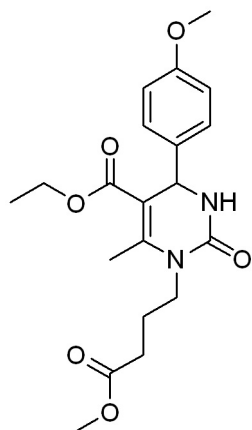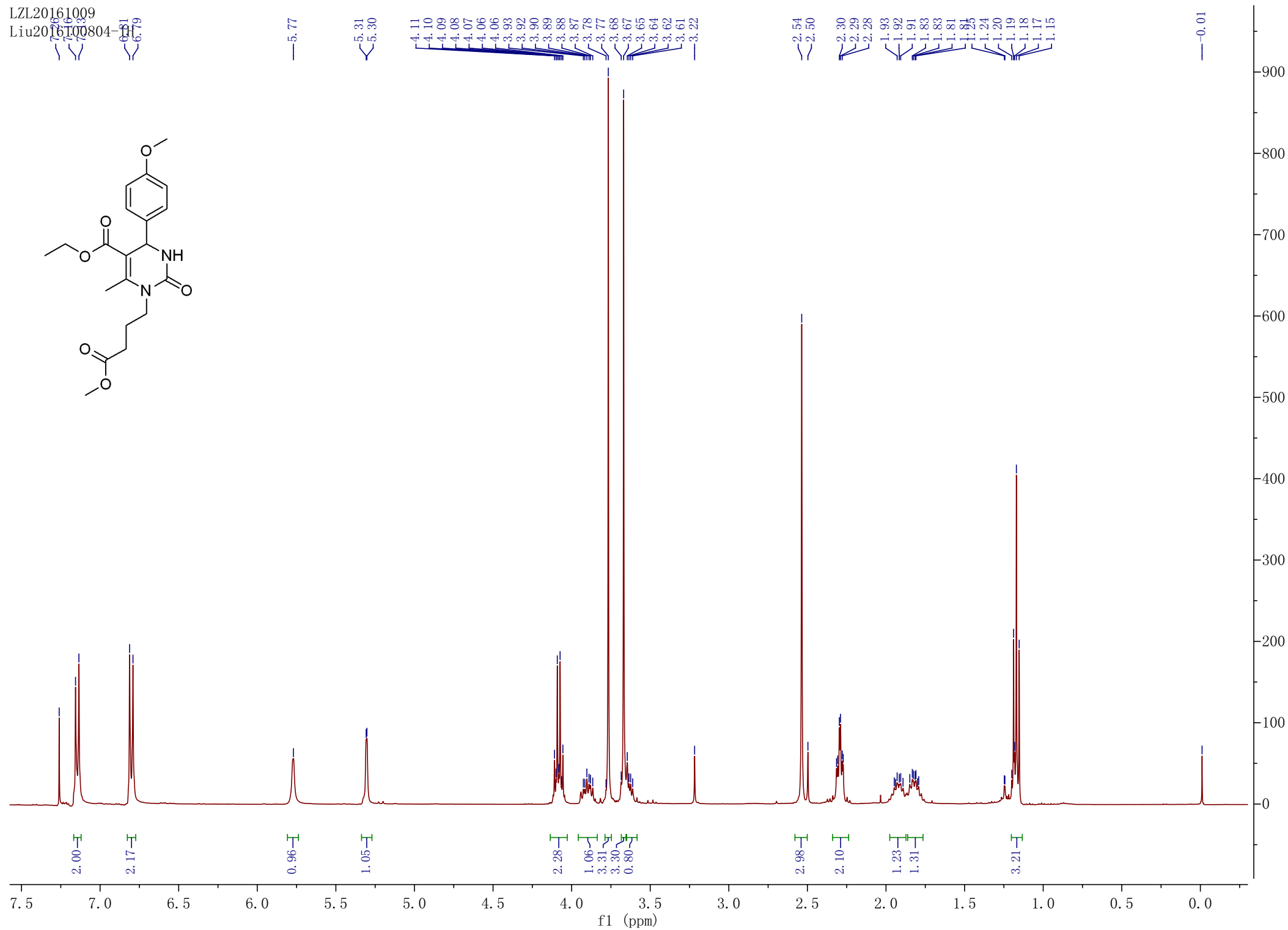

Supplement: Supplementary file 1 [file molecules-24-00891-s001.zip › molecules-433653-suppl/1H-NMR/2a.pdf]

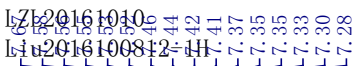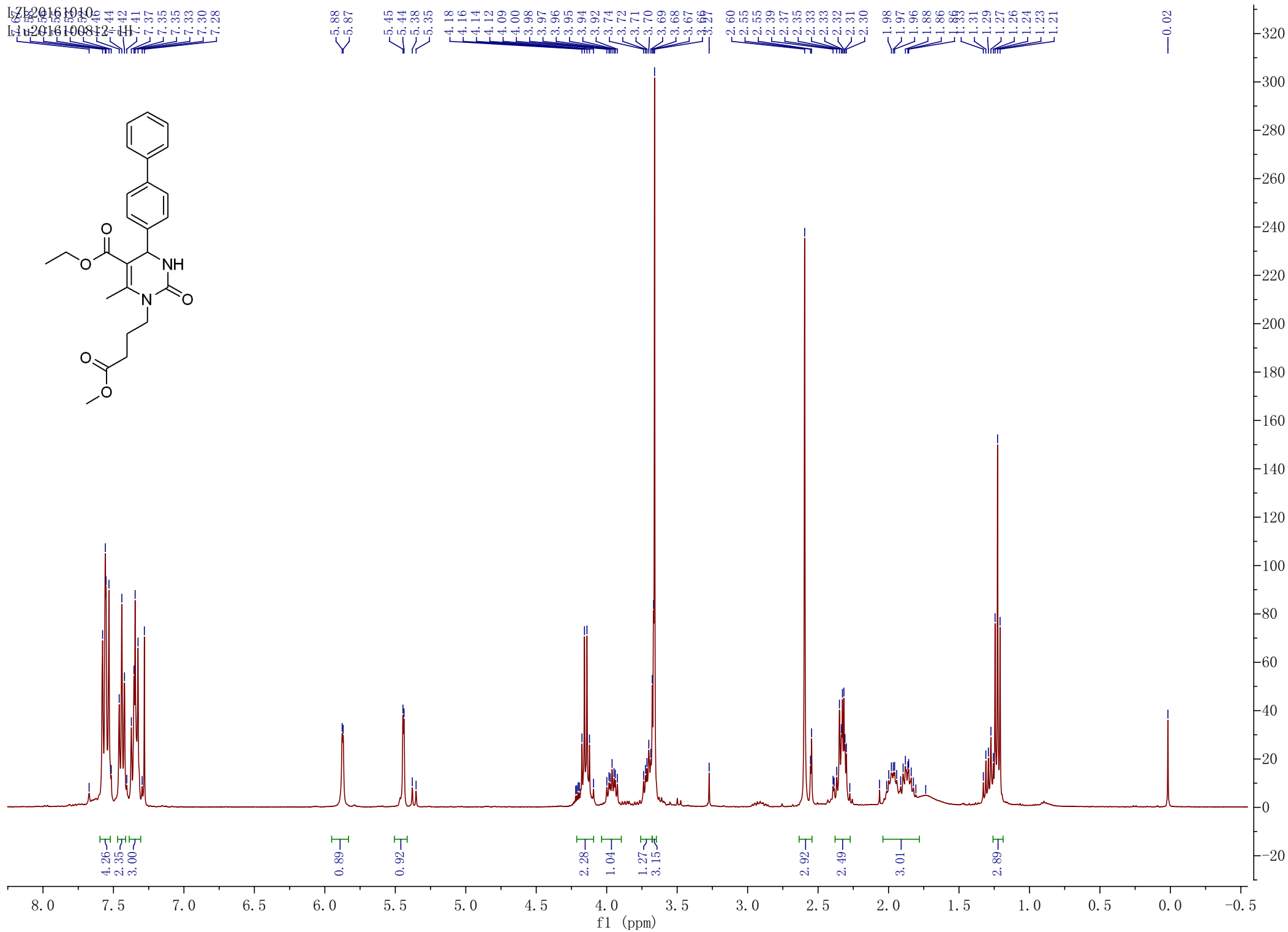

Supplement: Supplementary file 1 [file molecules-24-00891-s001.zip › molecules-433653-suppl/1H-NMR/3a.pdf]

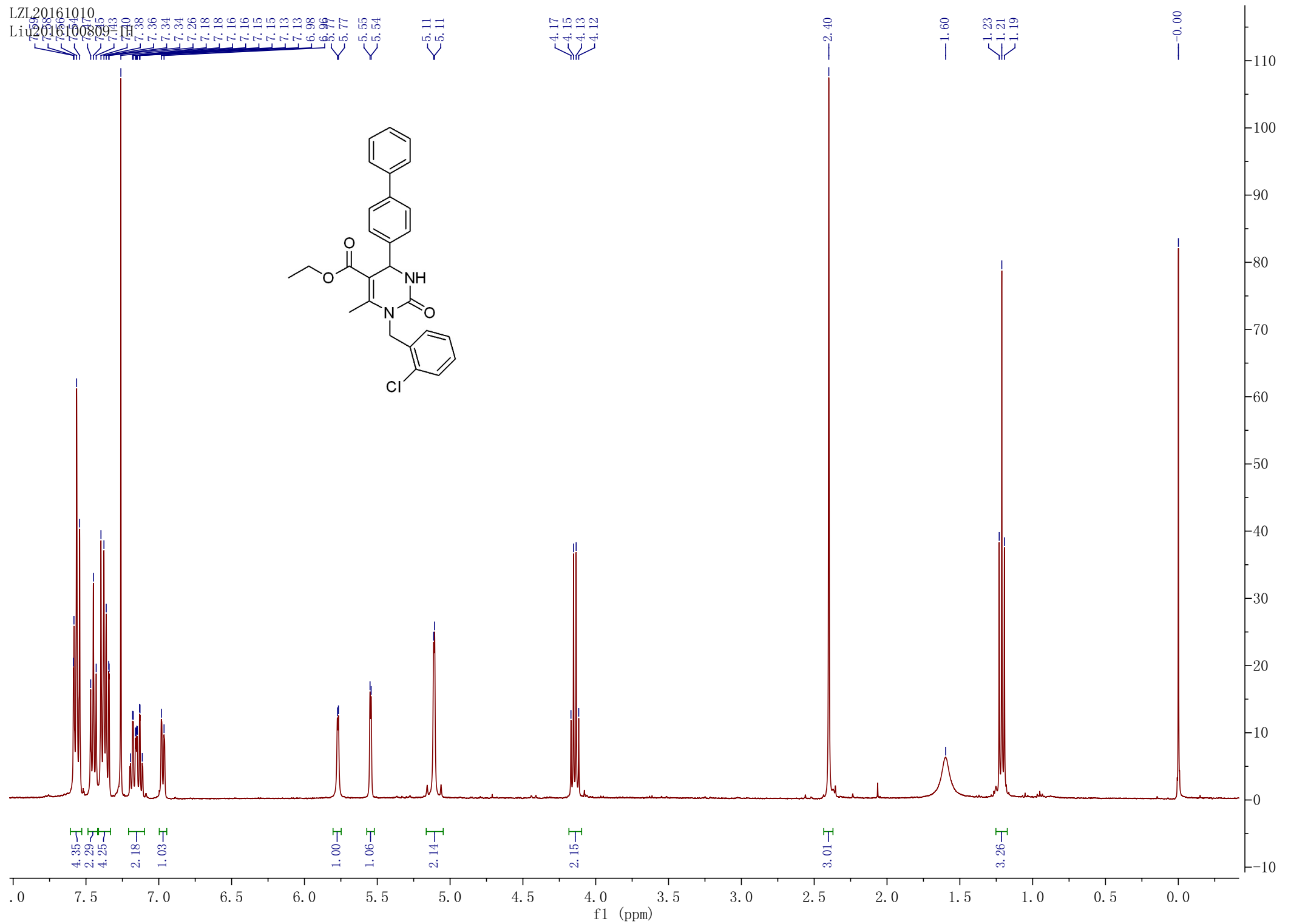

Supplement: Supplementary file 1 [file molecules-24-00891-s001.zip › molecules-433653-suppl/1H-NMR/3c.pdf]

LZ120160623  
Liu2016062304-11

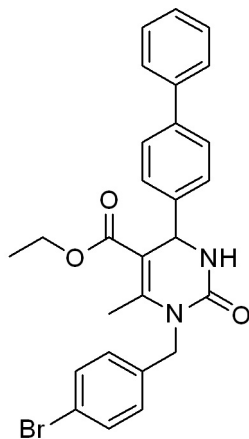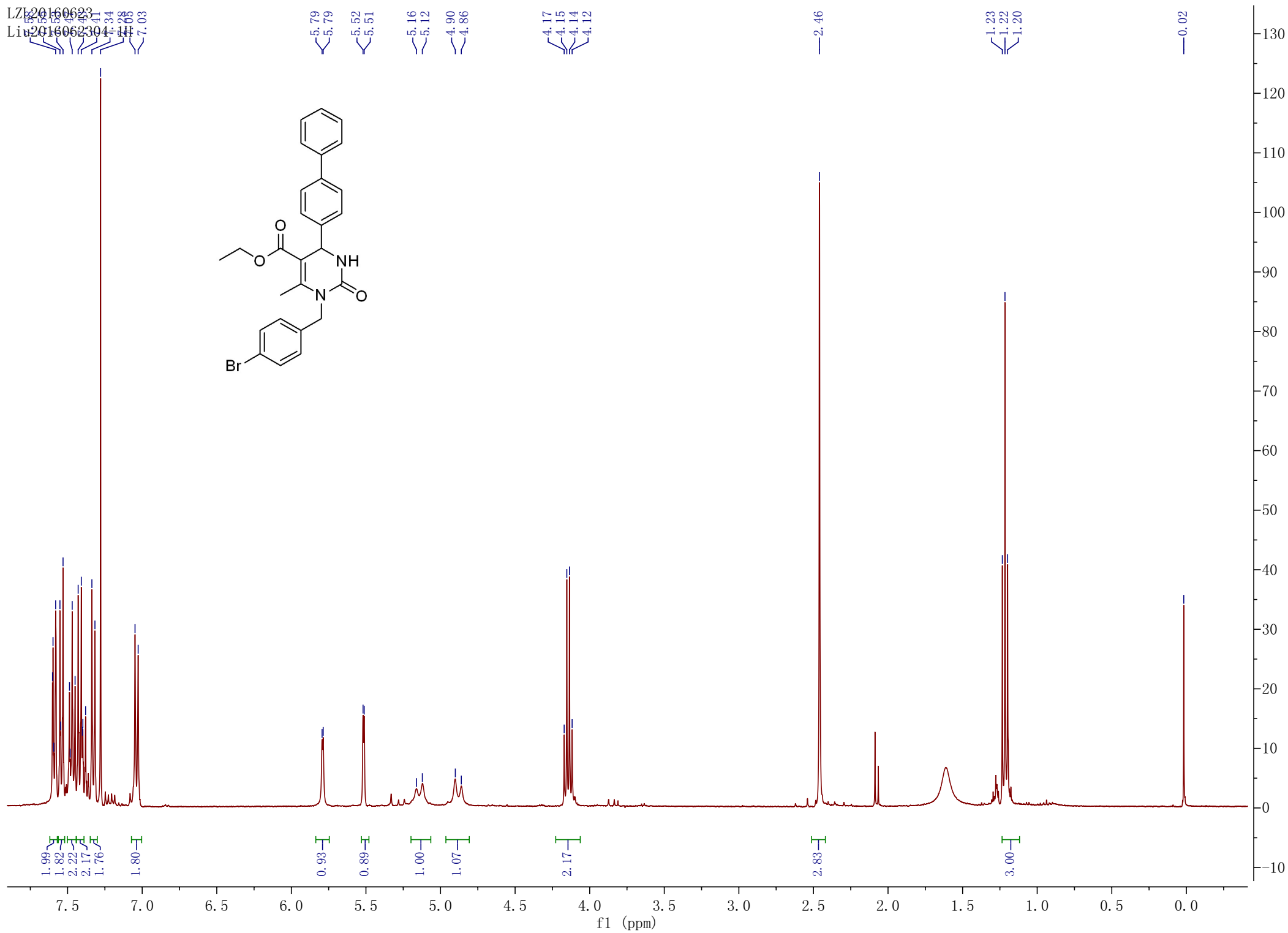

Supplement: Supplementary file 1 [file molecules-24-00891-s001.zip › molecules-433653-suppl/1H-NMR/3d.pdf]

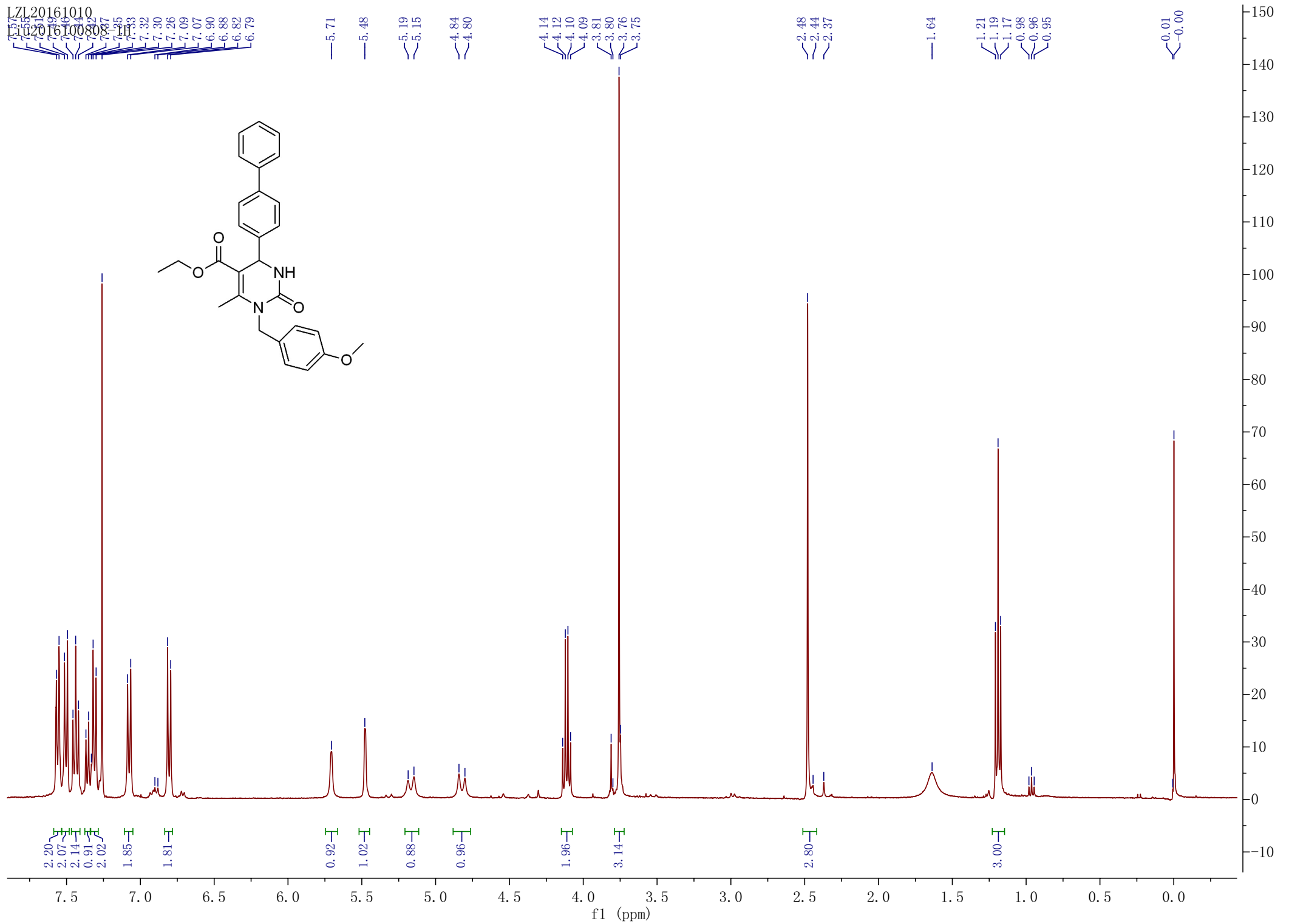

Supplement: Supplementary file 1 [file molecules-24-00891-s001.zip › molecules-433653-suppl/1H-NMR/3e.pdf]

LZL20161010  
Fig2016100810-1H

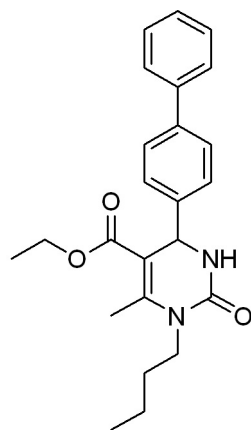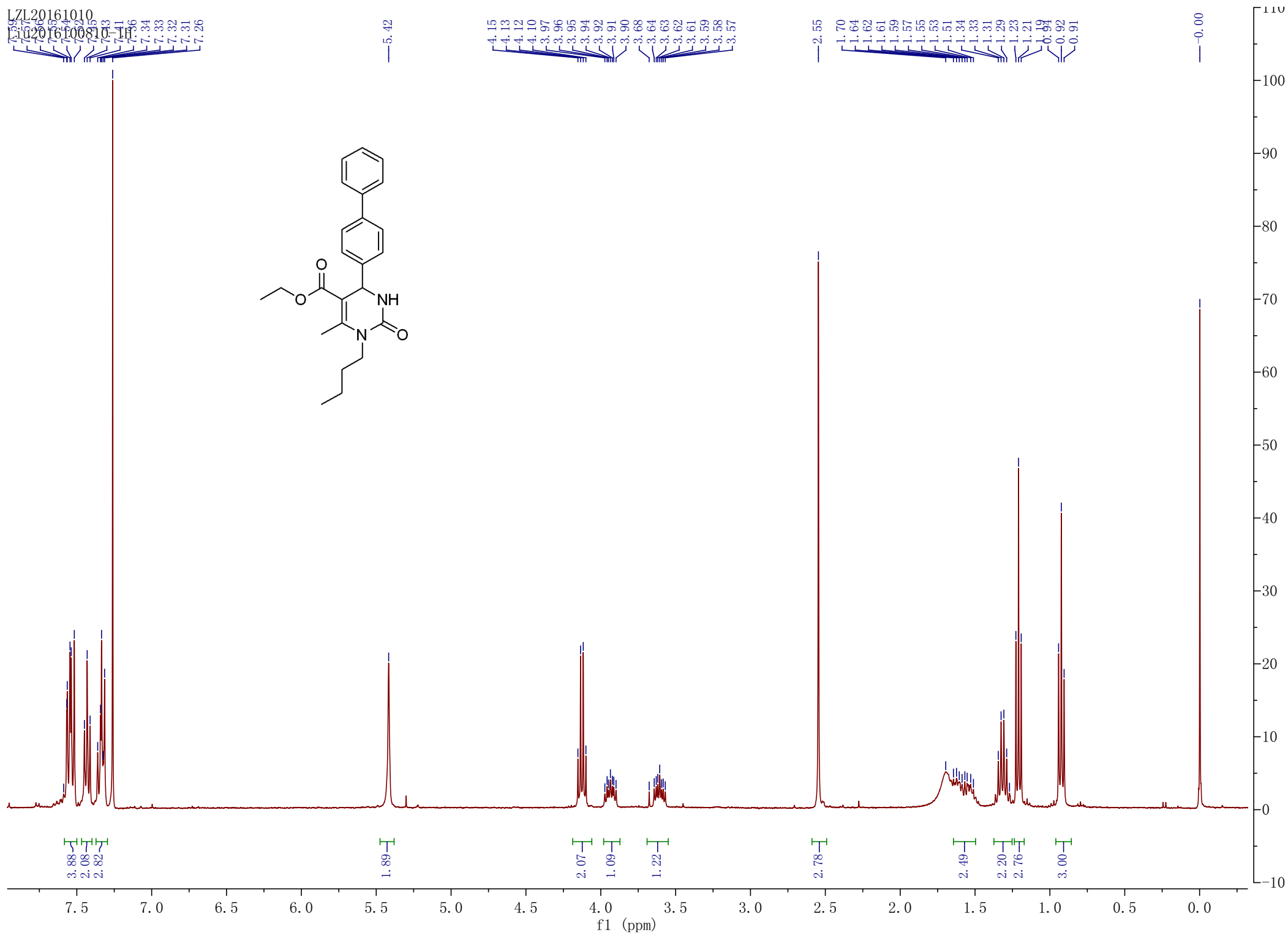

Supplement: Supplementary file 1 [file molecules-24-00891-s001.zip › molecules-433653-suppl/1H-NMR/3g.pdf]

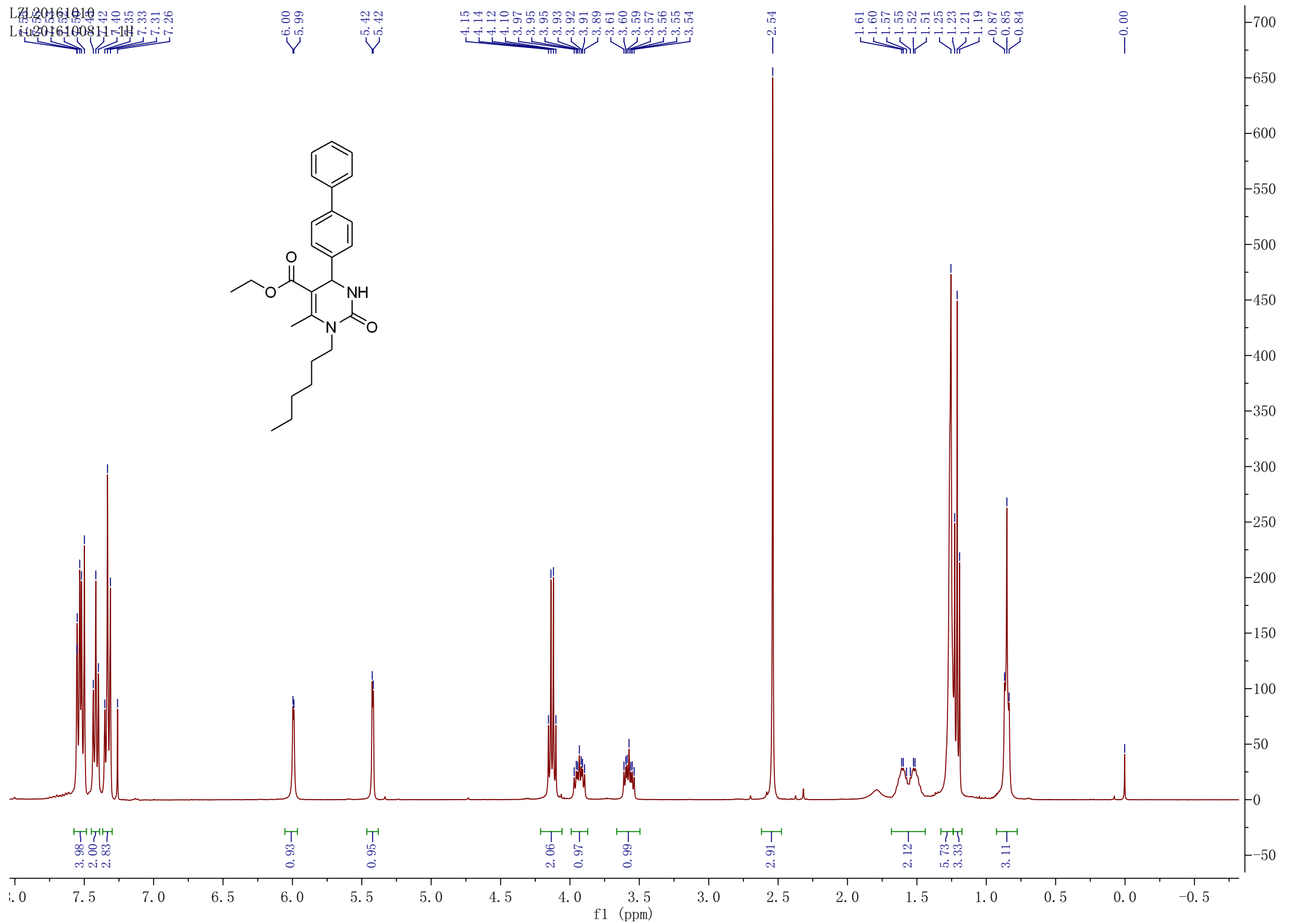

Supplement: Supplementary file 1 [file molecules-24-00891-s001.zip › molecules-433653-suppl/1H-NMR/3h.pdf]

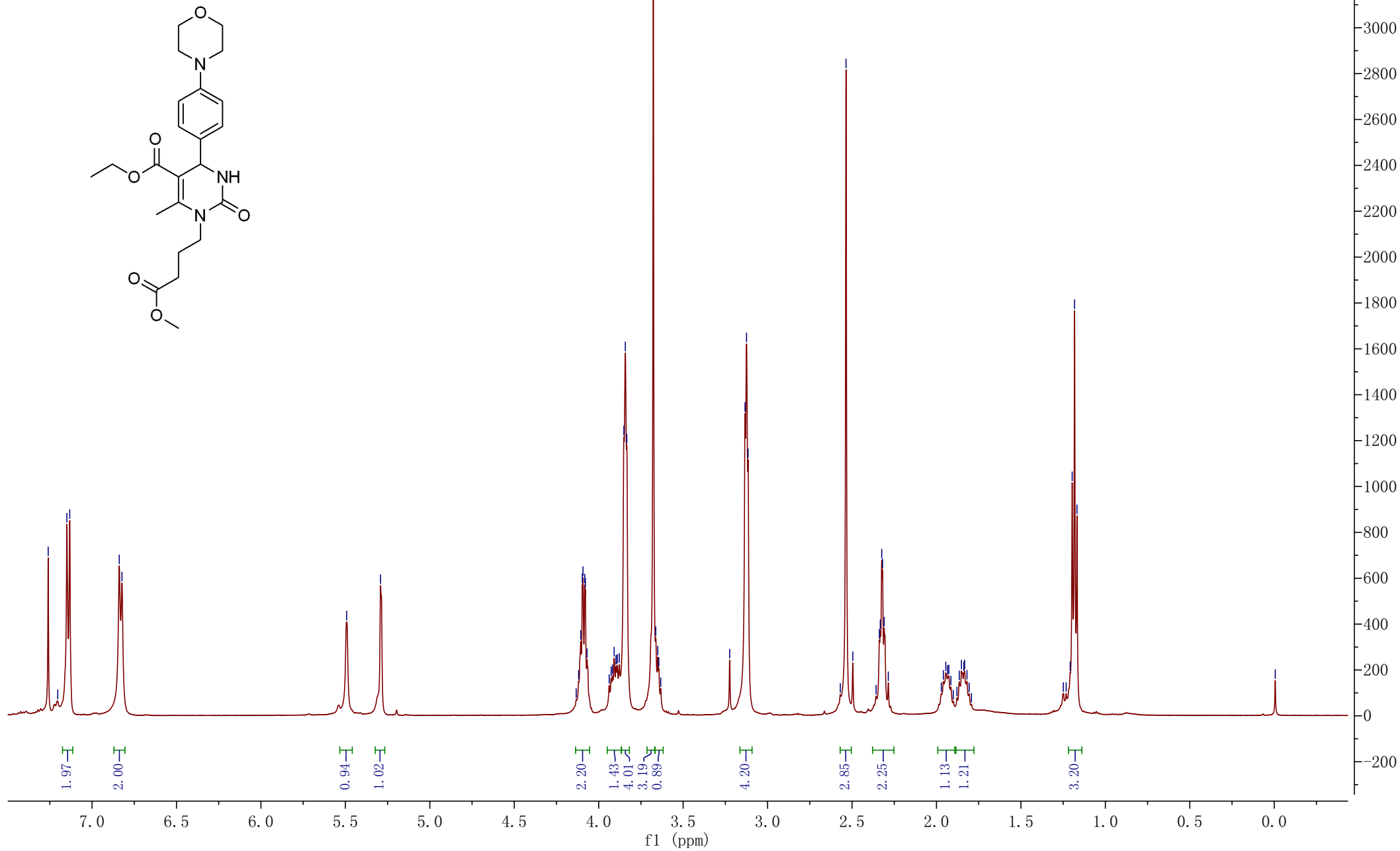

Supplement: Supplementary file 1 [file molecules-24-00891-s001.zip › molecules-433653-suppl/1H-NMR/4a.pdf]

LZL  
Liu2017040502-1H

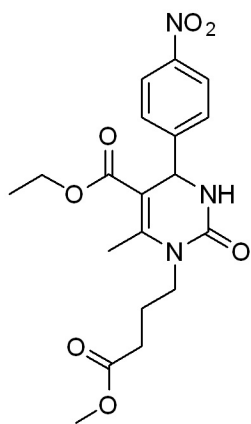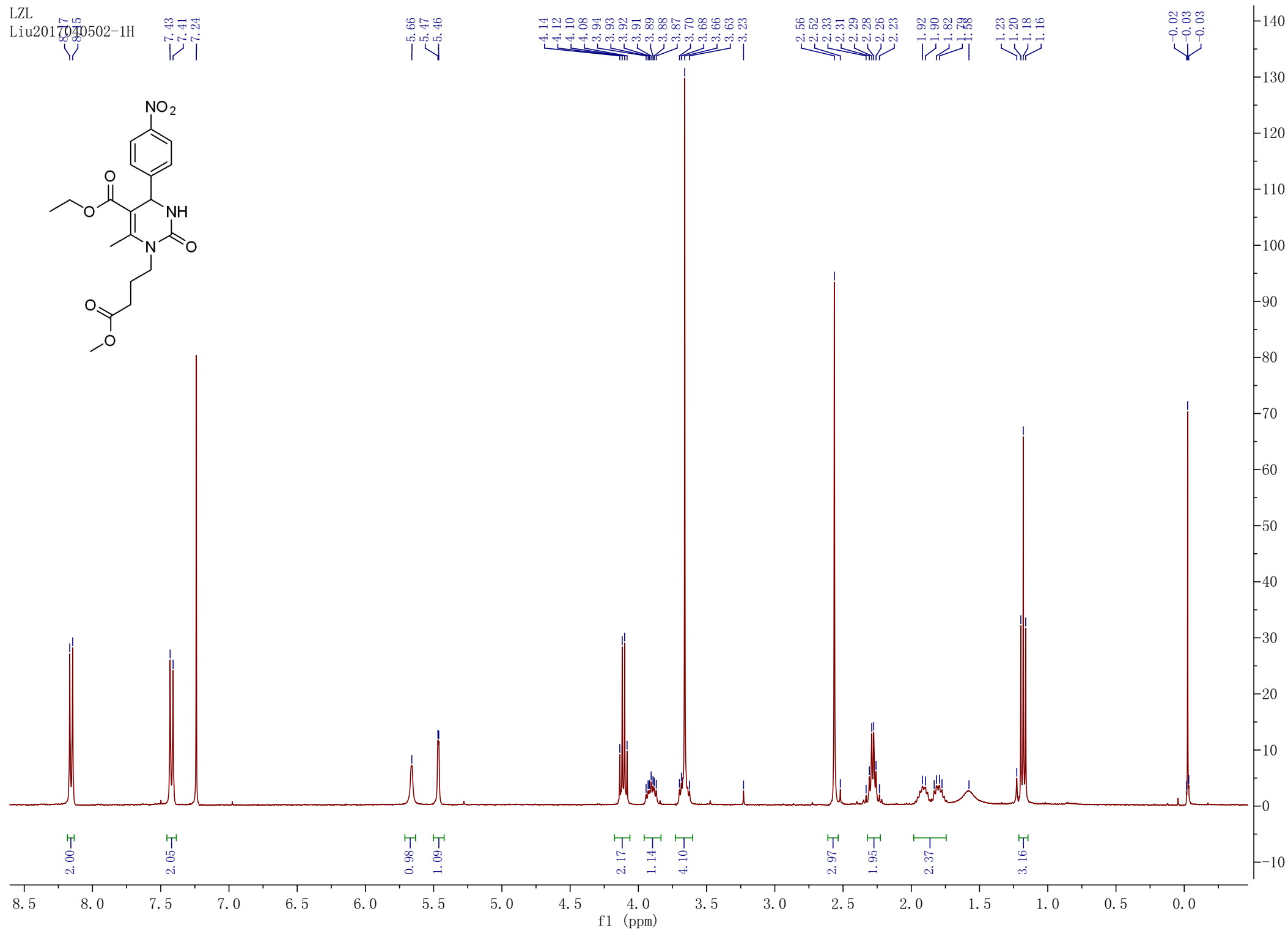

Supplement: Supplementary file 1 [file molecules-24-00891-s001.zip › molecules-433653-suppl/1H-NMR/5a.pdf]

LZL20170523  
LZL2017052301-1H

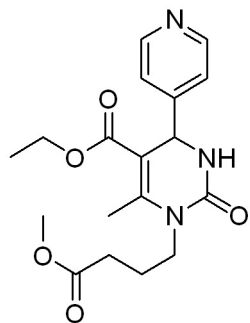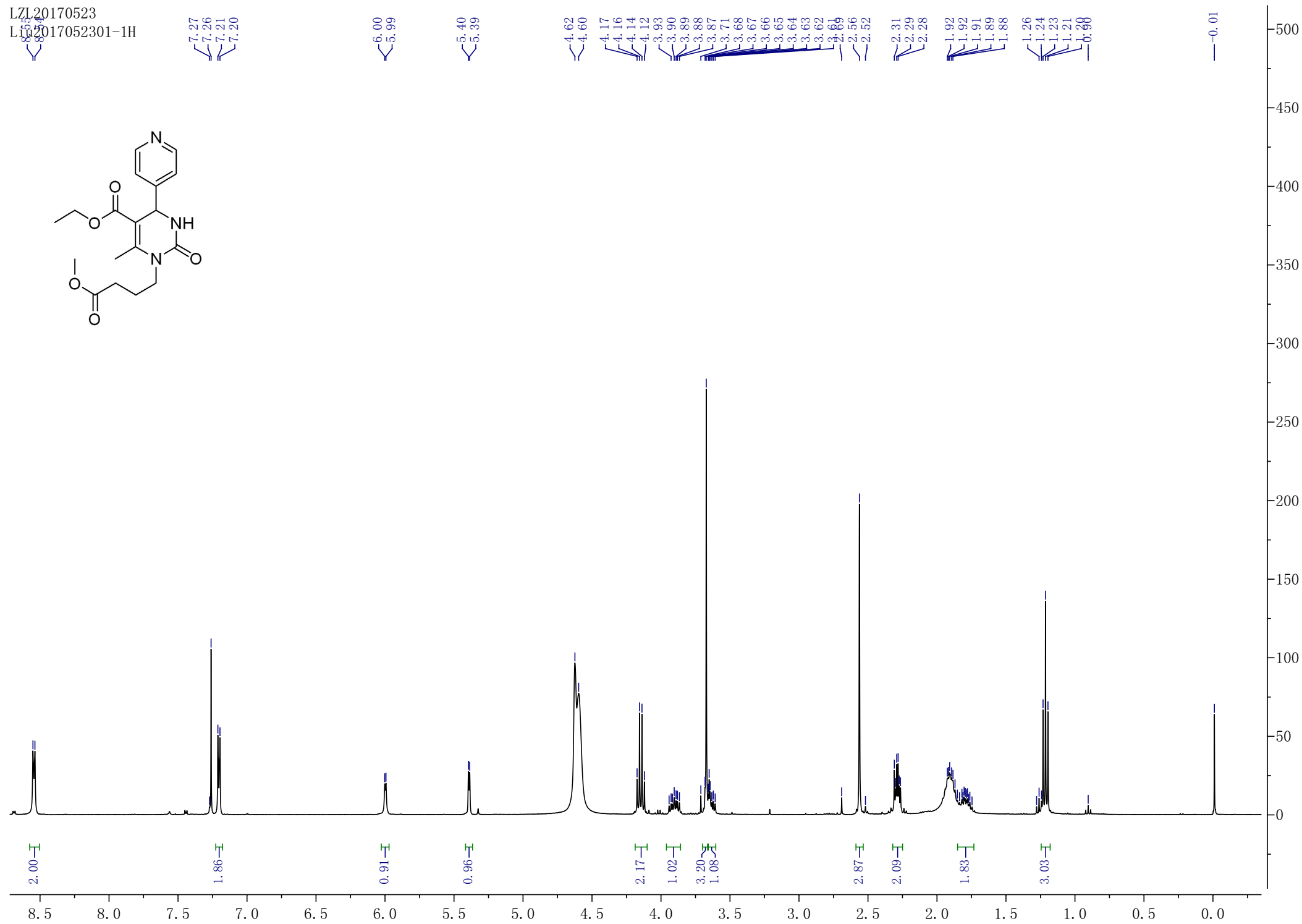

Supplement: Supplementary file 1 [file molecules-24-00891-s001.zip › molecules-433653-suppl/1H-NMR/6a.pdf]

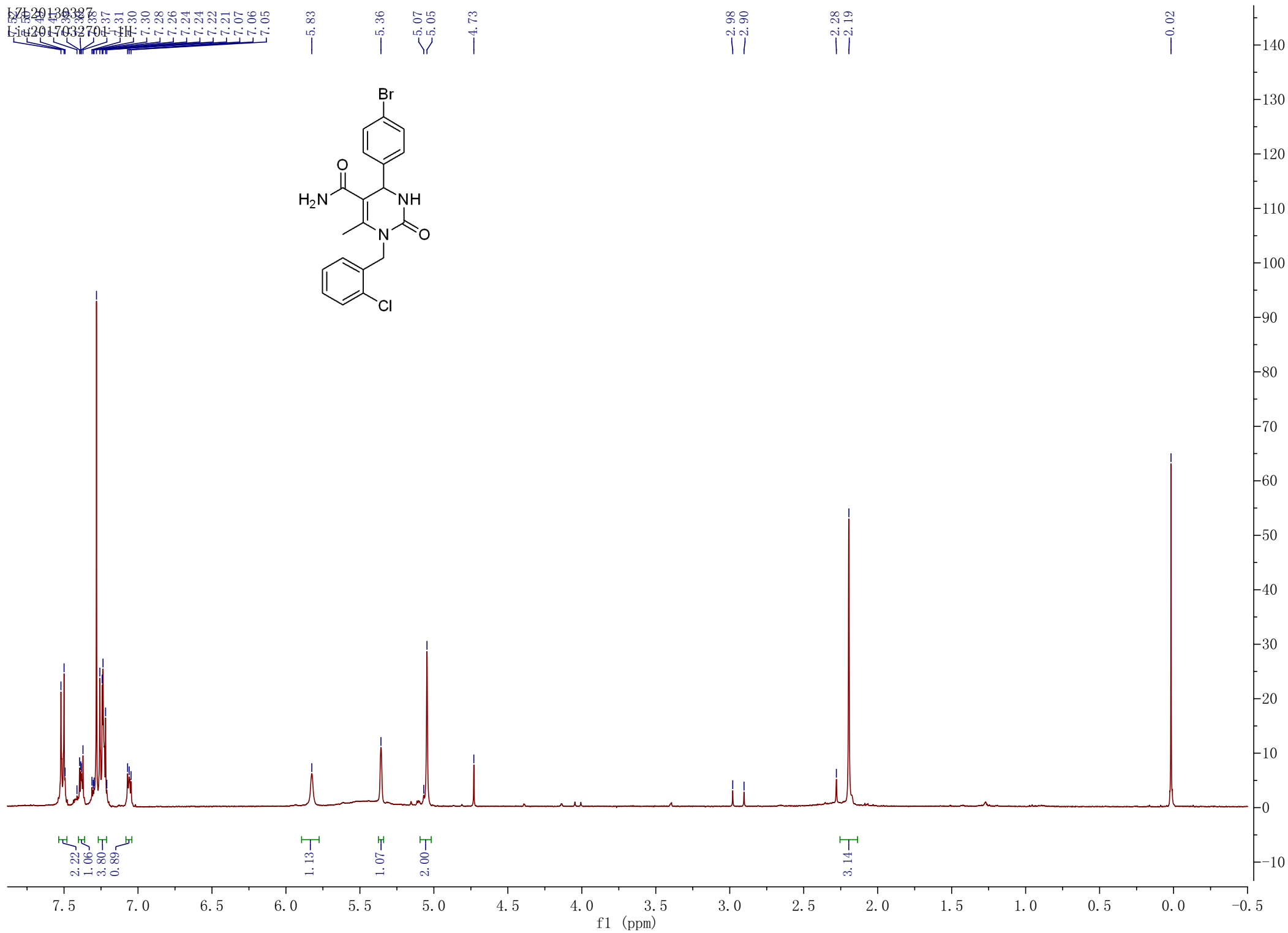

Supplement: Supplementary file 1 [file molecules-24-00891-s001.zip › molecules-433653-suppl/1H-NMR/7c.pdf]

LZL20170419  
Liu2017041903-1H

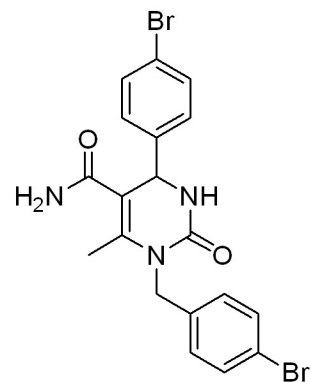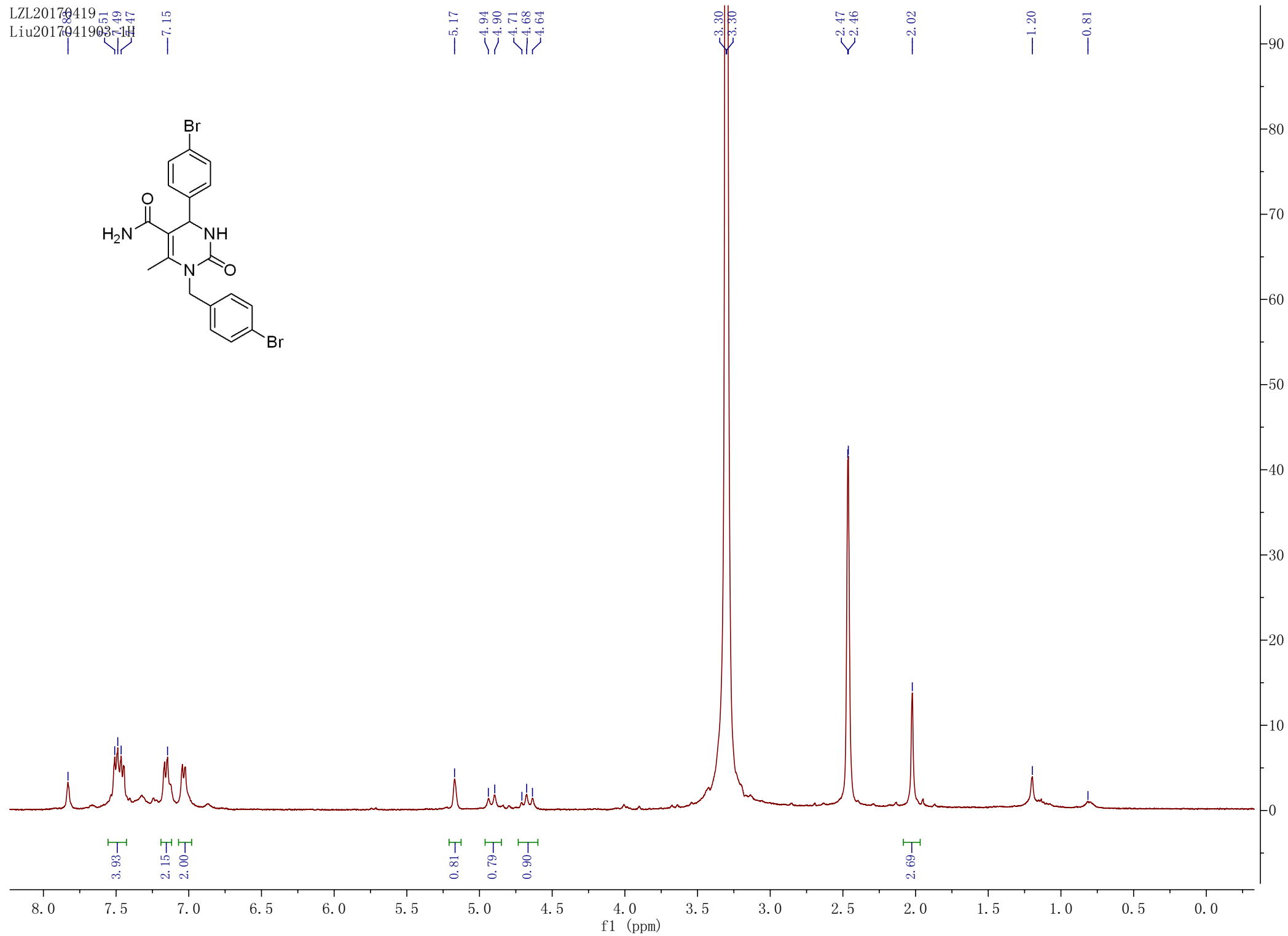

Supplement: Supplementary file 1 [file molecules-24-00891-s001.zip › molecules-433653-suppl/1H-NMR/7d.pdf]

LZL20170515  
Liu2017051504-1H

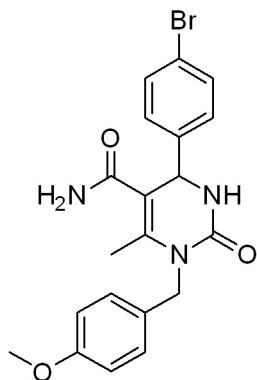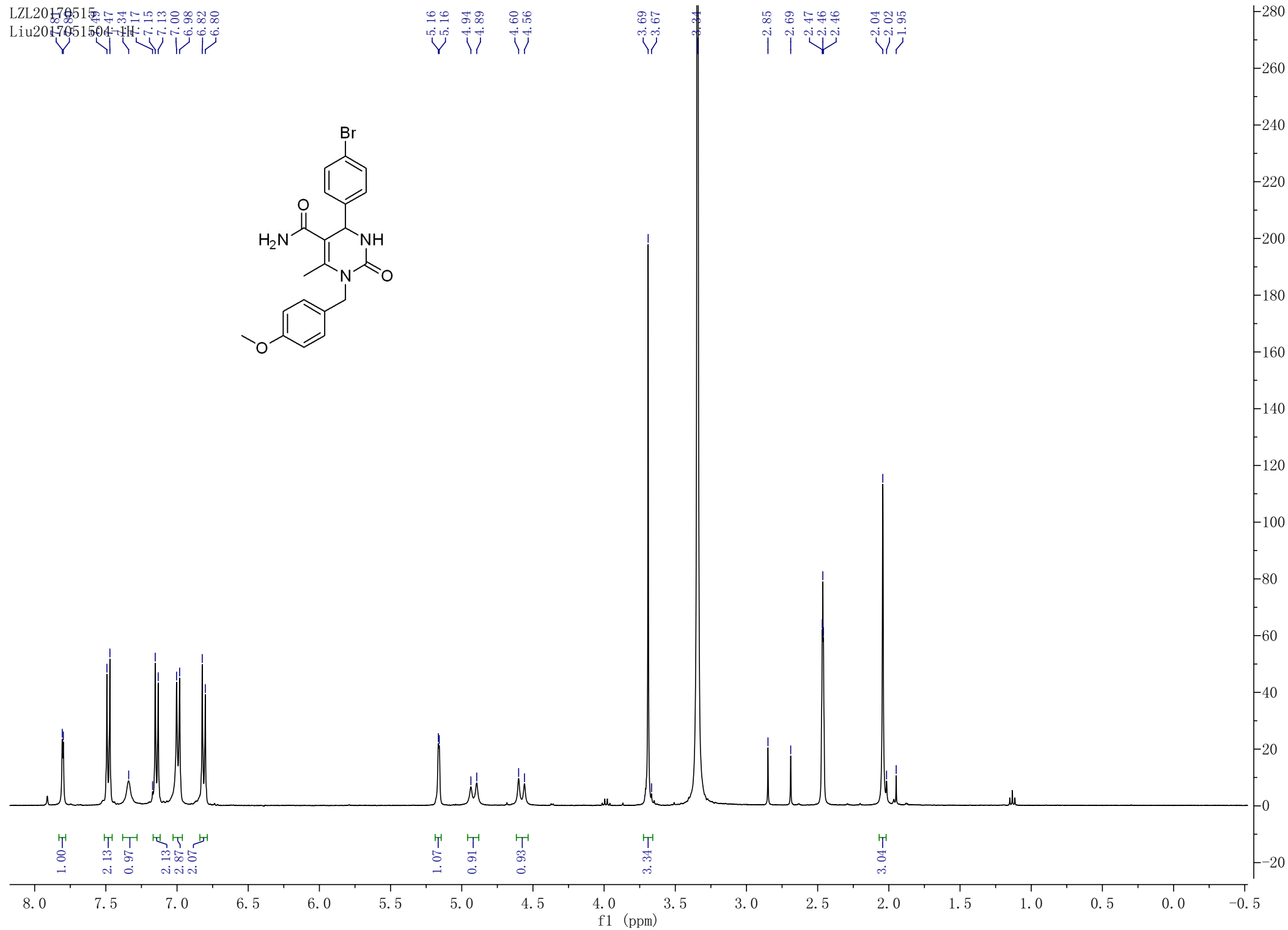

Supplement: Supplementary file 1 [file molecules-24-00891-s001.zip › molecules-433653-suppl/1H-NMR/7e.pdf]

氢谱  
LZ-704-1H

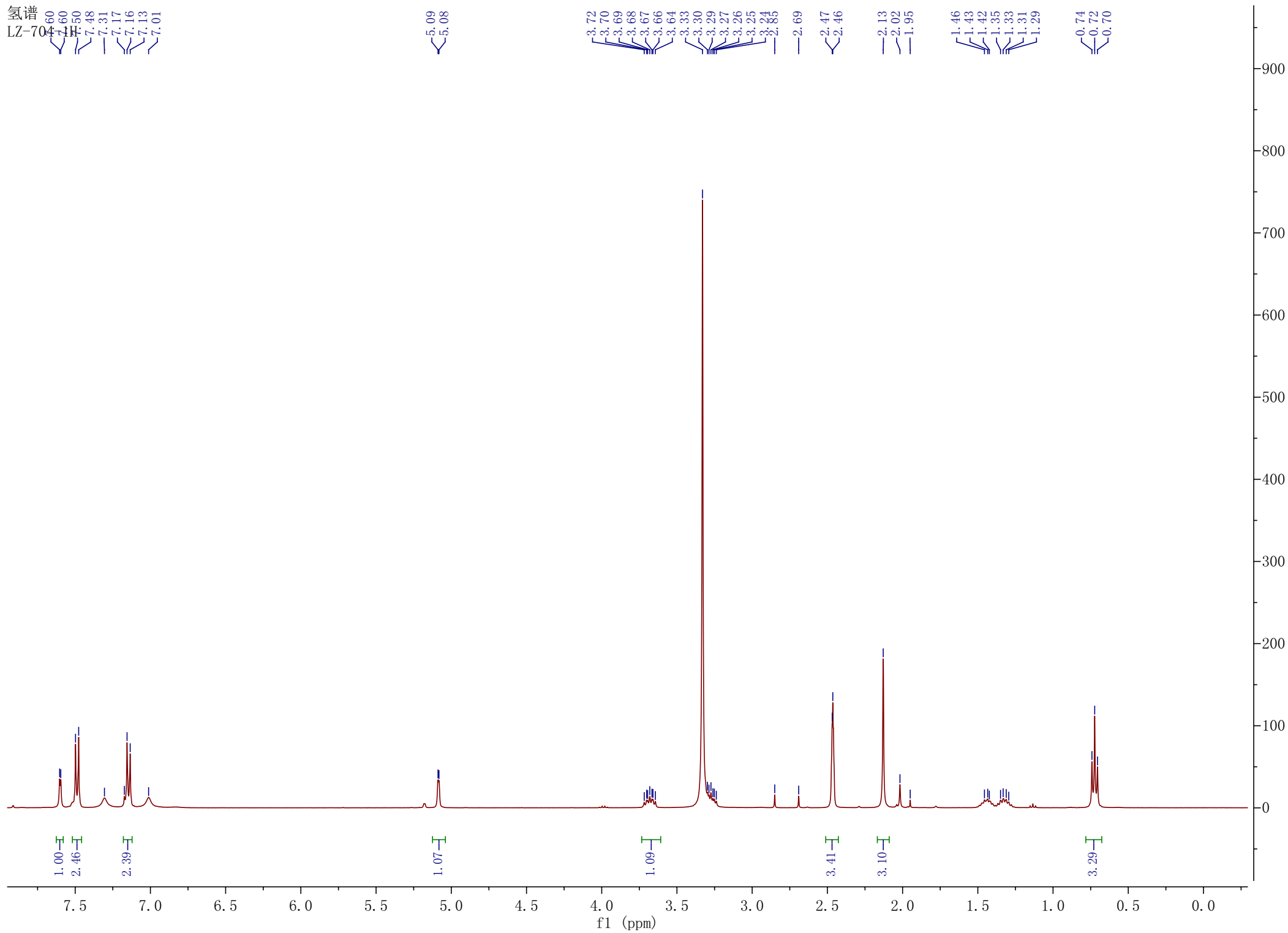

Supplement: Supplementary file 1 [file molecules-24-00891-s001.zip › molecules-433653-suppl/1H-NMR/7f.pdf]

LZL20170619  
Liu17061903-1H

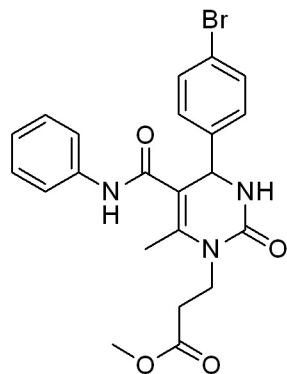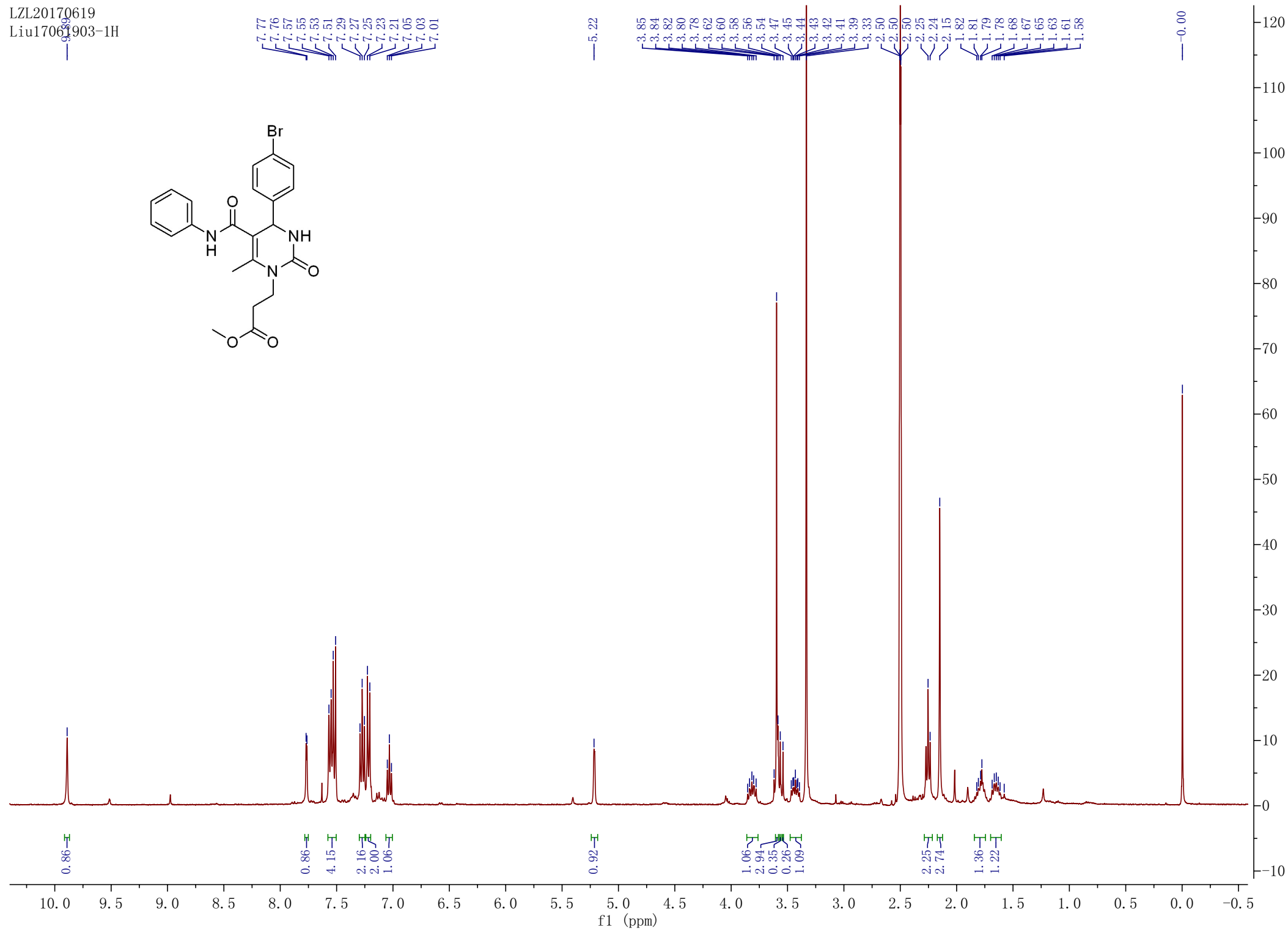

Supplement: Supplementary file 1 [file molecules-24-00891-s001.zip › molecules-433653-suppl/1H-NMR/8a.pdf]

LZL20170608  
Liub17060801-1H

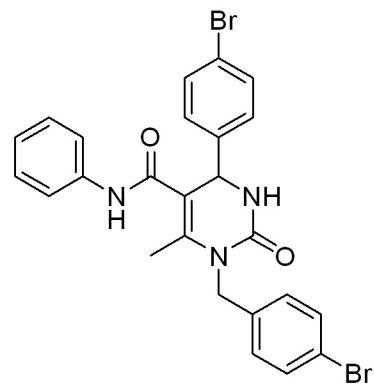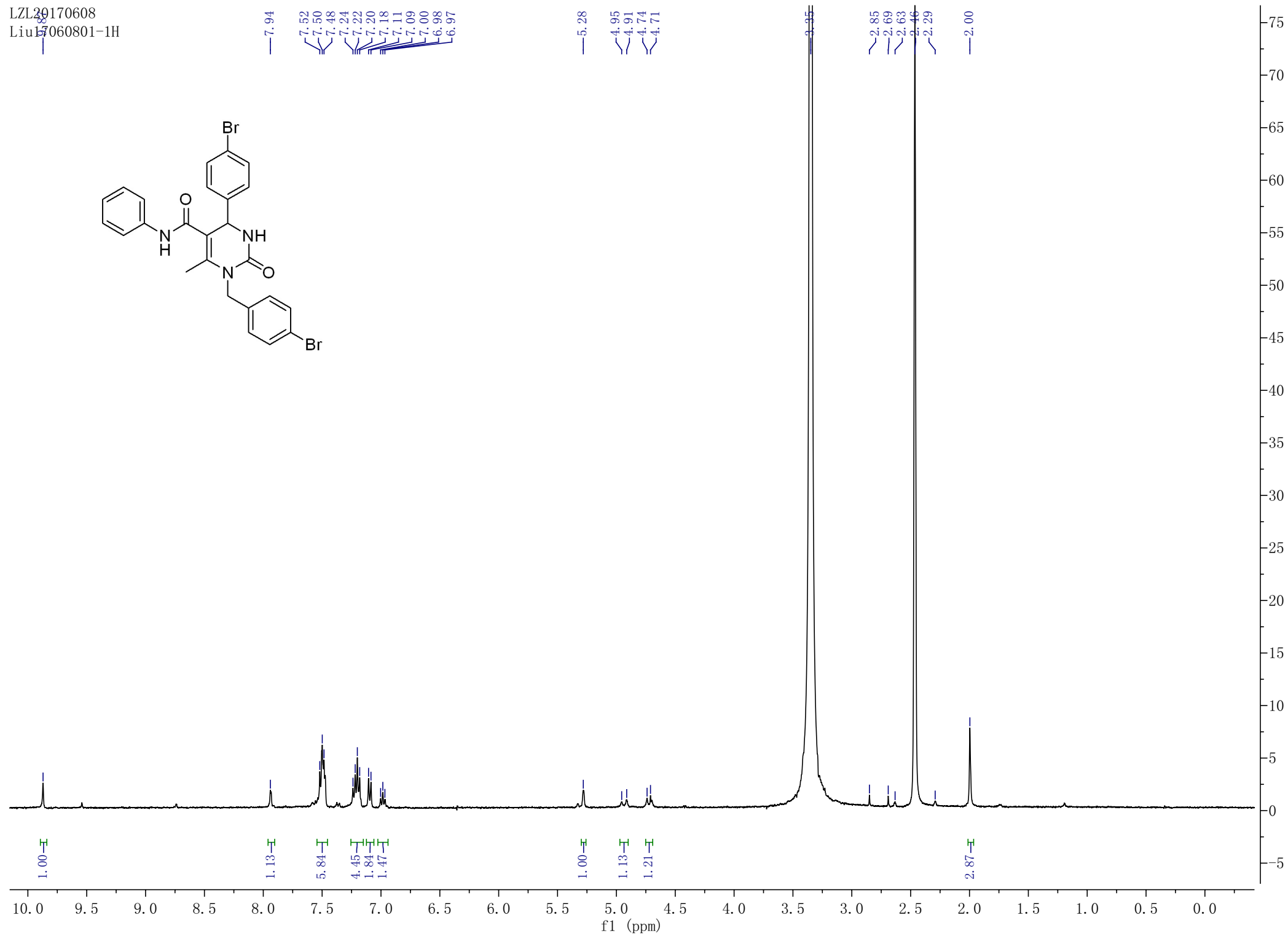

Supplement: Supplementary file 1 [file molecules-24-00891-s001.zip › molecules-433653-suppl/1H-NMR/8d.pdf]

LZL20170619  
Liu17061904-1H

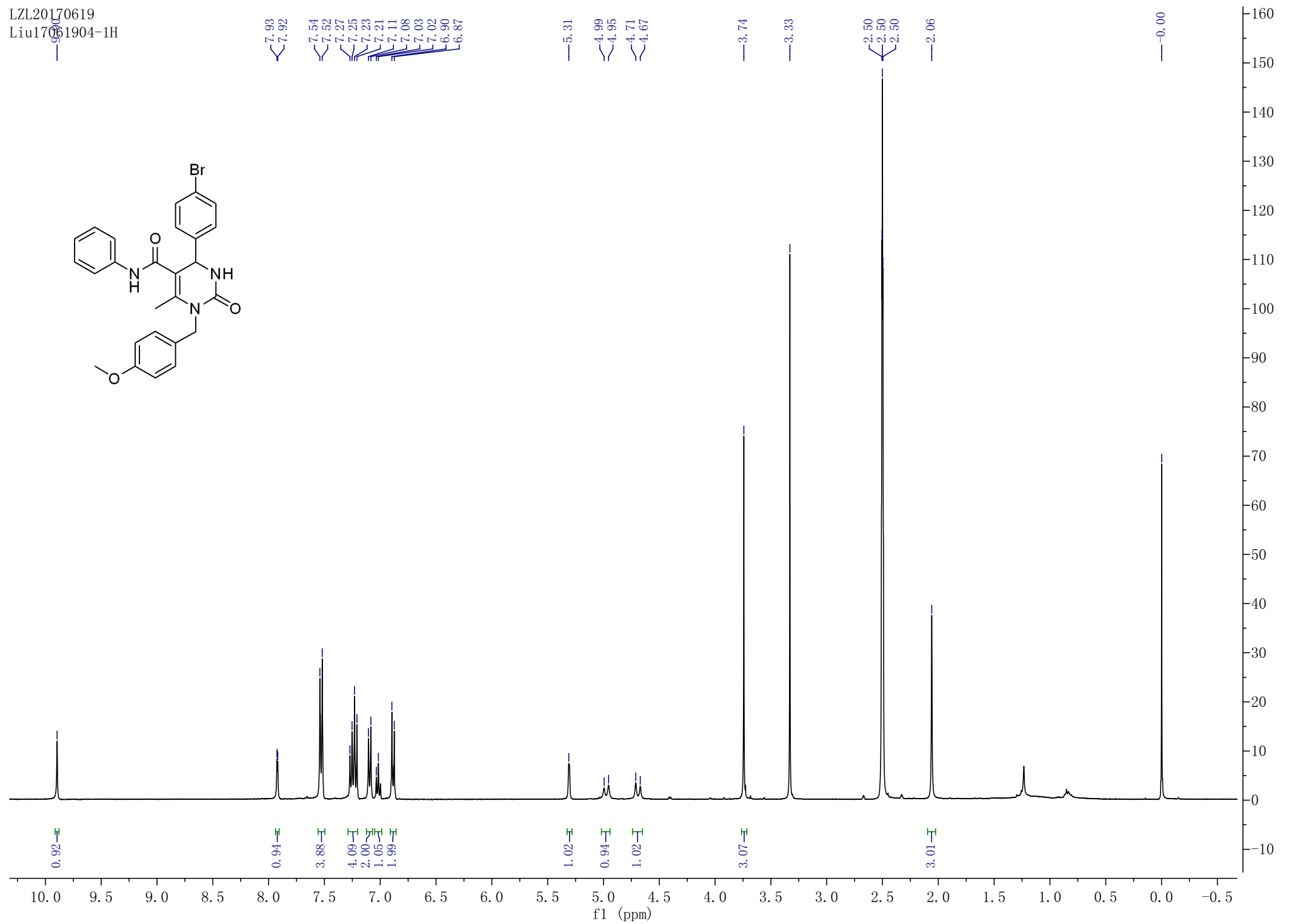

Supplement: Supplementary file 1 [file molecules-24-00891-s001.zip › molecules-433653-suppl/1H-NMR/8e.pdf]

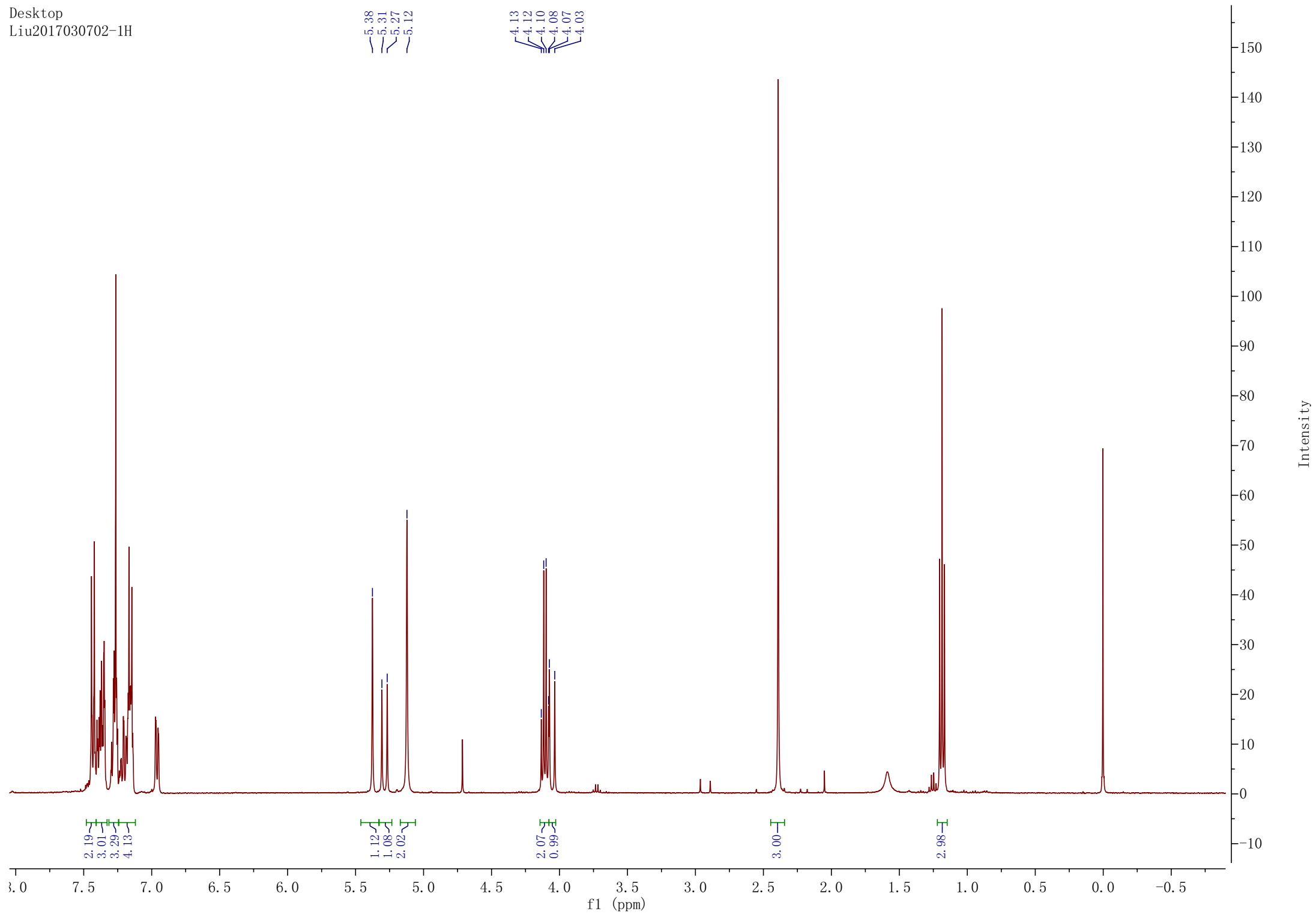

Supplement: Supplementary file 1 [file molecules-24-00891-s001.zip › molecules-433653-suppl/1H-NMR/N1 and N3 dialkylation of DHPMs.pdf]
