# Supplementary material for: Synthesis, Characterization, and Anticancer Activities Evaluation of Compounds Derived from 3,4-Dihydropyrimidin-2(1H)-one
Source: Molecules. 2019 Mar 3;24(5):891. doi: 10.3390/molecules24050891 (PMC6429579; doi:10.3390/molecules24050891)
Supplement: Supplementary file 1 [file 1i.pdf › molecules-433653-suppl/1H-NMR/1i.pdf]

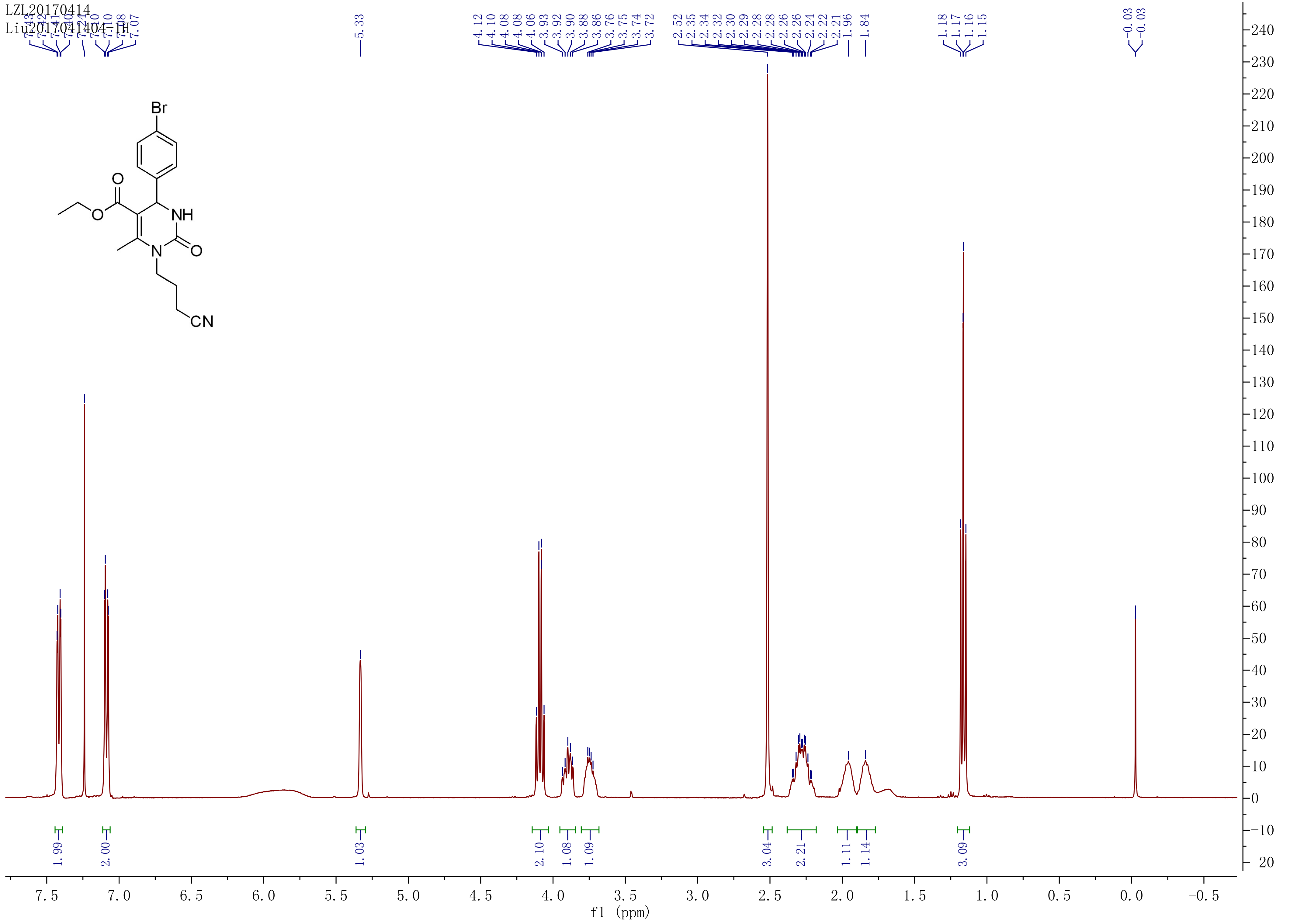
LZL20170414
Liu2017041404-1H
7.43 7.42 7.41 7.40 7.24 7.10 7.10 7.08 7.07
5.33
4.12 4.10 4.08 4.08 4.06 3.93 3.92 3.90 3.88 3.86 3.76 3.75 3.74 3.72
2.52 2.35 2.34 2.32 2.30 2.29 2.28 2.28 2.26 2.26 2.24 2.22 2.21 1.96 1.84
1.18 1.17 1.16 1.15
0.03 -0.03
Br
O
O
NH
N
O
CN
1.99 2.00 1.03 2.10 1.08 1.09 3.04 2.21 1.11 1.14 3.09
f1 (ppm)
